# Multilayered regulation of developmentally programmed pre-anthesis tip degeneration of the barley inflorescence

**DOI:** 10.1093/plcell/koad164

**Published:** 2023-06-07

**Authors:** Nandhakumar Shanmugaraj, Jeyaraman Rajaraman, Sandip Kale, Roop Kamal, Yongyu Huang, Venkatasubbu Thirulogachandar, Adriana Garibay-Hernández, Nagaveni Budhagatapalli, Yudelsy Antonia Tandron Moya, Mohammed R Hajirezaei, Twan Rutten, Götz Hensel, Michael Melzer, Jochen Kumlehn, Nicolaus von Wirén, Hans-Peter Mock, Thorsten Schnurbusch

**Affiliations:** Leibniz Institute of Plant Genetics and Crop Plant Research (IPK), Corrensstr. 3, OT Gatersleben, Seeland 06466,Germany; Leibniz Institute of Plant Genetics and Crop Plant Research (IPK), Corrensstr. 3, OT Gatersleben, Seeland 06466,Germany; Leibniz Institute of Plant Genetics and Crop Plant Research (IPK), Corrensstr. 3, OT Gatersleben, Seeland 06466,Germany; Leibniz Institute of Plant Genetics and Crop Plant Research (IPK), Corrensstr. 3, OT Gatersleben, Seeland 06466,Germany; Leibniz Institute of Plant Genetics and Crop Plant Research (IPK), Corrensstr. 3, OT Gatersleben, Seeland 06466,Germany; Leibniz Institute of Plant Genetics and Crop Plant Research (IPK), Corrensstr. 3, OT Gatersleben, Seeland 06466,Germany; Leibniz Institute of Plant Genetics and Crop Plant Research (IPK), Corrensstr. 3, OT Gatersleben, Seeland 06466,Germany; Leibniz Institute of Plant Genetics and Crop Plant Research (IPK), Corrensstr. 3, OT Gatersleben, Seeland 06466,Germany; Leibniz Institute of Plant Genetics and Crop Plant Research (IPK), Corrensstr. 3, OT Gatersleben, Seeland 06466,Germany; Leibniz Institute of Plant Genetics and Crop Plant Research (IPK), Corrensstr. 3, OT Gatersleben, Seeland 06466,Germany; Leibniz Institute of Plant Genetics and Crop Plant Research (IPK), Corrensstr. 3, OT Gatersleben, Seeland 06466,Germany; Leibniz Institute of Plant Genetics and Crop Plant Research (IPK), Corrensstr. 3, OT Gatersleben, Seeland 06466,Germany; Leibniz Institute of Plant Genetics and Crop Plant Research (IPK), Corrensstr. 3, OT Gatersleben, Seeland 06466,Germany; Leibniz Institute of Plant Genetics and Crop Plant Research (IPK), Corrensstr. 3, OT Gatersleben, Seeland 06466,Germany; Leibniz Institute of Plant Genetics and Crop Plant Research (IPK), Corrensstr. 3, OT Gatersleben, Seeland 06466,Germany; Leibniz Institute of Plant Genetics and Crop Plant Research (IPK), Corrensstr. 3, OT Gatersleben, Seeland 06466,Germany; Leibniz Institute of Plant Genetics and Crop Plant Research (IPK), Corrensstr. 3, OT Gatersleben, Seeland 06466,Germany; Faculty of Natural Sciences III, Martin Luther University Halle-Wittenberg, Institute of Agricultural and Nutritional Sciences, Halle 06120,Germany

## Abstract

Leaf and floral tissue degeneration is a common feature in plants. In cereal crops such as barley (*Hordeum vulgare* L.), pre-anthesis tip degeneration (PTD) starts with growth arrest of the inflorescence meristem dome, which is followed basipetally by the degeneration of floral primordia and the central axis. Due to its quantitative nature and environmental sensitivity, inflorescence PTD constitutes a complex, multilayered trait affecting final grain number. This trait appears to be highly predictable and heritable under standardized growth conditions, consistent with a developmentally programmed mechanism. To elucidate the molecular underpinnings of inflorescence PTD, we combined metabolomic, transcriptomic, and genetic approaches to show that barley inflorescence PTD is accompanied by sugar depletion, amino acid degradation, and abscisic acid responses involving transcriptional regulators of senescence, defense, and light signaling. Based on transcriptome analyses, we identified *GRASSY TILLERS1* (*HvGT1*), encoding an HD-ZIP transcription factor, as an important modulator of inflorescence PTD. A gene-edited knockout mutant of *HvGT1* delayed PTD and increased differentiated apical spikelets and final spikelet number, suggesting a possible strategy to increase grain number in cereals. We propose a molecular framework that leads to barley PTD, the manipulation of which may increase yield potential in barley and other related cereals.

## Teaser

A molecular framework of barley pre-anthesis tip degeneration involves complex, multilayered processes related to senescence, autoimmunity/defense, and light regulation affecting final grain number.

**IN A NUTSHELL**

**Background:** In many cereal crops, some floret primordia develop into fertile florets that form grains, but some fail to form grains. In barley (*Hordeum vulgare* L.), arrest and cessation of the inflorescence apex are usually followed by death of apical spikelets/florets and the central axis beneath the apex during early reproductive development. Previous studies on apical floret death have proposed prominent roles for assimilate allocation, phytohormone homeostasis, and poor vascular connections; however, the underlying molecular mechanism of pre-anthesis tip degeneration (PTD) in barley remains elusive. Reducing the extent of spikelet/floret loss represents an opportunity to increase grain yield in barley and related cereals.

**Question:** Is PTD of the barley inflorescence developmentally programmed? Is the apical part of the barley inflorescence limited by primary metabolites? Does the dying apical part display distinct phytohormone patterning compared to the viable parts? What are the major gene players regulating PTD?

**Findings:** Our spatiotemporal multi-omics studies revealed that barley inflorescence PTD is accompanied by sugar depletion, amino acid degradation, and abscisic acid responses involving transcriptional regulators of senescence. Photosynthesis, inflorescence greening, and energy metabolism contribute to proper spikelet growth and differentiation and were restricted to viable parts. The transcription factor GRASSY TILLERS1 (HvGT1) was identified as a modulator of barley PTD: a *HvGT1* knockout showed delayed PTD, harbored more differentiated apical spikelets, and exhibited an increased final spikelet number. This study proposes a molecular framework for barley spike PTD, the manipulation of which may increase yield in barley and other related cereals.

**Next steps:** Loss of *HvGT1* function delayed, but did not abolish, PTD. What are the upstream regulators defining the onset and extent of PTD? Putative PTD candidates can be used to exploit their natural allelic variants, their functional validation, and multiplex editing to increase grain yield in barley and other related cereals.

## Introduction

Barley (*Hordeum vulgare* L.), along with wheat (*Triticum aestivum* L.) and rye (*Secale cereale* L.), is one of the most economically important cereal crops in the Triticeae tribe (Poaceae). Barley possesses an indeterminate “spike”-type inflorescence that forms basic floral structures, called spikelets, in a distichous pattern along its central axis (termed rachis). Each rachis node in the barley spike produces 3 (1 central and 2 lateral) spikelets. Barley spikelets are uniflorous and thus produce a single grain. Two- or six-rowed spike types are specified based on the fertility of the 2 lateral spikelets (Koppolu and Schnurbusch 2019). In both row types, the end of spikelet primordia initiation along the rachis marks the stage of maximum yield potential (MYP) (Thirulogachandar and Schnurbusch 2021). Subsequently, the inflorescence meristem (IM) dome starts to collapse, followed by gradual basipetal degeneration of spikelet primordia and spikelets until a specific position along the spike is reached (Supplemental Fig. S1). This mechanism occurs in the early stages of plant development when the leaves still cover the spikes. The MYP stage, maximal spikelet number, and pre-anthesis tip degeneration (PTD) vary among barley genotypes and ultimately influence final grain number, a key yield determinant (Kamal et al. 2021; Thirulogachandar and Schnurbusch 2021). Understanding the molecular underpinnings of spike PTD may thus ultimately help improve grain yield.

Barley spike PTD resembles floret death in wheat spikelets (Sakuma et al. 2019) and, to some extent, ear tip degeneration in maize (*Zea mays* L.), with the latter also possessing indeterminate IMs (Pei et al. 2022; Ning et al. 2021). Many studies on wheat floret death have proposed a prominent role for assimilate allocation, the developmental age of florets, and poor vascular connections (Dreccer et al. 2014; Ghiglione et al. 2008; Ferrante et al. 2013; Wolde and Schnurbusch 2019). Recently, a mutation in the homeobox gene *GRAIN NUMBER INCREASE1* (*GNI-A1*) was reported to increase grain number by increasing floret fertility in wheat (Sakuma et al. 2019). Few morphometric or genetic studies have been conducted to dissect barley spikelet fertility/survival and grain number determination (Alqudah and Schnurbusch 2014; Kamal et al. 2021; Thirulogachandar et al. 2021). Nevertheless, several flowering time genes, such as *FLOWERING LOCUS T1, 2 and 4* (*FT1,2 &4*), *PHOTOPERIOD1* (*PPD-H1*), and *CENTRORADIALIS* (*CEN*), have been reported to regulate spikelet initiation, fertility, and grain yield (Bi et al. 2019; Digel et al. 2015; Pieper et al. 2020; Shaw et al. 2019). So far, the molecular mechanism and factors controlling spike PTD in barley and wheat floret death remain elusive. Moreover, spike PTD occurs in the early stages of plant development, which requires microscopy analysis to track the exact MYP stage and the onset of spike PTD, making a systemic description and analysis more challenging.

Age-induced tissue degeneration can affect the entire plant, whole organs, or tissues, including meristems (Bleecker and Patterson 1997; Gan 2003; Van Hautegem et al. 2015). Of these levels, leaf senescence is the most widely studied and involves multiple endogenous and environmental factors (Woo et al. 2019). Phytohormonal, nutritional, and transcriptional regulations are the most well-characterized factors for their role in senescence (Breeze et al. 2011; Balazadeh et al. 2008; Schippers et al. 2015). Genes regulating leaf senescence in barley and wheat are known (Borrill et al. 2019; Christiansen and Gregersen 2014; Krupinska et al. 2002; Uauy et al. 2006), as are recently identified molecular determinants involved in barley root senescence (Liu et al. 2019). However, we lack a comprehensive chronological study that describes the morphological, biochemical, and transcriptional changes occurring during the barley spike growth phase that are necessary to reach a better understanding of PTD.

In the present study, we performed spatiotemporal profiling of the transcriptome, plant hormones, and primary metabolites by dividing spikes of 2- and 6-rowed barley cultivars into degenerating apical and viable central and basal regions at stages before and during the onset of PTD over a 2-wk period. While photosynthesis, spike greening, and energy metabolism appear to be significant contributors to proper spikelet growth and differentiation and were restricted to basal and central parts, we discovered that the degenerating apical spike region undergoes sugar depletion and amino acid (AA) degradation along with enhanced abscisic acid (ABA) biosynthesis and signaling. Notably, the most common putative candidate genes for spike PTD included regulators of senescence, defense responses, and growth repressors. Moreover, we functionally validated one of the apically expressed transcription factor (TF) genes, *GRASSY TILLERS1* (*HvGT1*), an ortholog of maize *GT1* and a homolog of wheat *GNI-A1*, as a growth repressor of apical spikelet development. Targeted mutation of *HvGT1* in 2-rowed barley delayed the onset of spike PTD and produced more differentiated apical organs, resulting in significantly more fertile spikelets/florets and increased final spikelet number.

## Results

### Spike PTD in barley is a well-orchestrated developmental process

**Figure 1.**
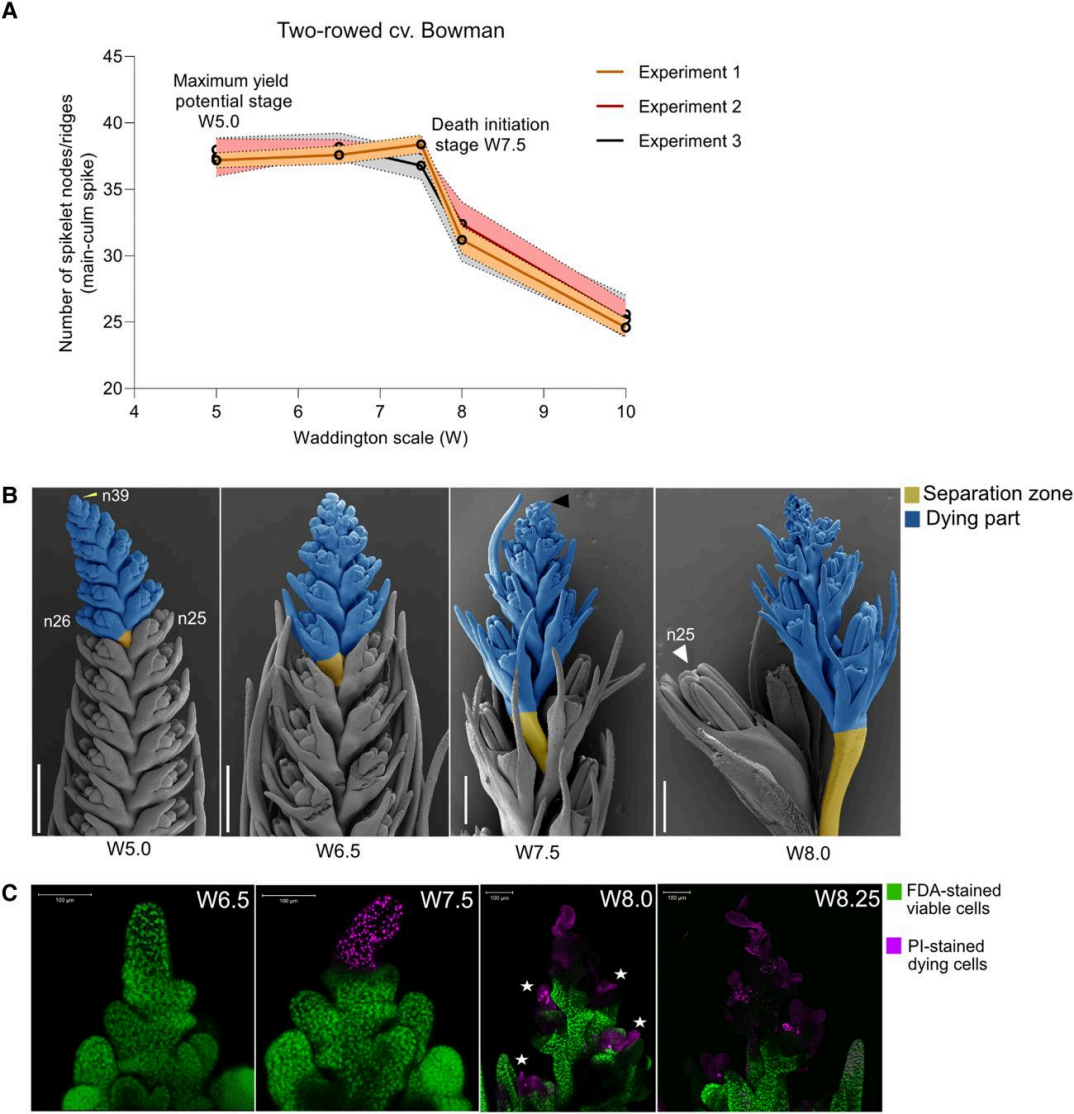
PTD in barley is a well-orchestrated developmental process. **A)** Spike developmental pattern in the 2-rowed cv. Bowman. Plots represent mean and error with a 95% confidence interval for 5 main-culm spike meristems at each developmental stages from 3 independent experiments in standardized phytochamber conditions (Materials and Methods; Supplemental Data Set S1). The *x*-axis indicates stages, and the *y*-axis shows the number of spikelet nodes/spikelet ridges produced in the main-culm spike. **B)** Representative Scanning Electron Microscope (SEM) images show suppressed growth of dying apical spikelets from the central region at 4 developmental stages between MYP and death progression in Bowman. The yellow arrow points to the final spikelet ridge (node 39) at W5.0, the black arrow points to visible initiation of death, and the white arrow points to the well-differentiated last surviving spikelet (node 25). Scale bar 500 *µ*m. n, rachis node. **C)** Live–death staining assay using fluorescein diacetate (FDA) and propidium iodide (PI) labeling in Bowman. FDA (green) stains viable cells, and cells undergoing death were stained with PI (Magenta). The star points to the death of anthers in the spikelets at stage W8.0. Scale bar 100 *µ*m. MYP, maximum yield potential; W, Waddington scale.

To better understand the sequential events leading to barley spike PTD and dissect its molecular basis, we grew plants under controlled conditions to limit possible environmental influences (see Materials and Methods). We tracked early spike development in the main culm of the 2-rowed barley cv. “Bowman” using the Waddington scale (Waddington et al. 1983) and determined the MYP stage using the “spikelet stop” method (Thirulogachandar and Schnurbusch 2021). Importantly, the developing inflorescence showed robust and reproducible developmental patterns in terms of spikelet initiation and degeneration (Fig. 1A and Supplemental Data Set S1). Under these standardized growth conditions, each Bowman spike produced 38 to 39 rachis nodes/ridges at stage W5.0 (38 d after sowing [DAS]), of which 13 to 14 aborted, reaching a spikelet survival rate ([final spikelet number/potential spikelet number] × 100) of about 64%. At the MYP stage (W5.0), spikes displayed a lanceolate phenotype with an acropetal developmental gradient of spikelets (advanced spikelets toward the basal part). Developmental differences became more evident between spikelets from the degenerating apical region and those from the viable central and basal regions as growth progressed after the MYP stage (Fig. 1B).

With the onset of spike elongation at W6.5 (44 DAS) (pre-death stage), the spike shape became more oblong with a higher degree of developmental synchronicity between the spikelets with central and basal positions. However, spikelets and spikelet primordia at the spike tip still showed a developmental gradient until the visible collapse of the inflorescence dome (visible death initiation stage), which takes place around W7.5 (48 DAS) (Fig. 1B). These observations indicate that the fate of the apical and destined-to-die spikelets and spikelet primordia is determined prior to visible signs of this decision. We used PI–FDA (propidium iodide–fluorescein diacetate) staining to distinguish between live and dead tissues, which clearly showed that the initiation of cell death starts at W7.5 from the inflorescence dome and progresses basipetally at stage W8.0 (52 DAS). Typically, anthers died before other tissues were affected (Fig. 1C). These observations suggest early spike development and PTD in barley main-culm spikes respond to strict developmental control for a given genotype when using defined growth conditions.

### Differential gradients of growth regulators distinguish the fate of apical spikelets

**Figure 2.**
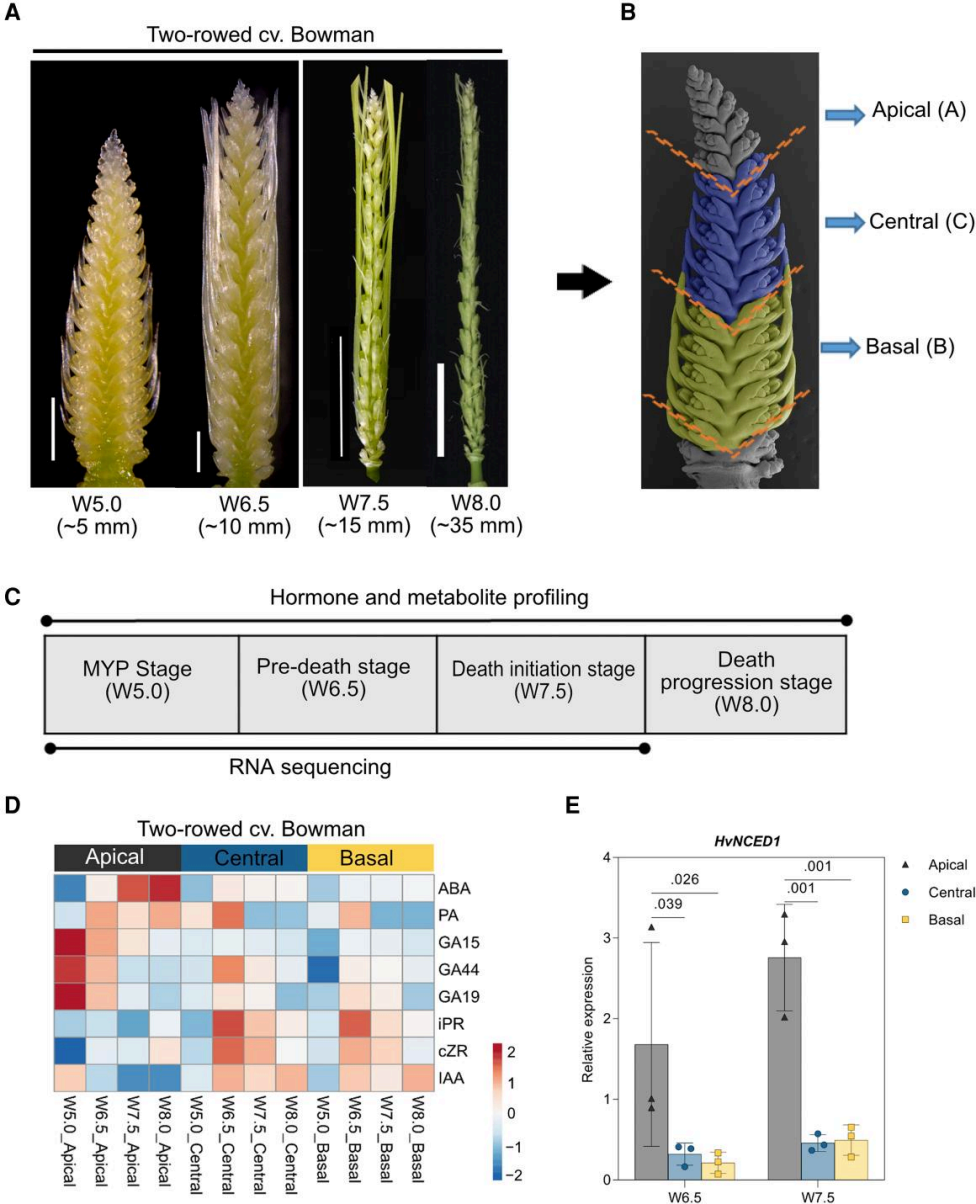
Influence of spike hormonal patterning on barley PTD. **A)** Four spike developmental stages within spike growth phase of cv. Bowman considered in this study. Values under the stages represents the approximate length of the spikes at each developmental stage. Scale bar 1 mm (W5.0, W6.5); 1 cm (W7.5, W8.0). **B)** Representative SEM image of spike depicting the separation into 3 parts apical (A, grey), central (C, blue), and basal (B, yellow). **C)** Table showing the description of stages used for hormones, metabolome (all 4 stages), and transcriptome profiling (first 3 stages until death initiation). **D)** Heatmap depicts the hormonal distribution along with the 3 spike positions of Bowman and Morex. The column annotation indicates the spike stages and positions, whereas the row annotation indicates the measured hormones. The color scale represents the ln(*X* + 1)-transformed hormonal concentrations from means calculated from at least 3 to 4 biological replicates with color codes: red, high; blue, low amounts (Materials and Methods). A, apical; C, central; B, basal. **E)** Bar plots show the relative expression (RT-qPCR) of ABA biosynthesis gene *HvNCED1* in the 2-rowed Bowman. Plots show means ± Sd from 3 biological replicates (Materials and Methods). Significance was tested by 2-way ANOVA with Tukey's multiple comparison test to evaluate the difference between apical, central, and basal positions at each stage (Supplemental Data S41). MYP; maximum yield potential; W, Waddington scale; SEM, scanning electron microscope; cv., cultivar; ABA, abscisic acid; PA, phaseic acid; GA, Gibberellic acid; iPR, *N*^6^-(Δ^2^-isopentenyl) adenine 9-riboside; cZR, *cis*-zeatin 9-riboside; IAA, indole acetic acid; *HvNCED1*, barley *NINE-cis-EPOXYCAROTENOID DIOXYGENASE1*.

Previous findings showed differential accumulation patterns of auxin, cytokinin, and gibberellins along barley spikes (Youssef et al. 2017). We thus hypothesized that phytohormonal patterning along an inflorescence might act as a signal that regulates apical spikelet/rachis development and death. Indeed, endogenous phytohormone measurements at 4 early developmental stages (W5.0 to W8.0) in 3 different spike parts (Fig. 2, A to C) revealed that ABA steadily increased in the apical parts under controlled conditions (phytochamber) (see Materials and Methods; Fig. 2D and Supplemental Fig. S2A). Notably, higher apical ABA levels (∼3-fold compared to viable central and basal parts) coincided with the arrest of the inflorescence dome (W7.5), and these levels continued to increase (to ∼4-fold compared to viable central and basal parts) at the death progression stage (W8.0). We observed similar patterns for the ABA catabolite phaseic acid (PA) (Fig. 2D), which has ABA–like functions in Arabidopsis (*Arabidopsis thaliana*) (Weng et al. 2016). Reverse transcription quantitative PCR (RT-qPCR) analysis detected higher expression levels for the key ABA biosynthesis gene *HvNCED1* (*NINE-cis-EPOXYCAROTENOID DIOXYGENASE1*) in apical parts compared to viable central and basal parts (Fig. 2E). These results indicate that a higher accumulation of ABA and its catabolite PA in the apical part of the spike accompany growth repression and death of apical organs.

In contrast to ABA, the growth-promoting phytohormones auxin (indole-acetic acid [IAA]) and cytokinin (CK) ribosides (iPR, *N*^6^-(Δ^2^-isopentenyl) adenine 9-riboside; and cZR, *cis*-zeatin 9-riboside) accumulated in the central and basal parts of spikes (Fig. 2D and Supplemental Fig. S2A). This pattern was maintained for IAA in all 4 stages tested, which is consistent with its essential role in meristem growth and differentiation (Yamaguchi et al. 2013; Wang and Jiao 2018). By contrast, IAA levels remained low in the apical parts after W5.0, consistent with poor differentiation of apical organs. Interestingly, we measured a sharp increase for iPR and cZR levels in the central and basal parts of stage 2 spikes (W6.5), followed by a drop in later stages. The gibberellic acid (GA) precursors GA15, GA44, and GA19 accumulated at high levels in the apical part at W5.0 and declined over time (Fig. 2D and Supplemental Fig. S2A), consistent with a role for GA in barley floral induction (Boden et al. 2014). Both GA44 and GA19 levels were high at W6.5 in the central and basal positions, coinciding with spike elongation and spikelet differentiation; their levels later decreased. These patterns in phytohormones were relatively conserved in samples collected from plants grown under long-day conditions in the greenhouse rather in the phytochamber (Supplemental Fig. S2B). Overall, the observed auxin and CK patterns clearly indicate that the levels of these phytohormones are higher in the central and basal parts of spikes to support their continuing growth and differentiation, whereas PTD is highly associated with ABA and PA levels.

### Dying apical parts display contrasting patterns of primary metabolites

Metabolic and energy homeostasis strongly influences plant cell growth, viability, and death (Kabbage et al. 2017; Skylar et al. 2011). We postulated that primary metabolism is altered in the dying apical part based on previous findings on apical floret fertility in wheat (Ghiglione et al. 2008). AAs can also function as signaling molecules in plants and for metabolic adaptation in response to stress (Heinemann and Hildebrandt 2021). To determine metabolite distribution at high spatial resolution, we subjected median longitudinal cryosections of Bowman spikes to MALDI (matrix-assisted laser desorption ionization) mass spectrometry imaging (MSI), a spatial metabolomics technique used to map metabolites in tissue sections (Dong et al. 2016).

**Figure 3.**
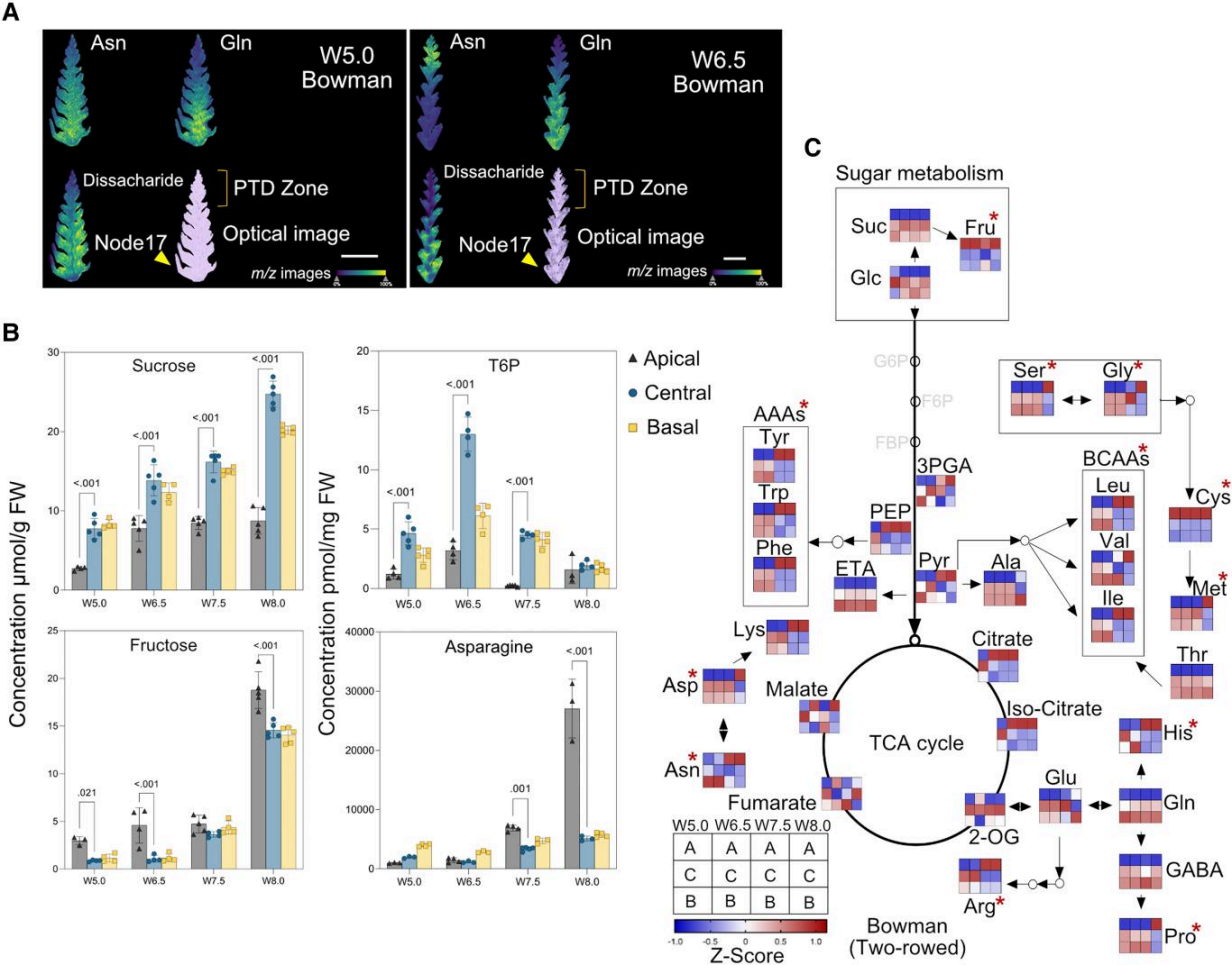
Dying apical parts display contrasting patterns of primary metabolites. **A)** Metabolite distributions in spike tissue sections of cv. Bowman by matrix-assisted laser desorption ionization mass spectrometry (MALDI-MS) imaging. Low levels of glutamine (Gln, *m/z* 147.1 ± 0.1 Da) and disaccharide (*m/z* 381.1 ± 0.1 Da) sugar towards the apical part can be observed in both W5.0 and W6.5 stages. A high level of asparagine (Asn, *m/z* 133.1 ± 0.1 Da) was found in the apical part of the section at stage W6.5. Scale bar 1 mm. The color bar shows the signal intensity of ions. **B)** Graphs show the absolute levels of soluble sugars (sucrose, fructose), sugar phosphate T6P, and AA Asparagine in different spike parts of Bowman. Plots show means ± Sd calculated from 3 to 5 biological replicates (Materials and Methods; Supplemental Data Set S2). Significance was tested using 2-way ANOVA with Tukey's multiple comparison test to evaluate the difference between apical, central, and basal positions at each stage (Supplemental Data Set S42). The *y*-axis represents absolute units of measurement. FW, fresh weight. **C)** Heatmap of primary metabolite changes during the spike growth phase. Colors represent the Z-transformed ratios of apical, central, and basal parts at each stage. Suc, sucrose; Glc, glucose; Fru, fructose; T6P, trehalose-6-phosphate; Asn, asparagine; Asp, aspartate; Arg, arginine; Ala, Alanine; Glu, glutamate; Gln, glutamine, Cys, cysteine, Met, methionine; Gly, glycine; Ser, serine; His, histidine; Val, valine; Leu, Leucine; Ile, isoleucine; Phe, phenylalanine; Tyr, tyrosine; Trp, tryptophan, Thr, threonine; Lys, lysine; GABA, gamma-aminobutyric acid; Pro, proline; 3-PGA, 3-Phosphoglyceric acid; PEP, phosphoenolpyruvate; Pry, pyruvate; 2-OG, 2-Oxoglutarate; AAAs, aromatic amino acids; BCAAs, branched-chain amino acids; TCA, tri-carboxylic acid; W, Waddington scale; cv., cultivar; PTD, pre-anthesis tip degeneration. The red asterisk points to the AAs and sugar enriched at high levels in the apical part during PTD.

We detected ion signals corresponding to sugar (disaccharide, *m/z* 381.1 ± 0.1 Da) and AAs (asparagine, Asn with *m/z* 133.1 ± 0.1 Da; glutamine, Gln with *m/z* 147.1 ± 0.1 Da) with a 15-*µ*m lateral resolution (Fig. 3A and Supplemental Table S1). In the dying apical part of spikes, the ion signals for sugar and Gln were weak in all the stages investigated, while the signal for Asn, which was lowest at W5.0, became much more substantial at W6.5 and W7.5 (Figure 3A and Supplemental Fig. S3A). Since we successfully annotated only a few of the primary metabolites detected by MALDI-MSI, we quantified soluble sugars, AAs, glycolysis, and tricarboxylic acid (TCA) cycle intermediates in dissected spike parts at similar stages used for phytohormone profiling by additional analytical approaches (see Materials and Methods; Fig. 2, A to D, and Supplemental Data Set S2).

Sucrose (Suc), a vital energy metabolite and signaling molecule for plant metabolism and growth (Wind et al. 2010), might be critical to support spike growth, as we observed that its levels increase over time in non-dying parts (central and basal) but stopped rising in dying apical parts after W6.5 (Figure 3, B and C). Trehalose-6-phosphate (T6P), which acts as a signaling metabolite in multiple aspects of plant development linking sucrose metabolism (Eveland and Jackson 2012; Figueroa and Lunn 2016), was present in significantly lower amounts in apical tissues relative to central and basal spike regions (Fig. 3B). By contrast, fructose (Fru) and trehalose (Tre) contents increased in dying apical tissues (W8.0) (Fig. 3B and Supplemental Fig. S3B).

In terms of AA distribution, we detected the highest concentration for all 3 aromatic AAs (AAAs), phenylalanine (Phe), tyrosine (Tyr), and tryptophan (Trp), which serve as precursors for a diverse class of primary and specialized metabolites (Lynch and Dudareva 2020), at W7.5 and W8.0 stages in apically dying parts (Fig. 3C). We observed a similar pattern for the 2 branched-chain amino acids (BCAAs) leucine (Leu) and isoleucine (Ile), which act as alternate respiratory substrates under energy-limited conditions (Peng et al. 2015). By contrast, another BCAA, valine (Val), and stress-responsive proline (Pro) (Alvarez et al. 2022) accumulated in the apical part only at W8.0 (Figure 3C). We also detected high lysine (Lys) and histidine (His) levels from W7.5 onward in the dying parts. Among vital AAs with a high N:C ratio, Asn (Gaufichon et al. 2010) steadily increased ∼5-fold in the apical part compared to viable central and basal parts at W8.0 and was the most abundant among the free AA pool (Fig. 3, B and C). By contrast, the content of Gln, a central metabolite of plant nitrogen metabolism (Chellamuthu et al. 2014), was lower in the apical parts (Fig. 3C), which might limit the proper growth of apical spikelets as its levels were higher in the other spike parts, which undergo normal differentiation.

Additionally, we noticed differential accumulation patterns of other TCA and glycolysis intermediates (Fig. 3C). Thus, the apical part of the spike accumulates low levels of certain key carbon and nitrogen metabolites and undergoes specific metabolic reprogramming that distinguishes it from the nondying central and basal parts to regulate spike PTD.

### Spatiotemporal transcriptome analysis uncovered molecular signatures that define the opposing developmental fates of degenerating and viable regions

**Figure 4.**
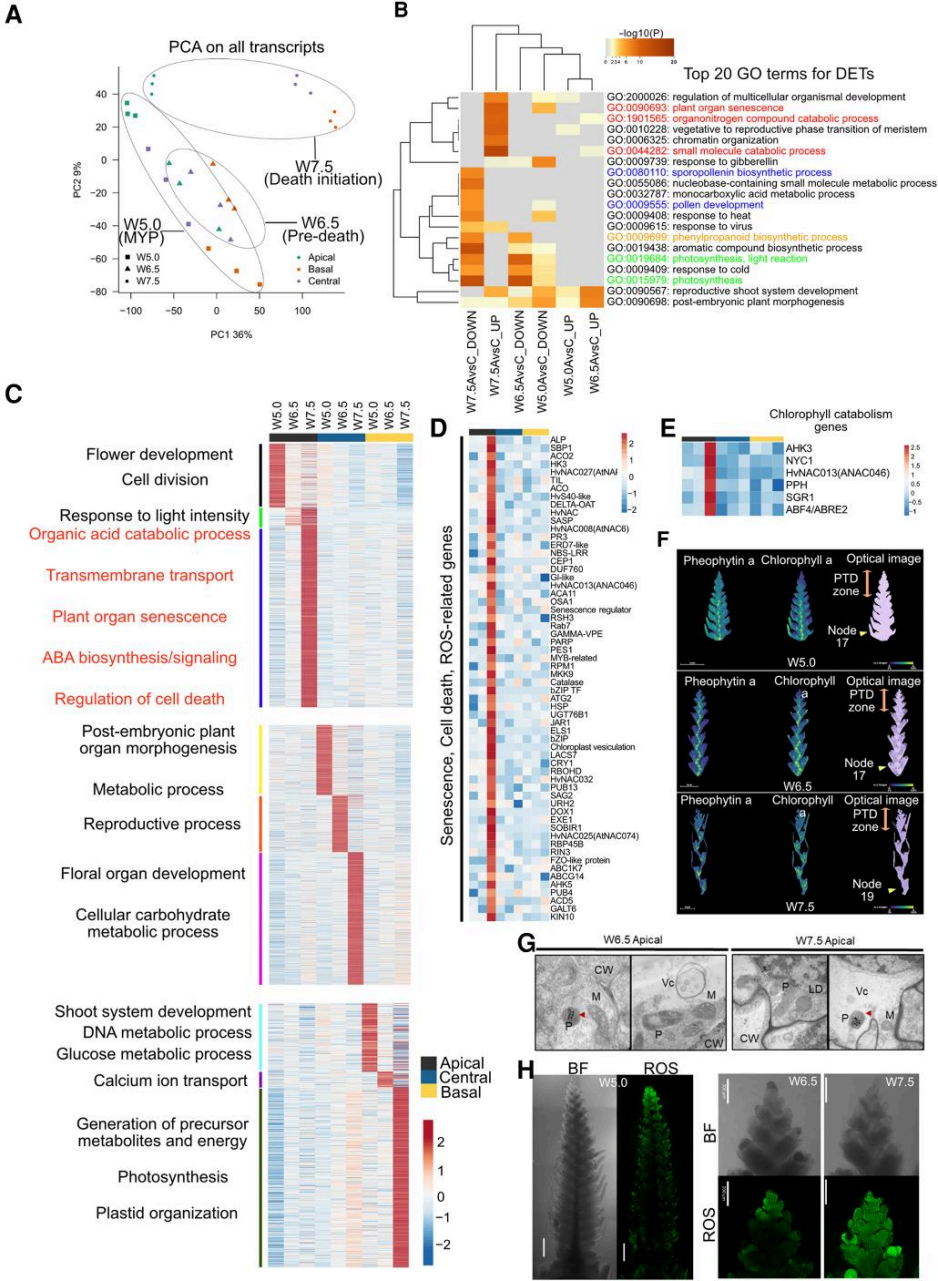
Position-specific transcriptomic analysis identifies processes differentiating viable and degenerating spike parts. **A)** Principal component analysis (PCA) of normalized expression levels (counts per million [cpm]) of all expressed transcripts. **B)** Top 20 GO terms enriched for the differential expressed transcripts (DETs) between degenerating apical and viable central parts in Bowman. Color saturation corresponds to the degree of enrichment, and the terms were hierarchically clustered based on default settings in Metascape (http://metascape.org). Highlighted GO terms represent those involved in senescence/catabolic processes (red) and those involved in growth and development (blue, orange, and green). **C)** Heatmap of apical, central, and basal position-specific transcripts identified by entropy-based ROKU analysis and stage-wise representative significant biological process. Biological processes associated with spike PTD were highlighted in red. **D)** Expression heatmap of apically enriched senescence, cell death, ROS–related genes. **E)** Expression heatmap of apically enriched chlorophyll catabolism genes. **F)** In situ visualization of chlorophyll *a* and pheophytin *a* in spike tissue sections of cv. Bowman stages W5.0, W6.5, and W7.5 by MALDI MS imaging. The color bar shows the signal intensity of ions. Scale bar 1 mm. **G)** TEM images of longitudinal sections of Bowman spikes in the apical part at W6.5 and W7.5 stages. P, plastid; M, mitochondria; CW, cell-wall; Vc, vesicles; LD, lipid droplets. The red arrow indicates plastoglobules in the plastids. **H)** ROS staining using H_2_DCFDA fluorescence dye in Bowman spike. Scale bar 500 *µ*m. Heatmaps represent ln(*X* + 1)-transformed mean TPM values were depicted by color code: red, high; blue, low expression; MYP, maximum yield potential; W, Waddington scale; PTD, pre-anthesis tip degeneration.

We performed transcriptome deep sequencing (RNA-seq) in spike parts between W5.0 and W7.5 to assess the transcriptional reprogramming that leads to the death of the apical part (see Materials and Methods; Fig. 2, A to D). A principle component analysis showed trajectories associated with spike growth stages and a developmental gradient along the spike (Fig. 4A and Supplemental Fig. S4A and Data Sets S3 and S4). In total, 83,177 transcripts were expressed (in at least 1 sample), of which 47,220 transcripts (∼57%) were differentially regulated (DETs; log_2_ [fold-change, FC] > 1) among the 15 pairwise comparisons made within and across developmental stages (see Materials and Methods; Supplemental Fig. S4B and Data Set S5).

The number of DETs was higher (∼16%) between apical (dying) and central (viable) parts, pointing to their opposite developmental fates, compared to those between the central and basal parts (∼2%), reflecting their normal developmental progression and synchrony (Supplemental Fig. S4B and Data Set S5). Gene Ontology (GO) enrichment analysis of these DETs identified distinct biological processes differentially regulated between viable (central and basal) and degenerating apical parts (Fig. 4B and Supplemental Fig. S5 and Data Sets S6 and S7), suggesting the developed transcriptome data set may provide some fruitful insights into the mechanisms governing spike PTD and spikelet growth/differentiation.

We also subjected all expressed transcripts to an entropy-based approach (ROKU) to identify apical, central, and basal position–specific transcripts (Supplemental Data Sets S8 to S10) and filtered those differentially expressed among the 15 pairwise comparisons (log_2_FC > 1; expression ≥ 1 TPM [transcripts per million] in at least 1 tissue) resulting in 3,968 transcripts (apical—2561, central—387, and basal—1020) (see Materials and Methods; Fig. 4C and Supplemental Data Sets S9 to S11) (Kadota et al. 2006). We also ranked transcripts based on their tissue specificity (Supplemental Data Sets S8 to S11). Genes enriched in central spike parts at W5.0 and W6.5 were highly related to cell cycle, flower development, embryo development, or postembryonic morphogenesis, followed by floral organ development, carbohydrate metabolism, and xylem/phloem patterning at W7.5 (Figure 4C and Supplemental Fig. S5B and Data Sets S9 and S12). In basal parts, genes identified were enriched with processes related to the cell cycle, carbon metabolism, organ development, and postembryonic development at W5.0 and W6.5. At W7.5, genes were related to photosynthesis, plastid organization, energy metabolism, chlorophyll biosynthesis, and phenylpropanoid metabolism, in line with the substantial spikelet development and greening observed in the basal to the central part of the spike (Fig. 4C and Supplemental Fig. S5C and Data Sets S10 and S13). KEGG (Kyoto Encyclopedia of Genes and Genomes) pathway enrichment of DETs in central and basal parts across development (from W5.0 to W7.5) also showed a clear enrichment for pathways related to photosynthesis, sugar, and starch metabolism, the TCA cycle, and glycolysis (Fig. 4C and Supplemental Fig. S6, A and B, and Data Set S14). These findings underscore the significance of photosynthesis, plastid organization, and energy metabolism for the constant supply/biosynthesis of assimilates to support proper growth and differentiation of spikelets in the viable (central and basal) regions.

By contrast, apically enriched genes at W5.0 were highly associated with biological processes related to cell division, flower development, meristem maintenance, chromatin organization, auxin transport, and phytohormone metabolism. However, the expression of most of these genes declined in later stages (Fig. 4C and Supplemental Fig. S7 and Data Sets S11 and S14 to S15). Furthermore, the promoters of apically enriched genes at W5.0 were significantly enriched in cis-motifs related to the REPRODUCTIVE MERISTEM protein family (Mantegazza et al. 2014) and “GAGA” (binding target of BARLEY B RECOMBINANT/BASIC PENTACYSTEINE [BBR/BPC] family proteins), regulating flower development (Theune et al. 2019) (Supplemental Data Set S16). These data suggest that at W5.0, the apical region is still intact, displaying normal meristem development and differentiation with no signs of death.

At W6.5, DETs in the apical part were enriched in GO categories related to response to light intensity, transcriptional regulation, plant organ senescence, organic acid catabolism, and chlorophyll catabolism (Fig. 4C and Supplemental Data Sets S14 and S15). The expression of most of these genes continued to increase in W7.5 (Figure 4C and Supplemental Data Set S11). Furthermore, genes at W7.5 were highly related to ABA biosynthesis/signaling, proteolysis, AA/chlorophyll catabolism, transmembrane transport, reactive oxygen species (ROS), and lipid oxidation (Fig. 4D and Supplemental Data Set S17). Consistently, we noticed *HvNCED1* (Leymarie et al. 2008), encoding a key rate-limiting enzyme; *ABA DEFICIENT4* (*ABA4*) (Perreau et al. 2020), involved in ABA biosynthesis; and several ABA–responsive/signaling genes among apically enriched genes (Supplemental Fig. S8 and Data Set S18). Moreover, dying tissues at W7.5 showed high expression of several defense response genes, including *DORMANCY-ASSOCIATED GENE/AUXIN-REPRESSED FAMILY PROTEIN* (*HvDRM/ARP*) (Roy et al. 2020) and *HYPERSENSITIVE-INDUCED REACTION3* (*HvHIR3*) (Rostoks et al. 2003). The list of DEGs in dying tissues at W7.5 also included several kinase-encoding genes, including *STRUBBELIG-RECEPTOR FAMILY3* (*SRF3*) and *OPEN STOMATA1* (*OST1*), which participate in circadian-regulated immunity (Alcázar et al. 2010; Li et al. 2011) and ABA signaling (Acharya et al. 2013), respectively (Supplemental Data Set S11).

In green tissues like leaves/culm, the earliest changes during senescence involve degreening due to chlorophyll degradation, followed by chloroplast disintegration (Tamary et al. 2019). We observed the typical basal-to-apical gradient of chlorophyll pigmentation (less pigmentation toward the spike tip) in developing barley spikes. Interestingly, genes involved in chlorophyll catabolism, such as *STAYGREEN1* (*SGR1*), *NON-YELLOW COLOUR* (*NYC1*), *PHEOPHYTINASE* (*PPH*), and *ABA-RESPONSIVE ELEMENT BINDING FACTOR2 (AREB2)*, were also enriched in the apically dying tissues of barley spikes (Yang et al. 2014) (Fig. 4E and Supplemental Data Set S11). We confirmed the acropetal chlorophyll gradient and degradation biochemically by in situ visualization of chlorophyll *a* and pheophytin *a* by MALDI-MSI in longitudinal tissue sections (Fig. 4F). Strikingly, the histone deacetylase–like gene *CHLOROPLAST VESICULATION* (*HvCV*), a putative ortholog of Arabidopsis *CV* that is involved in stress-induced chloroplast disassembly, was also highly expressed in the spike apical part (Fig. 4D) (Wang and Blumwald 2014).

Another exciting candidate previously associated with the leaf senescence detected here was the senescence-regulator gene *HvS40-like*, whose leaf senescence–associated functions have been studied in barley and other species (Fischer-Kilbienski et al. 2010; Jehanzeb et al. 2017; Krupinska et al. 2013) (Fig. 4D and Supplemental Data Set S11). Transmission electron microscopy (TEM) analysis revealed the presence of large vacuoles, plastoglobules, vesicular bodies, lipid droplets, and loosened cell walls in the apical part of the spike (Fig. 4G). Genes participating in proteolysis, AA catabolism, and nutrient remobilization were also highly expressed in the apical part, including *PAPAIN-LIKE CYSTEINE PEPTIDASE*s (*HvPAP14* and *HvPAP20*), *RESPONSIVE TO DESICCATION19* (*RD19*), and *RD21*, as well as genes encoding senescence-associated vacuolar processing enzymes (Hollmann et al. 2014) and several transporters. Consistent with the Asn accumulation observed in the apical part, genes involved in ammonium assimilation and nitrogen remobilization, such as *ASPARAGINE SYNTHETASE*s (*HvASN1* and *HvASN5*) (Avila-Ospina et al. 2015), showed an increased expression in dying parts. Similarly, *AUTOPHAGY-RELATED* genes (*ATG8A* and *ATG18A*) were highly expressed in these tissues as well (Fig. 4D and Supplemental Data Set S11). Furthermore, genes involved in ROS metabolism, for example, those encoding catalases, peroxidases, and glutathione S-transferases, were also highly expressed (Fig. 4D and Supplemental Data Set S17). Indeed, we detected high ROS fluorescence in the apical part of the spike, as evidenced by staining with the ROS–specific dye H_2_DCFDA (Fig. 4H). These results indicate that spike PTD involves chlorophyll and cellular degradation by the expression of chlorophyll catabolism and senescence-related genes.

### TFs associated with barley spike PTD

**Figure 5.**
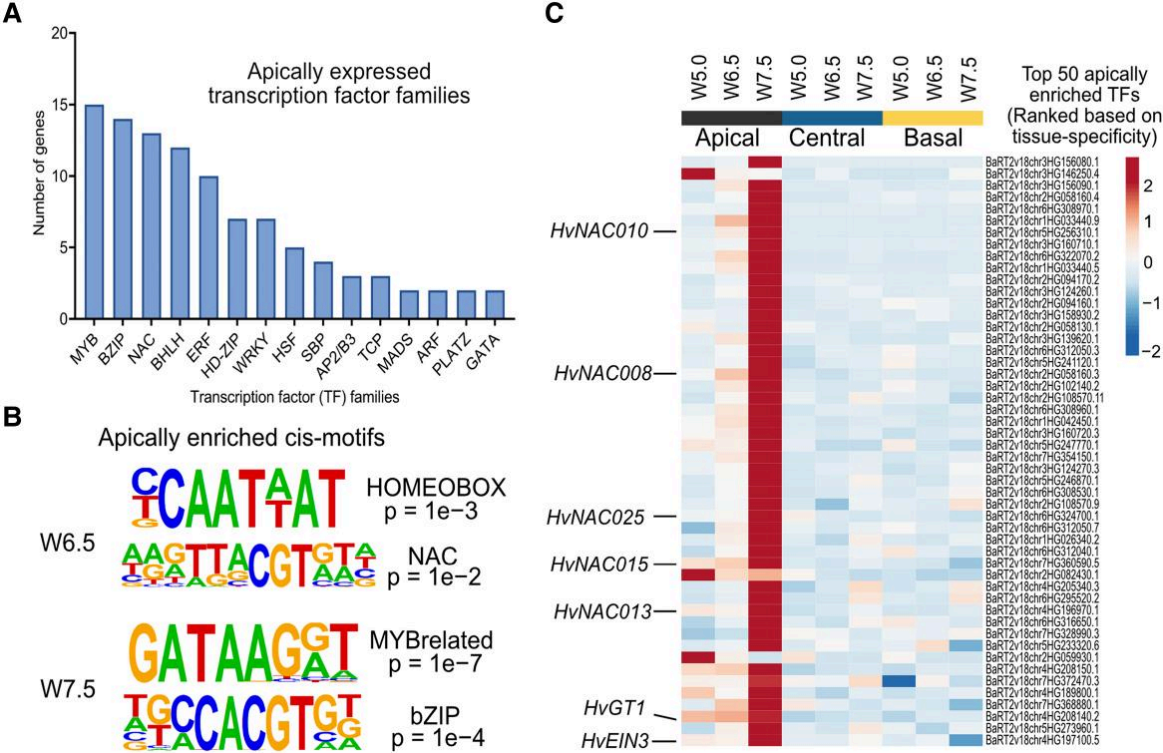
TFs associated with barley spike PTD. **A)** The bar graph represents the number of TF genes found among each TF family identified in apically enriched genes. **B)** Top cis-motifs enriched with their respective *P*-values among the PTD-associated apical genes at W6.5 (predeath) and W7.5 (death initiation) in Bowman. **C)** Expression heatmap of top 50 TF genes ranked based on apical tissue-specific expression in Bowman and highlighted some senescence-associated genes and the barley *GRASSY TILLERS1* (*HvGT1*) gene. Heatmap represents ln(*X* + 1)-transformed mean TPM values are depicted by color code: red, high; blue, low expression; W, Waddington scale; *NAC*, *(NAM [no apical meristem], ATAF1/2, CUC [cup-shaped cotyledon])*; *EIN3*, *ETHYLENE INSENSITIVE3*.

TFs tightly regulate and coordinate cell death–related processes in plants by functioning as master regulators of transcription (Borrill et al. 2019; Schippers 2015). To search for transcriptional regulators of barley spike PTD, we specifically analyzed TF genes, which represented about 9% of apically expressed PTD candidate genes (Fig. 5A and Supplemental Data Sets S19 and S20). Among those TF genes, the MYB TF family accounted for the most, followed by genes encoding bZIPs (basic LEUCINE ZIPPERs), NACs (NAM [no apical meristem], ATAF1/2, CUC [cup-shaped cotyledon]), bHLH (basic HELIX LOOP HELIX), ERFs (ETHYLENE RESPONSE FACTORs), HD-ZIPs (HOMEODOMAIN-LEUCINE ZIPPERs), and WRKYs. The promoters of apical-specific genes at W6.5 were highly enriched in HOMEOBOX and NAC binding cis-motifs (Fig. 5B and Supplemental Data Set S16). Accordingly, many genes of the NAC TF family were expressed in the apical part, most of which have been reported to function in leaf senescence (Breeze et al. 2011; Christiansen and Gregersen 2014; Christiansen et al. 2011) (Fig. 5C and Supplemental Data Sets S19 and S20). Thus, the PTD–associated expression of these NAC TF genes suggests the importance of the NAC regulatory network in the apical degeneration of barley spikes.

**Figure 6.**
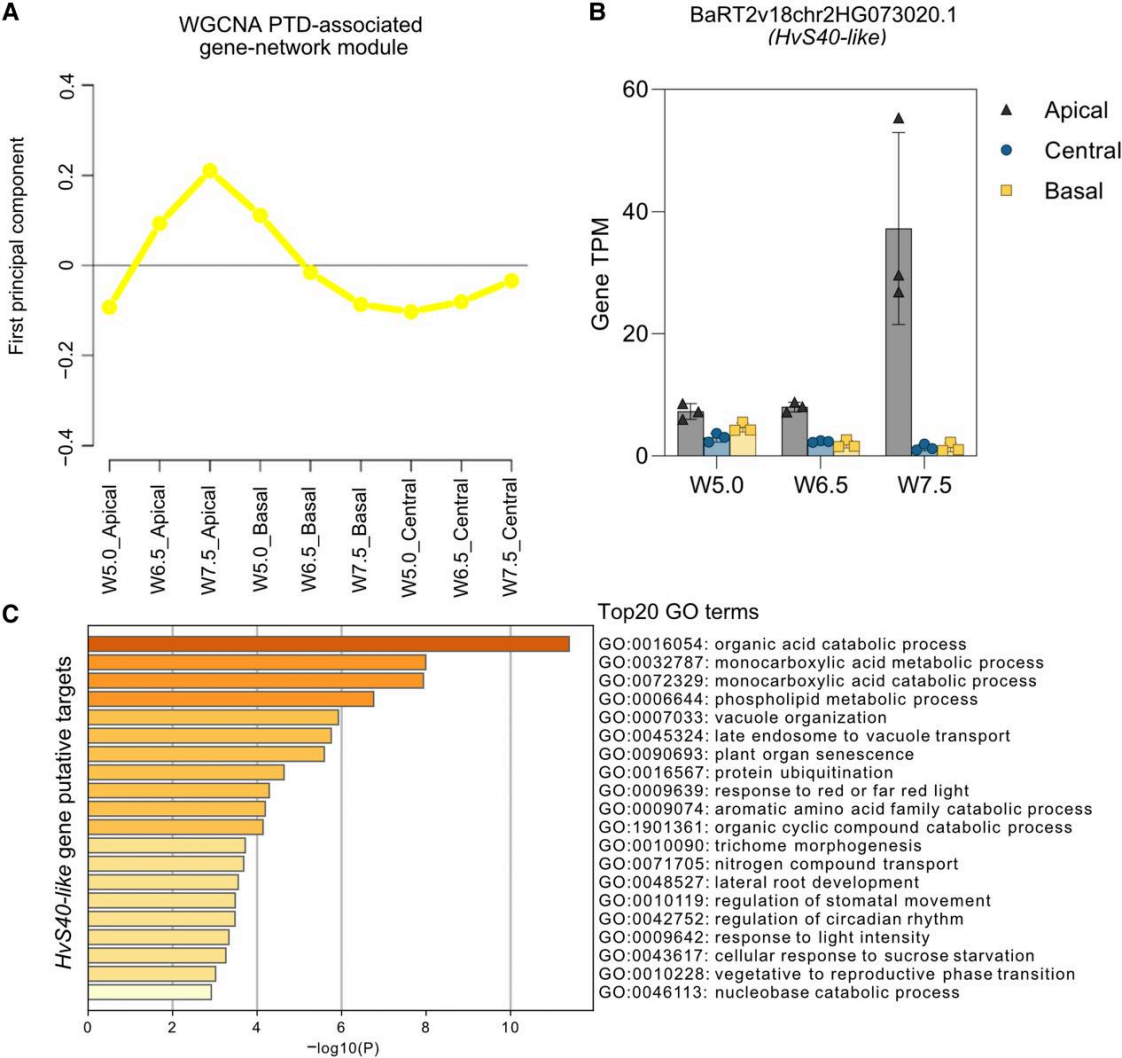
Gene coexpression network modules for the barley spike PTD. **A)** Expression shape of PTD–associated module identified in cv. Bowman (yellow) by WGCNA. **B)** Bar plots show the expression of the senescence-associated gene, *HvS40-like* (BaRT2v18chr2HG073020.1), in Bowman, which has been identified as a key hub gene in weighted gene coexpression network analysis (WGCNA). Plots show means ± Sd of TPM values from 3 biological replicates (Materials and Methods; Supplemental Data Set S4 and S21). **C)** GO term enrichment of putative targets of *HvS40-like* gene identified in Bowman. Color saturation corresponds to the degree of enrichment. W, Waddington scale; cv., cultivar; PTD, pre-anthesis tip degeneration.

We also observed the significant enrichment of MYB-related “GATAAG” and ABA–responsive element–binding (AREB/bZIP) “CACGTG” cis-motifs in apical genes at W7.5, suggesting light (Kim et al. 2016) and ABA regulation (Gómez-Porras et al. 2007), respectively, during spike PTD (Fig. 5B and Supplemental Data Set S16). We ranked TF genes based on their apical tissue specificity, which indicated that the top 50 genes include leaf senescence–associated *NAC* genes, *ERF* genes, and *HvGT1*, an ortholog of a key maize domestication gene (*GT1*) functioning in suppression of axillary tiller bud outgrowth (Whipple et al. 2011) (Fig. 5C and Supplemental Data Set S20). Additionally, we performed a weighted gene coexpression network analysis (WGCNA), which identified the leaf senescence regulator *HvS40*-like among the top 10 hub genes in the PTD-associated gene network module (see Materials and Methods; Fig. 6, A and B, and Supplemental Fig. S9, A to C, and Data Sets S21 and S22). We identified putative undirected targets of *HvS40-like* in this PTD–associated module, which included several *NAC* genes and *HvGT1*, while GO enrichment analysis of these targets suggested a role in cellular catabolism, autophagy, senescence, trehalose metabolism, and circadian rhythms (Fig. 6C and Supplemental Fig. S9C and Data Sets S23 and S24).

### HvGT1 represses apical spikelet growth

**Figure 7.**
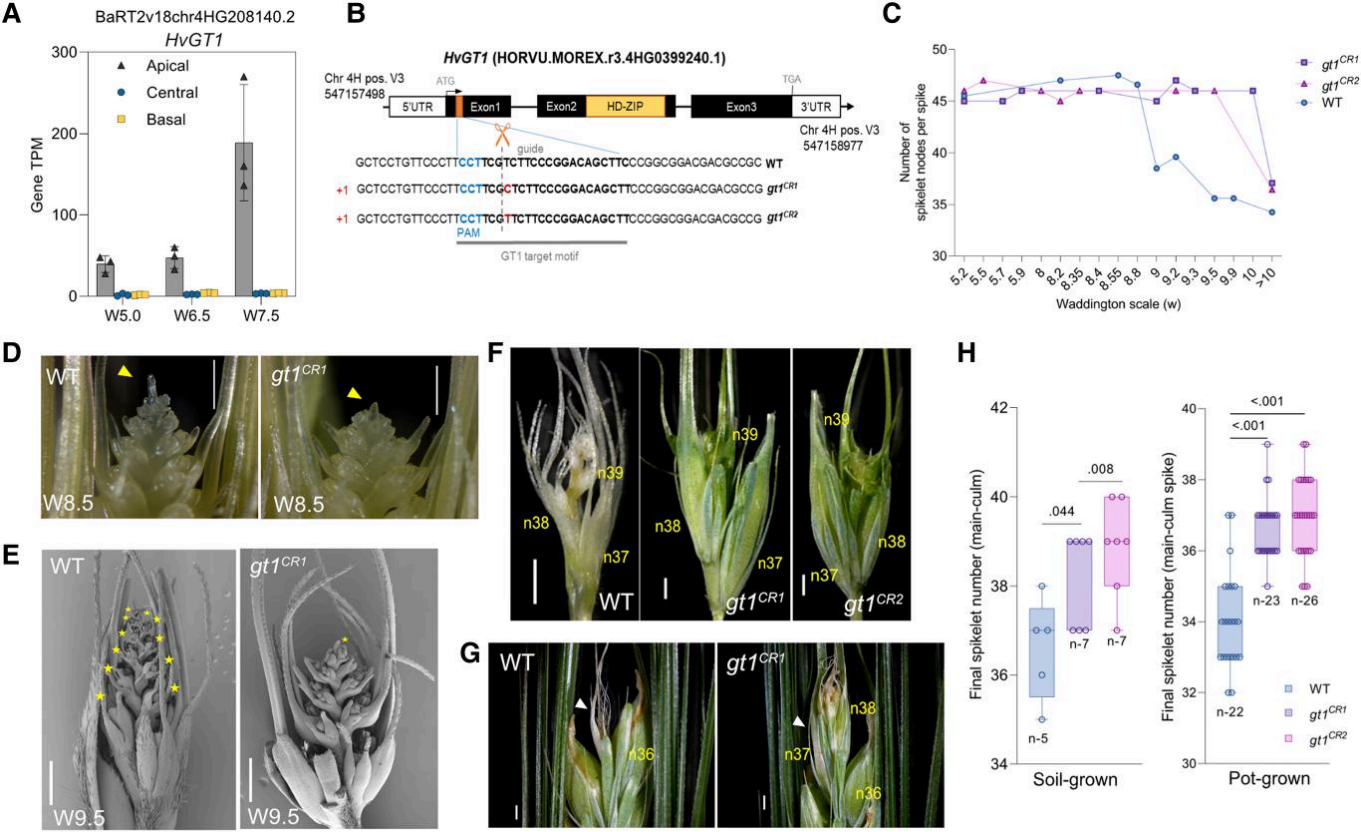
Barley *GRASSY TILLERS1 (GT1)* inhibits apical spikelet growth. **A)** Bar plot shows the increasing apical-specific expression of the PTD candidate gene *HvGT1* (BaRT2v18chr4HG208140.2) in Bowman. Plots show means ± Sd of TPM values from 3 biological replicates (Materials and Methods; Supplemental Data Set S4). **B)** Graphical representation of the guide RNA sequence with protospacer adjacent motif (PAM) and a putative cutting site (orange box in exon1) used to generate *HvGT1* CRISPR/Cas9 mutants. Black boxes and solid black lines depict gene exons and introns, respectively. The yellow box in exon2 depicts the HD-ZIP domain. **C)** Spike development pattern in WT and *gt1* mutant lines (*n* = 2 to 4 spikes for each data point until W10 and for >W10 *n* = 22 [WT], 24 [*gt1^CR1^*], 26 [*gt1^CR2^*]) grown in pots (Materials and Methods; Supplemental Data Set S27). **D)** Stereo microscope images show spike PTD in azygous WT and *gt1* mutant line (*gt1^CR1^*) in the background of 2-rowed barley cv. Golden Promise. The yellow arrow points the collapsed IM dome in WT and intact IM in *gt1* mutant. Scale bar 500 *µ*m. **E)** SEM images show the apical part of the spikes at stage W9.5 in the WT and *gt1* line (*gt1^CR1^*). Yellow stars highlight all degenerated spikelet nodes in WT and initiation of IM collapse in *gt1* mutant spike. Scale bar 500 *µ*m. **F)** Dissected dying tip of WT and well-differentiated tip of *gt1* mutant spikes (*gt1^CR1^* and *gt1^CR2^*) at heading (soil-grown). Scale bar 1 mm. **G)** Magnified view of spike tips of WT and *gt1* mutants at heading (pot-grown). The white arrow points to the degenerated tip above node 36 in the WT spike and the surviving tip of the *gt1* mutant spike. Scale bar 1 mm. n, rachis node. **H)** Box plot shows the final spikelet number at heading in the main-culm spikes of azygous WT and *gt1* mutant lines grown under greenhouse directly in soil and pots (Materials and Methods; Supplemental Data Set S27). Box plots show all individual data points with whiskers covering minimum and the maximum values; horizontal line in the box represents the median. Significant levels are determined from unpaired Student's *t*-test with Welch's correction (Supplemental Data Set S43). W, Waddington scale; PTD, pre-anthesis tip degeneration.

Among the top 50 TF genes ranked based on their apical tissue–specific expression, we identified the class I HD-ZIP gene *HvGT1* (BaRT2v18chr4HG208140/HORVU.MOREX.r3.4HG0399240.1) (Figs. 5C and 7A). An independent transcriptome atlas of the barley IM with laser-capture microdissected tissues also revealed the IM dome–specific expression of *HvGT1* at the MYP stage (Thiel et al. 2021) (Supplemental Fig. S10). Phylogenetic analysis of GT1-like proteins in barley, wheat, and maize revealed closely related proteins in the GT1 sister clades (Supplemental Fig. S11, see green and yellow sister clades, and Supplemental Data Set S25).

Repressive growth functions are known for *GRAIN NUMBER INCREASE 1* (*GNI-A1*), a wheat ortholog of the barley row–type gene *VRS1* (*six-rowed spike 1*), that is expressed in rachillae and apical florets of wheat spikelets and inhibits their growth, whereas reduced *GNI-A1* function increases floret fertility and thus, grain number (Sakuma et al. 2019). *VRS1* represses lateral spikelet development in 2-rowed barley (Komatsuda et al. 2007). In addition, maize *GT1* represses carpel development in tassel florets (Klein et al. 2022; Whipple et al. 2011). Moreover, GT1 orthologs in maize (Klein et al. 2022; Whipple et al. 2011) and Arabidopsis (HOMEOBOX PROTEIN 21 [HB21], HOMEOBOX PROTEIN 40 [HB40], and HOMEOBOX PROTEIN 53 [HB53]) (González-Grandío et al. 2017) mediate growth repression of axillary tiller buds by ABA biosynthesis and sugar depletion. In agreement with these findings, our phytohormonal and metabolite analyses detected higher ABA levels with limited sucrose and T6P levels in degenerating apical parts, pointing to the conservation of this growth repression module during barley spike PTD. In addition, we established that HvGT1-YFP is targeted to the nucleus (Supplemental Fig. 12A), suggesting a transcriptional regulation for HvGT1. Predicted targets of HvGT1 (undirected) identified by our WGCNA coexpression network analysis included the senescence regulator *HvS40-like* and other growth repressors (Supplemental Data Set S26). Hence, we focused on *HvGT1* as a candidate gene with a role in suppressing apical spikelet growth and regulating spike PTD.

To examine the HvGT1 function during barley spike PTD, we generated *Hvgt1* knockout mutants in the 2-rowed cv. “Golden Promise” (GP) using clustered regularly interspaced short palindromic repeat (CRISPR)/CRISPR–associated nuclease 9 (Cas9)–mediated gene editing (Fig. 7B and Supplemental Fig. S12B). We did not observe a phenotypic difference in main-culm spikes at the MYP stage (W5.5) between the azygous wild-type (WT) and the mutants in the T_2_ generation when grown under long-day conditions in pots or natural loamy soil, as both showed a similar number (45 to 47) of spikelets/spikelet primordia (see Materials and Methods; Fig. 7C and Supplemental Fig. S13 and Data Set S27).

Notably, substantial differences appeared at W8.5 when visible apical death was initiated in the WT but not in mutant spikes (Fig. 7D). During later stages, WT spikes displayed a basipetal degeneration of their apical spikelets (Fig. 7E and Supplemental Fig. S13B). However, apical spikelets in the *Hvgt1* mutant plants remained alive longer, and the collapse of their inflorescence dome only commenced at around W9.5, whereas apical degeneration was nearly complete in WT spikes at the same stage. In the *Hvgt1* mutant, many apical spikelets were still viable even at anthesis (W10), suggesting that the mutant undergoes a slower rate of degeneration progression. At the heading stage, the degenerated apical tip was clearly visible with dead spikelets and extra-differentiated spikelets in the mutants, compared to the completely dried tip of the WT spike (Figure 7, F and G, and Supplemental Figs. S13C and S14, A to D).

The delayed death in the *Hvgt1* mutant was accompanied by pronounced floral organ differentiation in apical spikelets, including awns, lemma, palea, stamens, and carpel, resulting in higher spikelet survival at heading and increasing the final spikelet number in both growth conditions tested (Fig. 7, F to H, and Supplemental Fig. S14, E to I, and Data Set S27). Although the final spikelet number was higher in the mutants at anthesis, the extra-surviving spikelets did not produce any regular grains in most spikes investigated. Instead, most extra spikelets remained as a rudimentary structure with empty chaff, while no significant difference was found for the final grain number per main-culm spike between WT and *Hvgt1* mutant plants grown in both growth conditions, except for the significant decrease in *gt1^CR2^* mutant grown in pots (Supplemental Fig. S15 and Data Set S27).

We did not observe differences in heading time between the WT and *Hvgt1* mutants (Supplemental Fig. S16, A and B). Additionally, we found that *Hvgt1* mutant plants produce significantly more tillers than their WT counterparts, as previously observed with *gt1* mutants in maize (Klein et al. 2022; Whipple et al. 2011) (Supplemental Fig. S16, C and D, and Data Set S27). These observations indicate that HvGT1 plays a crucial role during spike PTD in barley and that its loss can delay the onset of tip death and improve spikelet survival at heading.

### Molecular changes leading to spike PTD are conserved in 6-rowed barley

In barley, the fertility of the 2 lateral spikelets specifies spike row types: 2- and 6-rowed barley. In 6-rowed barley, all 3 spikelets per rachis node are fertile and set grains, whereas both lateral spikelets are sterile in 2-rowed barley. At least 5 genes control the lateral spikelet sterility mainly via carpel and anther abortion with growth inhibition already initiating from the triple mound stage (W2.25): *VRS1*, *VRS2*, *VRS*, *VRS4*, and *VRS5* (also named *INT-C* [*INTERMEDIUM SPIKE-C*] and *HvTB1* [*TEOSINTE BRANCHED1*]) (Koppolu and Schnurbusch 2019; Thiel et al. 2021). However, apart from this difference between 2- and 6-rowed barley, we found that barley spike PTD is a common phenomenon in both row types. Hence, we also investigated the developmental and molecular events leading to spike PTD in 6-rowed cv. Morex.

**Figure 8.**
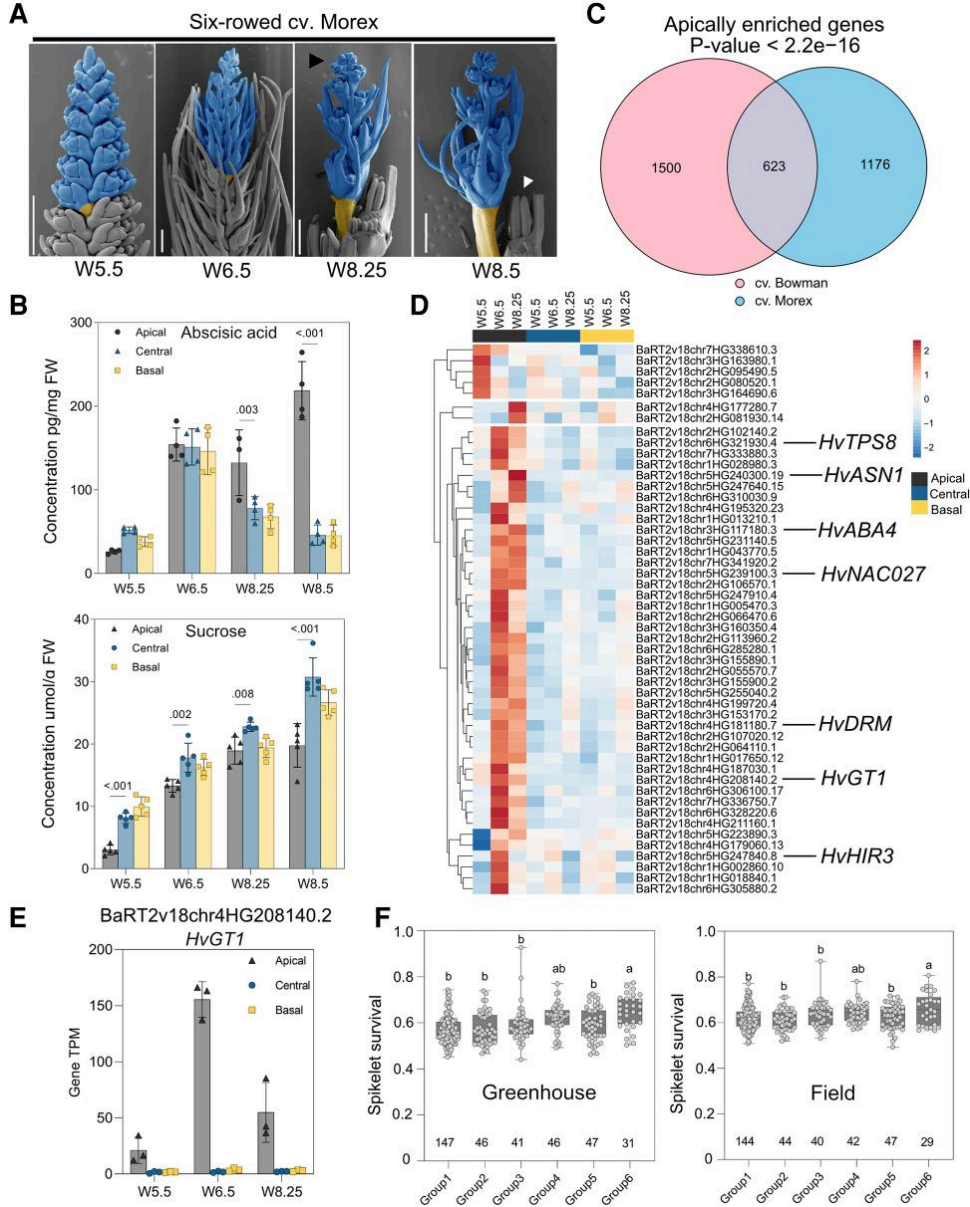
Molecular changes associated with barley spike PTD are conserved in 6-rowed barley. **A)** Representative SEM images showing the suppressed growth of dying apical spikelets compared to the central region at 4 developmental stages between MYP and death progression in 6-rowed cv. Morex. Dying spikelets are colored blue, whereas the yellow-colored internode represents the separation zone of dying and viable parts. The black arrow points to the visible death initiation stage, and the white arrow points to the well-differentiated last surviving spikelet. Scale bar 500 *µ*m. **B)** Graphs show the absolute levels of abscisic acid and sucrose in different spike parts of Morex. Plots show means ± Sd calculated from 3 to 5 biological replicates (Materials and Methods). Significance was tested using 2-way ANOVA with Tukey's multiple comparison test to evaluate the difference between apical, central, and basal positions at each stage (Supplemental Data Set S44). **C)** Venn diagram depicting the number of genes identified as apical specific in cv. Bowman and cv. Morex, as well as shared genes among both genotypes and *P*-value, showing the significance of overlap (Fisher's exact test). **D)** Expression heatmap with Euclidian distance of top 50 (based on TPM values) apically enriched overlapping genes in cv. Morex highlighted with a few genes related to growth repression, senescence, abscisic acid biosynthesis, and cell death. Heatmap represents ln(*X* + 1)-transformed mean TPM values are depicted by color code: red, high; blue, low expression. **E)** Bar plot shows the increasing apical-specific expression of the PTD candidate gene *HvGT1* (BaRT2v18chr4HG208140.2) in Morex. Plots show means ± Sd of TPM values from 3 biological replicates (Materials and Methods; Supplemental Data Set S29). **F)** Spikelet survival in the 6 major haplotype groups (minor allele frequency ≥ 0.05) among 358 6-row accessions grown in the greenhouse and field. Plot shows all individual data points with whiskers covering minimum and the maximum values; horizontal line in the box represents the median. The numerical values below each box represents the number of accessions (Supplemental Data Set S40). The letters above box plots depict significant levels determined from ordinary 1-way ANOVA with Tukey's multiple comparison test (Supplemental Data Set S45). W, Waddington scale; SEM, scanning electron microscope; cv., cultivar; FW, fresh weight; *TPS*, *TREHALOSE-6-PHOSPHATE SYNTHASE*; *ASN*, *ASPARAGINE SYNTHETASE*; *ABA4*, *ABA DEFICIENT4*; *NAC*, *(NAM [no apical meristem]*; *ATAF1/2*, *CUC [cup-shaped cotyledon])*; *DRM*, *DORMANCY-ASSOCIATED GENE/AUXIN-REPRESSED FAMILY PROTEIN*; *GT1*, *GRASSY TILLERS1*; *HIR3*, *HYPERSENSITIVE-INDUCED REACTION3*.

Six-rowed cv. Morex also showed robust and reproducible spike developmental patterns when grown in precisely controlled growth conditions (Supplemental Fig. S17A). Under similar growth conditions, Morex produced 35 to 36 rachis nodes/ridges at W5.5 (46 DAS), after which 10 to 11 nodes degenerated with the visible collapse of the inflorescence dome (visible death initiation stage) around W8.25 (62 DAS), with a spikelet survival rate of ∼72% (Fig. 8A). PI–FDA staining also showed that cell death begins from the inflorescence dome and progresses basipetally at W8.5 (66 DAS), affecting the anthers first, similar to cv. Bowman (Supplemental Fig. S17B).

Most phytohormones and metabolites showed consistent patterns similar to those seen in cv. Bowman with few differences (Fig. 8B and Supplemental Figs. S17, C and D, and S18). ABA levels peaked at W8.25 (visible death initiation) and increased ∼5-fold in apical parts at the death progression stage (W8.5), pointing to a strong association between ABA and spike PTD (Fig. 8B). Auxin, CK, sucrose, T6P, and Gln levels were limited in the apical parts of Morex spikes, in contrast to the well-differentiating central and basal parts, which was consistent with their proposed importance for spikelet growth and differentiation (Fig. 8B and Supplemental Figs. S18 and S19, A and B). High levels of Asn, branched-chain, and aromatic AAs accumulated in apical parts, similar to cv. Bowman (Supplemental Fig. S19B). However, most of these AAs increased at W6.5 in Morex, although visible death initiation was reasonably delayed (W8.25) compared to Bowman (W7.5). Nevertheless, degenerating apical parts of Morex underwent a similar metabolic reprogramming as Bowman, suggesting conserved regulation of spike PTD in both barley row types.

RNA-seq analysis also highlighted the overlap in transcriptional reprogramming behind spike PTD in 6-rowed Morex and 2-rowed Bowman (Supplemental Data Sets S28 to S35). Enriched GO terms among DETs identified distinct biological processes differentially regulated between viable (central and basal) and degenerating apical parts similar to 2-rowed Bowman (Supplemental Fig. S20, A to D, and Data Sets S30 and S31). Strikingly, most genes enriched in the apical parts of Morex significantly overlapped (623 genes; *P* < 2.2*e*^−16^) with apically enriched genes in Bowman (Fig. 8C and Supplemental Data Sets S32 to S35). The overlapping genes included transcriptional regulator genes of light signaling, senescence, cell death, defense responses, or growth repression (Supplemental Fig. S20D and Data Sets S35 and S36).

Additionally, a WGCNA identified the senescence regulator *HvS40*-like among the top 10 hub genes in the PTD-associated gene network module in which *HvGT1* is also coexpressed, similar to Bowman (Supplemental Fig. S21, A to C, and Data Sets S37 and S38). Importantly, we also identified *HvGT1* among the top 50 (based on TPM) overlapping genes highly expressed in apical parts at all 3 stages (Fig. 8, D and E). Predicted undirected targets of HvGT1 identified by our WGCNA coexpression network analysis included the senescence regulator *HvS40-like* similar to Bowman and other growth repressors (Supplemental Data Set S39).

Induced knockout of *HvGT1* improved spikelet survival and final spikelet number at heading in 2-rowed barley. Therefore, we asked whether natural variation in and around *HvGT1* associates with spikelet survival by conducting a candidate gene association study using a panel of 358 6-rowed spring barley (Kamal et al. 2021) and 300 diverse barley accessions (Jayakodi et al. 2020). These analyses resulted in significantly associated SNPs (single nucleotide polymorphisms) ∼8 kb upstream of *HvGT1* (Supplemental Fig. S22A and Data Set S40), which is close to an open chromatin region identified by a previous study (Lu et al. 2019). We predicted that this region is recognized by bZIP TFs that play roles during stress responses and plant development, suggesting that sequence variation in this region is likely to have functional consequences (Supplemental Fig. S22, A to C).

We thus conducted a haplotype analysis based on the SNPs located close to this region. We determined that haplotype group 6, which contained the alternative allele at the peak SNP, is associated with significantly higher spikelet survival compared to the other groups, based on phenotypic data collected from 358 6-rowed spring barley accessions in both greenhouse and field conditions (Fig. 8F and Supplemental Fig. S18B and Data Set S40). These findings suggest that natural *HvGT1* genomic variation may contribute to phenotypic differences for barley spikelet survival.

## Discussion

In this study, we assembled a comprehensive molecular framework of spike PTD in barley that potentially affects final grain number and yield. Observations of the MYP stage, the maximum number of spikelet/spikelet primordia, the onset of death, and the number of dying spikelet nodes were highly reproducible for both barley row types investigated here (Figs. 1, A and B, and 8A and Supplemental Fig. S17, A and B). While spikelet initiation and spike PTD are highly susceptible to environmental influences (Gol et al. 2021) (Boussora et al. 2019), spikelet initiation and spike PTD in the main-culm spike occurred as a well-orchestrated developmental program that is predictable for a given genotype under standardized growth conditions. Multi-omics analyses identified drastic molecular changes associated with apical degeneration that began at pre-death (W6.5) in both row types investigated, even though we observed no visible cell death at this stage. Spike PTD appeared to be highly conserved in both barley row types with substantially overlapping metabolic and transcriptional changes. Using tissue-specific enrichment, we identified specific TF gene families associated with barley spike PTD. We confirmed that one of these genes, *HvGT1*, played a role during the onset of PTD and growth repression of apical spikelets in barley.

### Spike PTD involves transcriptional regulators of senescence, plant defense, and light signaling

Senescence in plants is often classified as mitotic or replicative senescence (involves meristematic tissues and cell cycle/proliferative arrest) and postmitotic organ senescence (typically in organs like leaf, stem, floral petal, and roots) or the organismal level (annual crops undergo whole plant senescence for nutrient reallocation during grain filling) (Gan 2003; Woo et al. 2019). Leaf senescence, the most widely studied type of organ degeneration, involves several transcriptional regulators (Woo et al. 2019). During spike PTD, we detected the expression of several leaf senescence–related genes in the dying apical parts of the spike, including *HvS40*-*like* and NAC TF genes (Fig. 5, A and C, and Supplemental Data Sets S1, S19, and S20). Identifying *HvS40-like* as a joint top hub gene in both barley row types within the WGCNA PTD-associated gene network modules underscored its potential importance for spike PTD (Fig. 6, A and B, and Supplemental Figs. S9, A to C, and S21, A to C, and Data Sets S22 to S24 and S37 to S38). Its homolog, *HvS40*, encodes a senescence regulator domain–containing nuclear–targeted protein and is expressed during age/dark-induced leaf senescence and pathogen response (Krupinska et al. 2002).

Interestingly, many of the homologs of enriched NAC TF genes play a significant role in leaf senescence in barley and other species. For instance, *HvNAC027* (homologous to Arabidopsis *NAC-LIKE, ACTIVATED BY AP3/PI* [*NAP*] TF gene) is expressed during flag leaf senescence in barley and is highly responsive to ABA and methyl jasmonate (Christiansen et al. 2011). Nevertheless, its orthologs in wheat (*TaNAC69*) and rice (*Oryza sativa*) (*ONAC122*) play a role in disease resistance and abiotic stress tolerance (Sun et al. 2013; Xue et al. 2011). Additionally, in Arabidopsis and rice, *NAP* is involved in ABA biosynthesis for the upregulation of transcripts involved in chlorophyll degradation during leaf senescence (Liang et al. 2014; Yang et al. 2014), which might function similarly in barley spike PTD (Fig. 4, E and F). *HvNAC008/SF6*, which is highly expressed in apical tissues, was previously reported to have leaf senescence–associated expression in barley. Its closest Arabidopsis hit, *ORESARA1* (*ORE1*), is a well-characterized regulator of leaf senescence and programmed cell death (Matallana-Ramirez et al. 2013). These pieces of evidence strongly suggest that the *HvNAC*s identified here might function similarly in spike PTD. Future research on increasing yield potential should also explore the potential role of these NACs during PTD in barley and related cereals.

**Figure 9.**
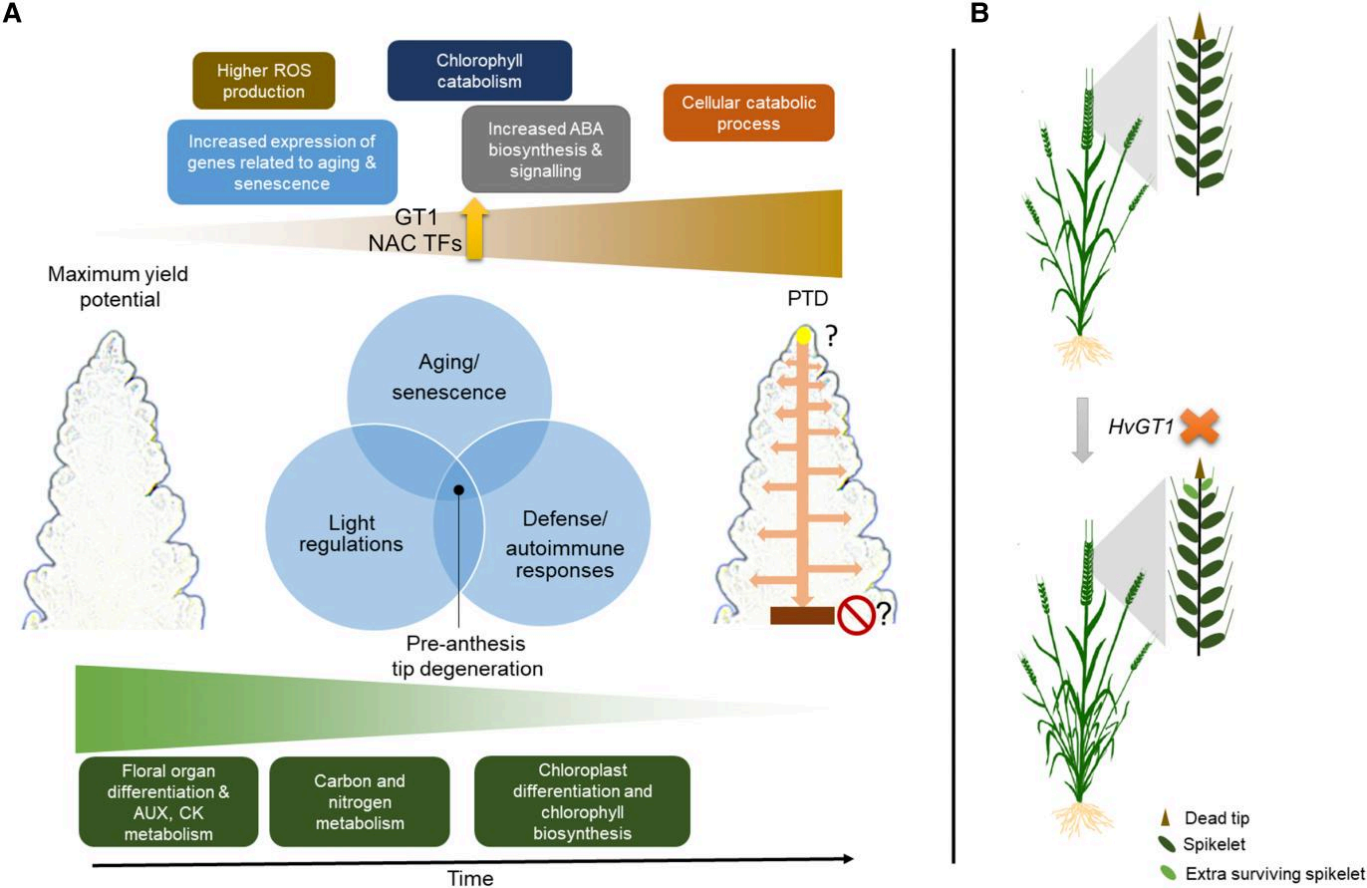
Proposed model of spike PTD in barley. **A)** Apical spikelets in the barley inflorescence undergo death, involving processes related to senescence, ABA biosynthesis, and increased ROS, chlorophyll, and protein catabolism. Increased expression of *GT1* and NAC TF genes suggest their functions as positive regulators of spike PTD. At the same time, dying apical parts exhibited reduced primary metabolism, auxin, cytokinin biosynthesis, and chloroplast differentiation, which seems important for spike growth and proper spikelet differentiation. Moreover, there seems to be an overlapping function of aging/senescence, light, and autoimmune response regulators on PTD. However, the actual signal(s) that might originate from the IM tip (solid yellow circle with arrows pointed downwards and laterally into the spikelets) and the signal(s) that defines the death zone (solid brown box with stop sign) remains elusive. **B)***HvGT1*, identified as one of the putative modulators of spike PTD, acts as a growth repressor that negatively affects the development of apical spikelets. Its loss-of-function (orange “X” symbol) promotes apical growth with delayed degeneration and enhanced differentiation of apical spikelets, thereby increasing the final spikelet number. ABA, abscisic acid; ROS, reactive oxygen species, *GT1*, *GRASSY TILLERS1*; NAC, (NAM [no apical meristem], ATAF1/2, CUC [cup-shaped cotyledon]); IM, Inflorescence meristem.

The effects of light/circadian regulators on environmental adaptation and flowering and their relevance to spike development have been documented in barley (Boden et al. 2014; Bi et al. 2019). Molecular mechanisms connecting leaf senescence and light signaling have also emerged recently (Kim and Hong 2019; Kim et al. 2018; Lee et al. 2021). Similarly, this study found regulators involved in light senescence crosstalk, such as *ETHYLENE INSENSITIVE3* (*HvEIN3*), a homolog of Arabidopsis *EIN3*, which regulates age-dependent leaf senescence (Li et al. 2013), indicating the possibility of such a control mechanism for spike PTD in barley (Fig. 5C and Supplemental Data S20). Very recent research also suggested the presence of a vascular-specific clock within the developing barley spike that might regulate spikelet growth and spike PTD (Huang et al. 2023). We also observed the enrichment of plant defense response genes, including *HvDRM/ARP* and *HvHIR3* (*HYPERSENSITIVE INDUCED REACTION3*) (Fig. 8D and Supplemental Data Sets S11 and S17). Their Arabidopsis homologs, *AtDRM1/2/ARP*, function in axillary bud dormancy, stress responses, and negative regulation of local and systemic acquired resistance (Rae et al. 2013; Rae et al. 2014; Roy et al. 2020), whereas *HvHIR3* functions as a positive regulator of hypersensitive response (HR)-like cell death (Rostoks et al. 2003). An independent study found a 35-fold increase in *HvHIR3* expression in the leaves of barley disease lesion mimic mutants, which show spontaneous HR-like cell death (Rostoks et al. 2003). Recently, a mutant in a proton pump *ATPase* gene exhibiting lesion mimic leaf and panicle abortion was characterized in rice (Hu et al. 2022). Although rice panicle abortion is morphologically distinct from barley spike PTD, characterizing barley lesion mimic mutant resources might help us better understand this cell death phenomenon. In fact, stem cell–derived immune signaling in the shoot apical meristem was previously hypothesized (Wu et al. 2020a), and loss of key immune signaling regulators may cause autoimmunity leading to death (Wu et al. 2020b). Thus, we postulate that PTD in barley might also occur due to the inability of the inflorescence tip to maintain proper immune signaling. This evidence suggests that spike PTD may be collectively regulated by senescence, autoimmunity/defense responses, and light signaling (Fig. 9A).

### Barley GT1, a modulator of spike PTD

Gene editing to knock out one of the putative candidates, *HvGT1*, enhanced apical spikelet development and differentiation, substantially delayed degeneration, and increased the final spikelet number in 2-rowed barley (Fig. 7). This result supports the evolutionarily conserved function of HD-ZIP class I family members in growth repression, as observed in grasses and eudicots (Komatsuda et al. 2007; Whipple et al. 2011; González-Grandío et al. 2017; Sakuma et al. 2017; Gallagher et al. 2023). The maize ortholog *GT1* is an essential domestication gene that suppresses carpel development in tassel florets and elongation of lateral ear branches and promotes axillary bud dormancy putatively regulated by the other domestication gene *TB1*, encoding a TCP TF (Dong et al. 2019; Klein et al. 2022; Whipple et al. 2011). This domestication module in maize induces tiller bud dormancy by recruiting inhibitory phytohormones, such as ABA and jasmonate, thereby altering carbohydrate homeostasis (Dong et al. 2019).

The findings in maize suggest that *HvGT1* might also function similarly in barley by modulating ABA and sugar signaling to suppress apical spikelet growth. However, barley *gt1* mutants did not show any carpel derepression phenotype in the spikelets of the non-dying region, in contrast to that seen in maize tassel florets (Klein et al. 2022). Notably, in barley, carpel suppression in lateral spikelets is controlled by the row-type *VRS* genes (Koppolu and Schnurbusch 2019). The *TB1* ortholog in barley, *VRS5*/*INT-C*/*HvTB1*, functions in lateral spikelet fertility/sterility, and its loss of function causes increased tillering in the early phase of plant development and partial fertility in the lateral spikelets of 2-rowed barley (Ramsay et al. 2011; Zwirek et al. 2019). Moreover, the expression domains of *HvTB1* and *HvGT1* were different in the barley IM, suggesting that they might function independently during barley spike growth, in contrast to maize, aside from their conserved tillering function. Thus, GT1 may have gained a novel function in barley during the onset of spike PTD and suppression of apical spikelet growth, most likely involving ABA pathway genes, which will require further downstream analysis of *gt1* mutants. Indeed, identifying the downstream targets of HvGT1 could help us understand the observed phenotype in the mutant. These results identify GT1 as a modulator of spike PTD in barley independent of the flowering time pathway and further demonstrate the possibility of manipulating PTD to increase yield potential in cereals (Fig. 9B).

However, the extra-surviving apical spikelets in *gt1* mutants fail to produce regular grains in most of the examined spikes. This could be attributed to various reasons such as growth conditions, genetic background, and border effect. On the other hand, the 2-rowed cultivar “Golden Promise” (used for transformation) often showed irregular ectopic spike branches in the basal nodes under a glasshouse light regime when grown in pots independent of *gt1* mutations as reported in previous studies (Brown and Bregitzer 2011). However, such ectopic spike branches were absent in the plants grown in soil under natural sunlight. Hence, it is still necessary to test the grain yield advantage of the *gt1* mutation by growing more plants and testing the mutant effects in other genetic backgrounds. Further, the loss of HvGT1 function achieved by targeted mutagenesis only delayed, but did not abolish, PTD, thereby facilitating more differentiation. Still, upstream regulators of spike PTD need to be identified, which requires further study.

### Later events of spike PTD are predominantly associated with higher ABA levels

Phytohormones serve as local or long-distance signals in several developmental and stress-related processes (Lacombe and Achard 2016; Mazzoni-Putman et al. 2021; Blazquez et al. 2020). Stress-responsive plant hormones, including ABA, jasmonic acid (JA), salicylic acid (SA), and ethylene, were previously characterized for their role in plant cell death and senescence (Woo et al. 2019). Our phytohormonal profiling revealed high levels of ABA in the apical part, which coincided with the onset and basipetal progression of tip degeneration. ABA was recently suggested to be involved in spikelet primordia abortion in 6-rowed barley under salt stress (Boussora et al. 2019). Whereas, the present study demonstrates the involvement of ABA during spike PTD of both row types, even under standardized growth conditions (Methods; Figs. 2D and 8B and Supplemental Figs. S2A and S18).

ABA levels correlate with the increased transcript levels of the key ABA biosynthesis gene *HvNCED1* (Leymarie et al. 2008; Seiler et al. 2014). Additionally, the alternative ABA biosynthesis route was also active, as indicated by the upregulation of *ABA4*. The homologous encoded proteins in Arabidopsis and rice are plastid localized and involved in stress-induced ABA accumulation (North et al. 2007), photoprotection (Dall’Osto et al. 2007), and root growth inhibition (Ma et al. 2014). ABA also represses the transcription of chloroplast genes (Yamburenko et al. 2015), supports reduced chlorophyll levels, and causes poor chloroplast differentiation toward the apex. Importantly, ABA also inhibits specific developmental PCD, thereby regulating the proper onset and slow progression of cell death (Van Hautegem et al. 2015; Young and Gallie 2000).

Similarly, barley spike PTD is not a rapid cell death event; instead, it undergoes a gradual progression that may last for weeks, depending on the genotype. Notably, ABA levels peak in the apical part during or after the collapse of the IM dome (Fig. 2D and Supplemental Fig. S2). In such cases, ABA might regulate the timing/onset and progression of PTD either antagonistically or mutually with other possible regulators; however, this question will await studies until the end of tissue death. These observations provide a hypothesis for future studies on testing the function of ABA biosynthesis/signaling on the regulation of barley spike PTD.

Although we did not measure the absolute quantities of SA, JA, or ethylene, we observed an enrichment of genes involved in ethylene biosynthesis/signaling and JA and SA responses (Supplemental Data Sets S6, S7, S11, and S15), suggesting a role for these phytohormones during barley spike PTD. The ethylene biosynthesis gene *1-aminocyclopropane-1-carboxylate oxidase2* (*ACO2*) controls floret fertility and kernel number in maize ears (Ning et al. 2021). Interestingly, our study also identified its barley ortholog *HvACO* as a highly expressed gene in degenerating apical parts (Fig. 4D). Indeed, apical spike parts had reduced levels of growth-promoting phytohormones, such as IAA, GA precursors, and CK ribosides (cZR and iPR), which might limit further growth and differentiation (Fig. 2D and Supplemental Figs. S2 and S18). A recent study in barley showed the significance of local auxin biosynthesis for the maturation of pollen grains by enhancing the expression of central metabolism genes, thereby promoting growth and differentiation (Amanda et al. 2022). Overall, differential gradients of growth-promoting and repressing phytohormonal regulation in dying and viable spike parts may establish the final spikelet number in barley.

### Apical spikelets undergo metabolic reprogramming typical of organ senescence in plants

Maintaining energy homeostasis is a key survival factor in all living organisms. During organ senescence/cell death, such as in leaves, drastic structural and biochemical changes take place sequentially, involving a metabolic transition from anabolism to catabolism and hydrolysis of macromolecules (Woo et al. 2019). Based on whole-spike measurements (Ghiglione et al. 2008), sugar starvation has been postulated to play a role in the death of more distal florets in wheat. We found that dying apical parts were low in carbohydrates, particularly Suc and T6P, whereas viable central and basal parts contained high levels of these metabolites (Figs. 3 and 8B and Supplemental Fig. S19). Such metabolic gradients strongly identify carbohydrate homeostasis as one of the main factors during spike PTD, thereby confirming the significant role of sugars in plant growth and development (Eveland and Jackson 2012). While Suc and T6P are important factors during plant development and signaling, respectively, high Fru and Tre levels were previously observed during leaf senescence (Watanabe et al. 2013). Our transcriptome analysis also showed a strong enrichment of carbon metabolism and photosynthesis-related genes being highly expressed in the central and basal parts of the spike. Here, substantial chloroplast differentiation and greening can be seen, which might support spikelet growth via a proper supply of photoassimilates.

AAs, which also serve as precursors for phytohormones, nucleotides, and several secondary metabolites, are involved in plant development and stress responses in addition to building proteins (Huang and Jander 2017; Lynch and Dudareva 2020; Yadav et al. 2019). The distribution of AAs in degenerating apical parts resembles that of stressed or senescent cells, which show increased levels of protein degradation due to proteolysis, which might explain the increase of AA contents (Hildebrandt et al. 2015). High levels of AAAs, BCAAs, Lys, and Pro in degenerating apical tissues may serve as precursors for stress-related secondary metabolites and alternate substrates under energy-limited conditions, as observed in Arabidopsis (Peng et al. 2015; Watanabe et al. 2013) and wheat (Heyneke et al. 2019; Zhu et al. 2022). Here, the apical part, limited in carbon supply, may use the degraded AAs as alternative substrates for energy production (Law et al. 2018). Notably, transcriptome analysis also revealed significant enrichment for genes related to AA catabolism in the apical parts.

The most abundant AA accumulating in the apical part of both genotypes was Asn, which coincided with the high expression of *ASPARAGINE SYNTHETASE* genes (*HvASN1* and *HvASN5*) in degenerating tissues (Supplemental Data Sets S11 and S33). An independent study in barley showed increased expression of these genes in developmental and dark-induced senescent leaves, probably serving a role in nitrogen (N) remobilization (Avila-Ospina et al. 2015). The Asn distribution and associated gene expression pattern favor a role for this AA as a major form of N transport and supply during spike PTD, whereby N might become available in the dying parts. During leaf senescence, mitochondria and nuclei, required for energy production and gene expression, respectively, remained intact to effectively mobilize cellular contents until senescence was completed. Besides changes in AA levels, the expression of several transporter genes and those involved in autophagy and vesicle trafficking increased in the apical part, suggesting that newly available nutrients in the degenerating apical region become reallocated to the viable region.

In summary, spike PTD in barley is a complex quantitative trait that likely involves multiple regulatory layers, including molecular processes typical for organ senescence/cell death (Woo et al. 2019). Despite the discovery of several molecular regulators in the present study, fundamental questions remain about the signal(s) determining the precise timing, initiation, and marking of the affected spikelet primordia (i.e. extent of death zone) (Fig. 9A). Nevertheless, the candidates identified here may serve as an excellent resource for exploiting their natural allelic variants, while their functional validation and manipulation via gene editing may lead to an increase in grain yield potential in barley and other related cereals.

## Materials and methods

### Plant materials and growth conditions

Two-rowed barley (*H. vulgare*) cv. Bowman (hereafter Bowman) and 6-rowed barley cv. Morex (hereafter Morex) were grown in 9-cm square pots (1 plant per pot) in a climate chamber (IPK, Gatersleben) at 12 °C/8 °C and 12-h/12-h light–dark photoperiod with a light intensity of 300 *µ*E (fitted with metal halide and light-emitting diode [LED] lamps) at 80% humidity as described previously (Thiel et al. 2021). Two-rowed cv. Golden Promise T_2_*Hvgt1* knockout lines were grown for phenotyping spike and plant traits in a glasshouse containing natural loamy soil under sunlight and porous metal netting on the sides to enable air circulation (the growth period was between April 2022 and August 2022 at IPK, Gatersleben 51°49′23.1″N, 11°16′ 41.7″E). Lines were sown in jiffy pots in a controlled environment and 10 d later transplanted in soil with ∼10 cm distance between plants within a 1-m row. For the greenhouse experiment, plants were grown in 9 cm square pots under long-day conditions of 16-h/8-h light–dark regime at 17 °C/14 °C. For all the phenotyping and molecular/biochemical analyses, the immature spike meristems were sampled only from the main culm of the plants between 6 and 10 h after initiating the light phase. Optical images of the spike meristems were captured using a Zeiss stereomicroscope.

### Phytohormone measurements in immature spike meristems

For phytohormone measurements of Bowman, apical, central, and basal spike parts were collected in stages W5.0, W6.5, W7.5, and W8.0, with the apical part covering all the dying spikelet nodes/ridges, central and basal, with 11 spikelet nodes each leaving 3 underdeveloped spikelets at the base. In the case of Morex, stages W5.5, W6.5, W8.25, and W8.5 were collected with the apical part covering all dying spikelet nodes/ridges and central and basal parts with 11 spikelet nodes, each leaving 2 underdeveloped spikelets at the base. Four biological replicates per sample were used. For each biological replication, 15 (W5.0, W5.5), 10 (W6.5), and 5 (W7.5, W8.0, W8.25, and W8.5) spike meristems were dissected and collected as apical, central, and basal parts in separate vials, frozen in liquid nitrogen, and stored at −80 °C. Two stainless steel beads were added to each sample tube and placed in precooled (−80 °C) TissueLyser II (QIAGEN, USA) racks and homogenized at 30 Hz for 1.5 min. Fresh weights were measured using a precision balance. Samples of 3.5 to 30 mg (fresh weight) were weighed into 2 mL safe lock tubes (Eppendorf AG, Germany) and kept at −80 °C until use. Before extraction, 2 3-mm ceria-stabilized zirconium oxide beads were placed into each tube. The samples were extracted and purified as described previously with minor modifications (Simura et al. 2018). For UHPLC-ESI-MS-MS analysis, samples were dissolved in 50 *µ*L of 30% ACN (*v*/*v*) and transferred to insert-equipped vials. The absolute quantification of targeted phytohormones was performed as described (Eggert and von Wiren 2017). Ten microliters of purified extracts were injected into an Acquity Ultra Performance LC system coupled with a Xevo TQ mass spectrometer (Waters, USA). All targeted phytohormones but gibberellins were separated on an Acquity UPLC BEH C18 1.7 *μ*m, 2.1 × 100 mm column coupled to a VanGuard pre-column BEH C18 1.7 *μ*m, 2.1 × 5 mm. A 10-point external calibration curve was used for quantification. MassLynx software (version 4.1; Waters) was used to control the instrument and data acquisition. MS data were processed with TargetLynx V4.1 SCN 904. Gibberellin baseline separation was achieved on a reversed-phase Acquity. The UHPLC system was coupled to a Q Exactive Plus Mass Spectrometer (San Jose, CA, USA) equipped with a HESI source operating in negative ion mode. MS data were acquired and processed by Trace Finder Software (v. 4.1, Thermo Scientific, San Jose, CA, USA). The peak area on the extracted ion chromatogram (XIC) of the deprotonated molecule ion [M-H] was measured to generate the calibration curve. A least-square linear regression was used to fit the linearity curve best. The identification of compounds found in extracts was based on comparing their retention time, high-resolution *m/z* spectrum, and isotope pattern with standards. Additionally, generated MS2 spectra were searched in a custom spectral library to confirm compound identification. For the greenhouse experiment, Bowman spikes were collected at stages W5.0, W7.0, and W8.0 and performed the hormone profiling similarly as described above.

### Primary metabolite profiling in immature spike meristems

To extract polar metabolites, spike samples similar to hormone measurement were collected in separate vials, frozen immediately in liquid nitrogen, and stored at −80 °C. Five biological replicates per sample were used for this analysis. For each biological replication, 15 (W5.0, W5.5), 10 (W6.5), and 5 (W7.5, W8.0, W8.25, and W8.5) spike meristems were dissected from Bowman and Morex and collected as apical, central, and basal parts in separate vials as similar to phytohormone measurements, frozen in liquid nitrogen, and stored at −80 °C. Two stainless steel beads were added to each sample tube and placed in precooled (−80 °C) TissueLyser II (QIAGEN, United States) racks and homogenized at a frequency of 30 Hz for 1.5 min. Samples of 3.5 to 40 mg (fresh weight) were weighed into 2 mL safe lock tubes (Eppendorf AG, Germany) and kept at −80 °C until use. One milliliter of the extraction buffer containing chloroform:methanol in a 1:1 (*v*/*v*) ratio was added. Samples were mixed thoroughly and placed on a vortex at 4 °C while shaking carefully for 20 min. Afterwards, 300 *µ*L LC-MS grade water was added to each sample and mixed. After centrifugation at 18,400 × *g* at 4 °C for 10 min, the polar upper phase was transferred to a new reaction tube (Eppendorf AG, Germany) and used directly for the measurement or stored at −80 °C for further analysis. Soluble sugars (sucrose, glucose, and fructose) were measured enzymatically as described previously (Ahkami et al. 2009). For the targeted analysis of AAs, 100 *µ*L of vacuum-dried polar phase from each sample extract was redissolved in 10 *µ*L of water. The AAs were derivatized with AQC (6-aminoquinolyl-N-succinimidyl carbamate) using the Bioanalytics Gatersleben Amino-Acid kit (Germany). Briefly, 10-*µ*L samples were added with 70 *µ*L of the derivatization buffer and 20 *µ*L of the AQC reagent, followed by a 10-min incubation at 55 °C, and cooled down for analysis. One microliter of the derivatized AAs was analyzed through RP-UPLC-PDA-FLR (RP-UPLC-PhotoDiode Array-Fluorescence detection) using an Acquity UPLC system (Waters, Germany). AAs were separated in an AccQTag Ultra (1.7 *µ*m, 2.1 × 100 mm; Waters, Germany) column coupled to an Acquity In-Line filter (Waters, Germany) at 60 °C and 0.7 ml.min^−1^. The separation gradient was as follows: starting isocratic hold with 0.1% *v*/*v* eluent B for 0.5 min, from 0.1% to 2% *v*/*v* B from 0.5 to 5.7 min, from 2% to 9% *v*/*v* B from 5.7 to 7.5 min, from 9% to 9.7% *v*/*v* B from 7.5 to 8.7 min, from 9.7% to 13% *v*/*v* B from 8.7 to 9.2 min, from 13% to 14% *v*/*v* B from 9.2 to 12 min, and from 14% to 60% *v*/*v* B from 12 to 12.2 min and an isocratic hold from 12.2 to 12.7 min to clean the column; after each run, the column was equilibrated to the starting conditions (0.1% *v*/*v* B). Eluent A was the AccQTag Ultra Eluent A concentrate diluted 1:10 (*v/v*) in water, and eluent B was the AccQTag Eluent B concentrate (Waters, Germany). The derivatized amino acids were analyzed by fluorescence (excitation/emission 266/473 nm, PMT gain 1.0, data rate 10 points.s^−1^) and PDA (260 nm, 4.8 nm resolution, sampling rate 10 points.s^−1^) detection. For the targeted quantitation of each amino acid, we used calibration curves of commercial standards in the column range of 0.1 to 75 pmol. Data processing and analysis were done with the Empower 3 software (Waters, Germany).

Sugar phosphates and organic acids were measured as described previously (Ghaffari et al., 2016). Briefly, the separation and detection of metabolites were carried out using an ion chromatography system with a conductivity detector (Dionex, Thermofisher Germany) connected to a triple quadrupole mass spectrometer QQQ6490 (Agilent Technologies, Waldbronn Germany). ESI-MS/MS analysis was conducted in the negative ionization mode, and following parameters were employed: desolvation temperature of 250 °C, nitrogen gas of 720 l per hour with a heater temperature of 250 °C, capillary voltage of 3.5 KV and different dwell times between 40 and 200 sec. Collision energy (CE) differed among the compounds and was in the range between 6 and 50 for different masses. Deprotonated ions [M-H]^-^ were monitored with a span of 1 amu. Multiple reactions monitoring (MRM) was performed to identify individual compounds accurately. This allows for minimizing parallel monitoring and enhancing sensitivity. Quantification was performed with authentic standards (Sigma-Aldrich, Germany) at different concentrations. The relative abundance of additional polar metabolites was analyzed through untargeted LC-MS-based profiling. Polar extracts were added 4:1 (*v/v*) with 0.5% *v/v* formic acid. Samples were analyzed by RP-UPLC-ESI-UHR-QTOF-MS (Reversed Phase Ultra Performance LC-Electrospray Ionization-Ultra-High-Resolution-Quadrupole Time Of Flight MS) using an Acquity UPLC system (Waters, Germany) coupled to a maXis Impact ESI-QTOF MS (Bruker Daltonik GmbH, Germany) with an injection volume of 5 *µ*l. The separation was done in an Acquity UPLC HSS T3 (1.8 *µ*m, 2.1 x 100 mm; Waters, Germany) column coupled to an Acquity UPLC HSS T3 VanGuard (1.8 *µ*m, 2.1 x 5 mm; Waters, Germany) pre-column, at 30 °C and 0.4 ml.min^−1^, using the following gradient (eluent A, 0.1% *v/v* formic acid in water; B, 0.1% *v/v* formic acid in acetonitrile): starting isocratic hold with 0.1% *v/v* B for 1 min, from 0.1 to 5% *v/v* B from 1 to 3 min, from 5 to 12.6% *v/v* B from 3 to 6 min, isocratic hold from 6 to 7 min, from 12.6 to 50% *v/v* B from 7 to 10 min, from 50 to 99% *v/v* B from 10 to 10.5 min, and an isocratic hold from 10.5 to 12.5 min to clean the column; after each run, the column was equilibrated to the starting conditions (0.1% *v/v* B, 99.9% *v/v* A). MS profiling for small molecules (50-1000 m/z) was done in MS1 using a positive ionization mode as described (Garibay-Hernández et al., 2021). MS/MS was performed in selected samples using auto MS/MS mode for annotation purposes (Garibay-Hernández et al., 2021). Data processing and quantitative analyses were performed with MetaboScape 5.0 (Bruker Daltonik GmbH, Germany) using an intensity threshold of 1500 counts and a minimum peak length of 7 spectra. The Compass DataAnalysis 4.4 SR1 package (Bruker Daltonik GmbH, Germany) was used for manual data inspection. Amino acids and trehalose were confirmed with a commercial standard by retention time (0.75 min), exact mass (error < 3 ppm), isotopic pattern, and MS/MS fragmentation. The relative quantitation of trehalose was based on the most intense ion, [M+H-H2O]^+^ m/z 325.1138. The MS/MS fragmentation of this precursor mass resulted in the characteristic ions [M+H-C6H10O5]^+^ m/z 163.0643 and [M+H-C6H12O6]^+^ m/z 145.0495. For all the polar metabolites described above, values were normalized to the fresh weight of starting extracted material.

### MALDI-TOF MS imaging

Bowman spike samples (W5.0, W6.5, W7.5) were collected and frozen immediately in liquid nitrogen and stored at –80 °C. two samples at each stage were collected and considered as biological replicates. Samples were then embedded in 10% w/v gelatin (Sigma-Aldrich, Germany) medium and frozen. The samples were then transferred into a cryostat (Thermo scientific Cryostar NX70, Germany) at −20 °C and equilibrated for 1 hour. Following median longitudinal sections of 10 μm thickness were made and thaw mounted onto indium tin oxide (ITO, Bruker Daltonics, Germany) coated glass slides (75 mm × 25 mm). For drying, slides were placed into a vacuum desiccator for ∼30 min before optical imaging. 2,5-dihydroxybenzoic acid (DHB) matrix was sublimated over the tissue surface with a lab-made sublimator (Shanmugaraj et al., 2023). MALDI imaging was performed using an Ultraflextreme MALDI-TOF/TOF MS (Bruker Daltonics, Germany) equipped with a smartbeam II laser in a positive ionization mode at a repetition rate of 1000 Hz as described previously with few modifications (Peukert et al., 2014). The data was acquired in the small mass range between the mass window of m/z 80-1000 Da and the sample rate at 1 Gs s^−1^. The spatial resolution was set to 15 *µ*m, and 600 shots per raster spot were acquired in reflectron mode. The instrument was calibrated with 0.25 *µ*L polyethylene glycol mixture (1:1 PEG 200 and 600, diluted 1:300 in 30% (*v/v*) acetonitrile and 0.1% (w/v) trifluoroacetic acid) before each experiment. The laser power was adjusted to exhaust the analyte signals, and the images were acquired in random sampling mode. The unprocessed data were loaded into flexImaging v4.1 and SCiLS lab 2021c pro software (Bruker Daltonics, Germany). Data processing in the SCiLS lab was done according to the instruction manual. Reduced spectra (.mis file) were loaded into the SCiLS lab and proceeded with the default automatic mass axis setting (constant: equidistant). A convolution algorithm was used for baseline removal with a peak width value of 20. MALDI ion images were processed using total ion count (TIC) normalization and visualized.

### FDA/PI staining

For the live-death assay, spike meristems were incubated in 2-ml Eppendorf tubes containing water supplemented with 10 *µ*g/ml FDA (stock 2.5 mg/mL in acetone) and 2 *µ*g/ml PI (stock 1 mg/ml in distilled water) and incubated in the dark for 5 min on a lab shaker at 100 rpm. Stained meristems were mounted onto the chambered coverglass (Thermo Scientific, USA). FDA and PI were imaged simultaneously on a LSM780 laser scanning microscope (Carl Zeiss, Germany) as previously described (Feng et al., 2022). Briefly, employing a 10x (N.A. 0.45) objective, zoom 1.0 and image size 1024 x 1024 pixels, FDA was excited with a 488 nm laser line and emission recorded using a 500-540 nm bandpass while PI was excited with a 561 nm laser line and emission recorded using a 580-640 nm bandpass. Adjusting gain settings to avoid oversampling, Z-stacks were recorded and presented as maximum intensity projections.

### ROS staining

ROS production was observed using fluorescent dye 2',7'-dichlorodihydrofluorescein diacetate (H_2_DCFDA). Bowman spike samples at stages W5.0, W6.5, and W7.5 were incubated for 30 min with 20 *µ*M H_2_DCFDA in 20 *µ*M HEPES-NaOH buffer (pH 7.4) as well described previously (Feng et al., 2020). Fluorescence was examined using a laser scanning confocal microscope (LSM780, Zeiss) with a 10x (N.A. 0.45) objective, employing a 488 nm laser line for excitation in combination with a 490-540 nm bandpass for emission. Using zoom 1.0, image size 1024 x 1024 pixels and adjusted gain settings to avoid oversampling, Z-stacks were recorded and presented as maximum intensity projections.

### Scanning electron microscopy and TEM

Scanning electron microscopy (SEM) and TEM observations were done as described previously (Daghma et al. 2011; Poursarebani et al. 2020). For SEM, Bowman spikes were fixed in 50 mm cacodylate buffer (pH 7.2) containing 2% *v*/*v* glutaraldehyde and 2% *v*/*v* formaldehyde at 4 °C. Samples were washed and dehydrated in an ascending ethanol series and critical point dried in a Bal-Tec critical point dryer (https://www.leica-microsystems.com). Dried spike specimens were gold coated in an Edwards S150B sputter coater (http://www.edwardsvacuum.com) and examined in a Zeiss Gemini30 scanning electron microscope (https://www.zeiss.de) at 10 kV acceleration voltage. For TEM, spike samples were embedded in Spurr's resin. After ultrathin (70 nm) sectioning and staining, samples were analyzed in a Tecnai Sphere G2 TEM (FEI, Netherlands) at 120 kV.

### Total RNA extraction, RNA-seq, and gene expression analysis

Total RNA was extracted from the apical, central, and basal spike parts of the stages W5.0, W6.5, and W7.5 (Bowman) and from W5.5, W6.5, and W8.25 (Morex) using TRIzol (Invitrogen). Three biological replicates per tissue type were used for this analysis. For each biological replication, 5 spike meristems were dissected from Bowman and Morex and partitioned similar to phytohormone measurements as apical, central, and basal parts in separate vials. To remove genomic DNA contamination, RNA was treated using RNase-free DNase (TURBO, Invitrogen). RNA integrity and amounts were checked via Agilent 2100 Bioanalyzer (Agilent Technologies) and Qubit (Invitrogen). RNA samples were sent to Novogene (United Kingdom) for strand-specific RNA libraries (PE150) preparation and high-throughput sequencing using the Novaseq 6000 platform yielding about 50 million reads per sample. RNA-seq quantification was done with Kallisto using the barley reference transcriptome BARTv2.18 (Bray et al. 2016; Coulter et al. 2022). The quant files were then processed in R (3.4.3). For each transcript, a total number of reads mapped on all the tissue was calculated, and only transcripts with a total count greater than 100 (Morex) and 150 (Bowman) were selected for differential expression analysis. The count threshold was determined based on voom transformation function from the limma package (Law et al. 2014), and the raw read counts were normalized to TPM expression levels. DESeq2 was used to identify DETs among 15 pairwise combinations according to log2 FC ≥ 1 and Benjamini–Hochberg FDR–adjusted *P* values (<0.05) (Love et al. 2014; Benjamini and Hochberg 1995). In parallel, the ROKU method, which uses Shannon entropy to rank genes according to their overall tissue specificity, was used to identify tissue-specific transcripts. In brief, the TPM values from replicates of tissues or stages were used to calculate the mean TPM value for each transcript, and log2 of mean TPM values were used to identify tissue-specific transcript. Gene expression heatmaps were generated using the ClustVis online tool (Metsalu and Vilo 2015). Barley reference transcriptome is publicly available at https://ics.hutton.ac.uk/barleyrtd/bart_v2_18.html. Gene IDs of barley MorexV3 genome reference (Mascher et al. 2021) were also obtained by the reciprocal blast against BARTv2.18 transcriptome reference.

### RT-qPCR

First-strand cDNA was synthesized from total RNA with SuperScript III Reverse Transcriptase Kit (Invitrogen, 18080-051). qPCR was performed using power SYBR Green PCR Master Mix (Thermo Fisher, A46112) on an ABI prism 7900HT sequence detection system (Applied Biosystems). The relative gene expression levels were determined by the 2^−△CT^ method using *HvActin* for normalization (Livak and Schmittgen 2001). Data were interpreted from 3 technical replicates for each of the 3 biological replicates. Primers used were listed in Supplemental Table S2.

### Co-expression network analysis

TPM data of all expressed transcripts from 27 samples each from Bowman and Morex were used for the construction of the coexpression network, module detection, and identification of hub genes from the modules. The data processing and coexpression analysis were carried out as described previously (Thiel et al. 2021). The expression profile of each module was summarized by module eigengene defined as its first principal component, and modules with highly correlated (Pearson's correlation > 0.75) eigengenes were merged. In this way, ten modules in Bowman and thirteen modules in Morex were identified. Furthermore, modules with expression patterns similar to genes of interest were selected to identify hub genes. For hub gene identification, the “degree” and “betweeness centrality” statistics were calculated separately for all the transcripts from each module. These values were used to rank the genes, and the top 10 genes from each module were considered as hub genes. The networks were visualized using Cytoscape 3.7.1 (Shannon et al. 2003).

### cis-motif enrichment analysis

For cis-motif identification and enrichment analysis, the first 1.5-kb upstream promoter sequences for all the genes were extracted from MorexV3. The “findMotif.pl” HOMER suite (Heinz et al. 2010) program was used for the promoter sequences of apical, central, and basal enriched genes identified in ROKU analysis. Identified motifs were searched against the 1.5 kb promoter sequences of the remaining genes for enrichment, and binomial distribution was used to calculate *P* values.

### GO enrichment analysis

Arabidopsis (*A. thaliana*) homologs were used for GO term enrichment analysis. The protein sequences of barley annotated genes (BARTv2.18) were used to query the Arabidopsis protein data set in TAIR10. Corresponding genes with the highest hit of BLASTP (*e*-value < 1*e*^−5^) in Arabidopsis were used for GO enrichment analysis. GO term enrichment was performed for the DETs from pairwise comparisons and tissue-specific DETs from ROKU analysis using their corresponding TAIR IDs with Metascape (http://metascape.org) following default parameters with a *P*-value cutoff of 0.05 (Zhou et al. 2019).

### Phylogenetic analysis

Protein sequences of GT1 and GNI1 orthologs from barley, wheat (*T. aestivum*), and maize (*Z. mays*) were used as queries to BlastP against maize (source: Ensembl), wheat (source: Ensembl), and barley (source: Morex v3 in IPK) protein databases with an *e*-value cutoff of 1*e*^−10^. This resulted in 137 nonredundant hits. Multiple sequence alignment was done using MUSCLE (REF: PMID: 15318951). Gaps present in 80% (or more) of the aligned sequences were removed using trimal (-gt 0.2) (REF: PMID: 19505945), as these gaps are considered to lack phylogenetic signals. Phylogenic tree was constructed with a neighbor joining method in MEGA using the JTT model, and 1,000 bootstrapped replicates. Corresponding gene IDs of the protein sequences used for the phylogenetic analysis were provided in the Supplemental Data Set S25. Alignment files and sequences are openly available in the Dryad Digital Repository at https://doi.org/doi:10.5061/dryad.j0zpc86k9.

### Subcellular localization

For subcellular localization, the full-length sequence of *HvGT1* (NIASHv2033D08, GenBank accession no. AK365295) (Matsumoto et al. 2011) was amplified from a full-length cDNA clone obtained from NIAS, Japan (http://barleyflc.dna.affrc.go.jp), and directionally cloned into the pENTR/D-TOPO entry vector (Thermo Fisher Inc.). After sequence verification, entry vectors were N- and C-terminally fused in frame to yellow fluorescent protein (YFP) gene in pIPKTA48 and pIPKTA49 vectors (https://figshare.com/articles/dataset/Sequence_information_of_pIPKTA48_and_pIPKTA49/6652415/1), respectively, using Gateway cloning technology (Thermo Fisher Scientific, New York, NY, USA). Resulting YFP-fusion constructs were transiently expressed in 7-d-old leaf segments of barley cv. Golden Promise by particle bombardment and examined after 24 h of incubation using an LSM 780 confocal microscope (Zeiss). YFP fluorescence was detected following their excitation with a 514 nm laser line and 517- to 562-nm emission bandpass and chlorophyll autofluorescence with a 633 nm laser line.

### Generation of *gt1* knockout barley by targeted mutagenesis

A suitable Cas9 target motif was selected in the first exon of *GT1* (Fig. 8A) using the genomic sequence of barley cv. “Golden Promise” (Schreiber et al. 2020) and the DESKGEN (Doench et al. 2014) and WU-CRISPR (Wong et al. 2015) platforms followed by modeling and validating the gRNA's secondary structure using the RNAfold tool (Gruber et al. 2008) according to criteria outlined previously (Koeppel et al. 2019). The GT1-specific 5′-end of the gRNA–coding transgene was synthesized as single DNA strands with overhangs (forward: 5′-TGGCGAAGCTGTCCGGGAAGACGA-3′ and reverse: 5′-AAACTCGTCTTCCCGGACAGCTTC-3′) that then were annealed and cloned into the intermediate vector pSH121 (GenBank ID: MW145140.1) (Hoffie et al. 2021) using *BsaI* restriction sites. Subsequently, the plasmid fragment containing the expression cassettes of *GT1*-specific gRNA and cas9 was subcloned into the binary vector p6i-d35S-TE9 (DNA CLONING SERVICE, Hamburg, Germany) using *SfiI* restriction sites. The sequence-verified binary vector was introduced into the hypervirulent *Agrobacterium tumefaciens* strain AGL1 via electroporation. A resulting AGL1 clone was then used to inoculate immature embryos of barley cv. “Golden Promise” as previously described (Hensel et al. 2009). To verify the T-DNA transfer, T0 plants were analyzed for the presence of *cas9* and *hpt* transgenes using a PCR–based approach, which was followed by Sanger sequencing of target region–specific PCR amplicon to detect mutations (Supplemental Fig. S9). In total, 29 T0 events (41 sister lines) were generated, and based on the analysis, 8 independent events were selected for further investigation in subsequent generations. In the T1, 24 sister lines per event were germinated and analyzed for mutation by PCR amplification of the target region as described above. Based on the zygosity of the mutation and phenotypic characterization, 2 independent homozygous mutant events (*gt1^CR1^* and *gt1^CR2^*) and an azygous (WT) event were selected for detailed phenotypic analysis in T2. Primers used were listed in Supplemental Table S2.

### Association study and haplotype analysis

We have the whole genome sequenced from 358 6-rowed spring barley accessions. Here, a gene-based association study was performed with 752 SNPs (MAF ≥ 0.03) from the 2 flanking genes of *HvGT1* that covered ∼200 kb of the genomic region. To control for both false positives and false negatives, the FarmCPU model was used to detect SNP trait association under GAPIT (version3) (Liu et al. 2016; Wang and Zhang 2021). We included SNPs for the same interval from 300 diverse barley accessions reported elsewhere (Jayakodi et al. 2020), for which 121 SNPs were identified. SNPs were merged and phased with BEAGLE5.0 with default settings (Browning et al. 2018). Merged accessions were grouped with PHYLIP using the DNADIST method. The tree was visualized with ggtree (Yu 2020). Our association study identified a peak SNP situated closed to an open chromatin region from ∼8 kb upstream of *HvGT1* (Lu et al. 2019). We thus conducted haplotype analysis by considering SNPs surrounding the peak SNP and the ATAC-seq peak encompassing a 5-kb interval from the 358 6-rowed barley accessions (112 SNPs) (Supplemental Data Set S40). TF binding motif was predicted using PlantPAN3.0 (Chow et al. 2018).

### Statistical analysis

Bar plots and box plots and statistical analyses were performed using the GraphPad Prism software, version 9.3.1 (LLC). RNA-seq processing and DEseq2 analysis were conducted in R version 3.4.3. Statistical analysis results are provided as Supplemental Data sets S41–S45 (https://doi.org/doi:10.5061/dryad.j0zpc86k9).

### Accession numbers

RNA-seq data have been deposited at the European Nucleotide Archive (ENA) under accession number PRJEB51523. GenBank accession numbers are *HvGT1*-AK365295, *HvNCED1*-AB239297.1. Supplemental Data sets Supplemental S1 to S45 and phylogenetic analysis alignment files and sequences are openly available in the Dryad Digital Repository at https://doi.org/doi:10.5061/dryad.j0zpc86k9.

## Supplementary Material

koad164_Supplementary_Data

## Supplemental data

The following materials are available in the online version of this article.

**Supplemental Figure S1**. An overview of spike PTD in barley.

**Supplemental Figure S2**. Absolute levels of phytohormones in spike sections of barley cv. Bowman.

**Supplemental Figure S3**. Asparagine and trehalose distribution and relative contents along the spike in cv. Bowman.

**Supplemental Figure S4**. Position-specific transcriptome analysis of dying and viable spike parts in cv. Bowman.

**Supplemental Figure S5**. GO enrichment for differentially expressed transcripts across development in cv. Bowman.

**Supplemental Figure S6**. KEGG pathway enrichment analysis for differentially expressed transcripts.

**Supplemental Figure S7**. GO enrichment analysis for apically enriched genes in cv. Bowman.

**Supplemental Figure S8**. Expression heatmap of apically enriched ABA biosynthesis signaling/response genes in cv. Bowman.

**Supplemental Figure S9**. WGCNA coexpression modules in apical, central, and basal positions in cv. Bowman.

**Supplemental Figure S10**. Expression of *HvGT1* in independent gene expression atlas of developing spike meristems of cv. Bowman.

**Supplemental Figure S11**. Phylogenetic tree of GT1-like proteins in grasses.

**Supplemental Figure S12**. HvGT1 localization and mutation screening in gene-edited *Hvgt1* knockout lines.

**Supplemental Figure S13**. Delayed tip degeneration in *Hvgt1* knockout lines.

**Supplemental Figure S14**. Enhanced differentiation of apical spikelets in *Hvgt1* knockout lines.

**Supplemental Figure S15**. Mutation in barley *GT1* does not contribute to grain yield.

**Supplemental Figure S16**. Tillering effect of barley *GRASSY TILLERS1*.

**Supplemental Figure S17**. PTD in 6-rowed cv. Morex.

**Supplemental Figure S18**. Absolute levels of phytohormone distribution in spike sections of cv. Morex.

**Supplemental Figure S19**. Altered primary metabolism along the spike in cv. Morex.

**Supplemental Figure S20**. Position-specific transcriptome analysis in 6-rowed cv. Morex.

**Supplemental Figure S21**. WGCNA coexpression modules in apical, central, and basal positions in cv. Morex.

**Supplemental Figure S22**. *HvGT1* natural variants enhance spikelet survival.

**Supplemental Table S1**. Tentative identifications of detected molecular ions by MALDI-TOF MSI.

**Supplemental Table S2**. Primers used in this study.

**Supplemental Data Set S1**. Rachis node number from the main-culm spike developmental study in cv. Bowman and cv. Morex.

**Supplemental Data Set S2**. Absolute amounts of primary metabolites quantified in cv. Bowman.

**Supplemental Data Set S3**. Expressed transcript counts from RNAseq analysis (cv. Bowman) used for PCA.

**Supplemental Data Set S4**. TPM values for each expressed transcripts in cv. Bowman.

**Supplemental Data Set S5**. Differentially expressed transcripts (log2FC > 1) and their description identified between 15 pairwise combinations in cv. Bowman.

**Supplemental Data Set S6**. Top 20 enriched GO terms for DETs from apical and central pairwise comparisons in cv. Bowman.

**Supplemental Data Set S7**. GO enrichment analysis for DETs from apical, central, and basal positions across development in cv. Bowman.

**Supplemental Data Set S8**. List of apical region–specific transcripts identified by ROKU analysis in 2-rowed cv. Bowman and their description.

**Supplemental Data Set S9**. List of central region–specific transcripts identified by ROKU analysis in 2-rowed cv. Bowman and their description.

**Supplemental Data Set S10**. List of basal region–specific transcripts identified by ROKU analysis in 2-rowed cv. Bowman and their description.

**Supplemental Data Set S11**. List of putative PTD transcripts in 2-rowed cv. Bowman and their description.

**Supplemental Data Set S12**. GO enrichment for enriched transcripts in central parts in cv. Bowman.

**Supplemental Data Set S13**. GO enrichment analysis for enriched transcripts in basal parts in cv. Bowman.

**Supplemental Data Set S14**. KEGG pathway enrichment analysis for DETs from apical, central, and basal positions across development in cv. Bowman.

**Supplemental Data Set S15**. Stage-wise GO enrichment analysis for apically enriched putative PTD genes in cv. Bowman.

**Supplemental Data Set S16**. Top 20 stage-wise cis-motifs among apically enriched genes in cv. Bowman.

**Supplemental Data Set S17**. Apically enriched senescence-, cell death–, and ROS–related genes and their description in cv. Bowman.

**Supplemental Data Set S18**. Apically enriched ABA biosynthesis signaling/response genes and their description in cv. Bowman.

**Supplemental Data Set S19**. IDs and description of apically enriched TF gene families in cv. Bowman.

**Supplemental Data Set S20**. Apically enriched TF genes rank based on their tissue specificity in cv. Bowman.

**Supplemental Data Set S21**. List of WGCNA coexpressed transcripts in the PTD–associated module in cv. Bowman.

**Supplemental Data Set S22**. GO enrichment analysis for Bowman PTD–associated yellow module genes.

**Supplemental Data Set S23**. List of transcripts coexpressed with *HvS40-like* (undirected network analysis) in cv. Bowman (yellow module).

**Supplemental Data Set S24**. GO enrichment analysis for putative gene targets of *HvS40-like* in Bowman.

**Supplemental Data Set S25**. Gene IDs of *GT1-like* genes from maize, wheat, and barley used for phylogenetic tree construction.

**Supplemental Data Set S26**. List of transcripts coexpressed with *HvGT1* (undirected network analysis) in cv. Bowman (yellow module).

**Supplemental Data Set S27**. Phenotyping scores for WT Golden Promise and *Hvgt1* mutant lines.

**Supplemental Data Set S28**. Expressed transcript counts from RNA-seq analysis (cv. Morex) used for PCA.

**Supplemental Data Set S29**. TPM values for each expressed transcripts in cv. Morex.

**Supplemental Data Set S30**. Differentially expressed transcripts (log2FC > 1) and their description identified between 15 pairwise combinations in cv. Morex.

**Supplemental Data Set S31**. Top 20 enriched GO terms for DETs from apical and central pairwise comparisons in cv. Morex.

**Supplemental Data Set S32**. List of apical region–specific transcripts identified by ROKU analysis in 6-rowed cv. Morex and their description.

**Supplemental Data Set S33**. List of putative PTD transcripts in 6-rowed cv. Morex and their description.

**Supplemental Data Set S34**. List of common PTD transcripts in 2-rowed cv. Bowman and their description.

**Supplemental Data Set S35**. List of common PTD transcript genes in 6-rowed cv. Morex and their description.

**Supplemental Data Set S36**. GO enrichment analysis for apically enriched putative PTD genes in cv. Morex.

**Supplemental Data Set S37**. List of WGCNA coexpressed transcripts in the PTD–associated module in cv. Morex.

**Supplemental Data Set S38**. GO enrichment analysis of Morex PTD–associated dark-cyan module genes.

**Supplemental Data Set S39**. List of transcripts coexpressed with *HvGT1* (undirected network analysis) in cv. Morex (dark-cyan module).

**Supplemental Data Set S40**. 112 SNPs and spikelet survival phenotyping score for the haplotype groups identified in candidate gene association study.

**Supplemental Data Set S41**. Statistical analyses for the data in Fig. 2E.

**Supplemental Data Set S42**. Statistical analyses for the data in Fig. 3B.

**Supplemental Data Set S43**. Statistical analyses for the data in Fig. 7H.

**Supplemental Data Set S44**. Statistical analyses for the data in Fig. 8B.

**Supplemental Data Set S45**. Statistical analyses for the data in Fig. 8F.
