# Supplementary material for: Multilayered regulation of developmentally programmed pre-anthesis tip degeneration of the barley inflorescence
Source: Plant Cell. 2023 Jun 7;35(11):3973–4001. doi: 10.1093/plcell/koad164 (PMC10615218; doi:10.1093/plcell/koad164)
Supplement: koad164_Supplementary_Data [file koad164_supplementary_data.zip › tpc.22.01245Supplemental Figures and Tables.pdf]

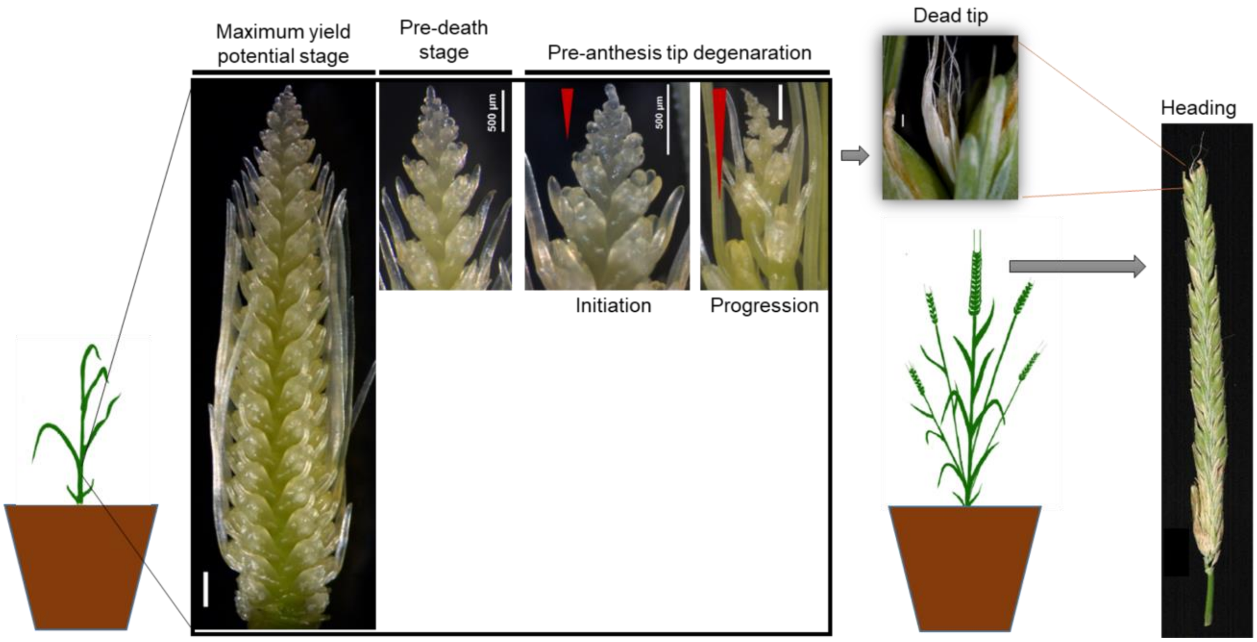


**Supplemental Figure S1. An overview of pre-anthesis tip degeneration in barley.** (Supports Figure 1). At Maximum yield potential stage, inflorescence meristem stops initiating new spikelet primordia. The IM will remain superficially intact until entering into visible death initiation from the tip, which gradually moves in a basipetal fashion. All these events happen when the spike meristems are still covered by the leaves and requires microscopic dissection to visualize the events. At heading, the dead tip can be barely seen as a dried tissue. Red triangle indicates the basipetal pattern of tip degeneration. Scale bar 500 µm.

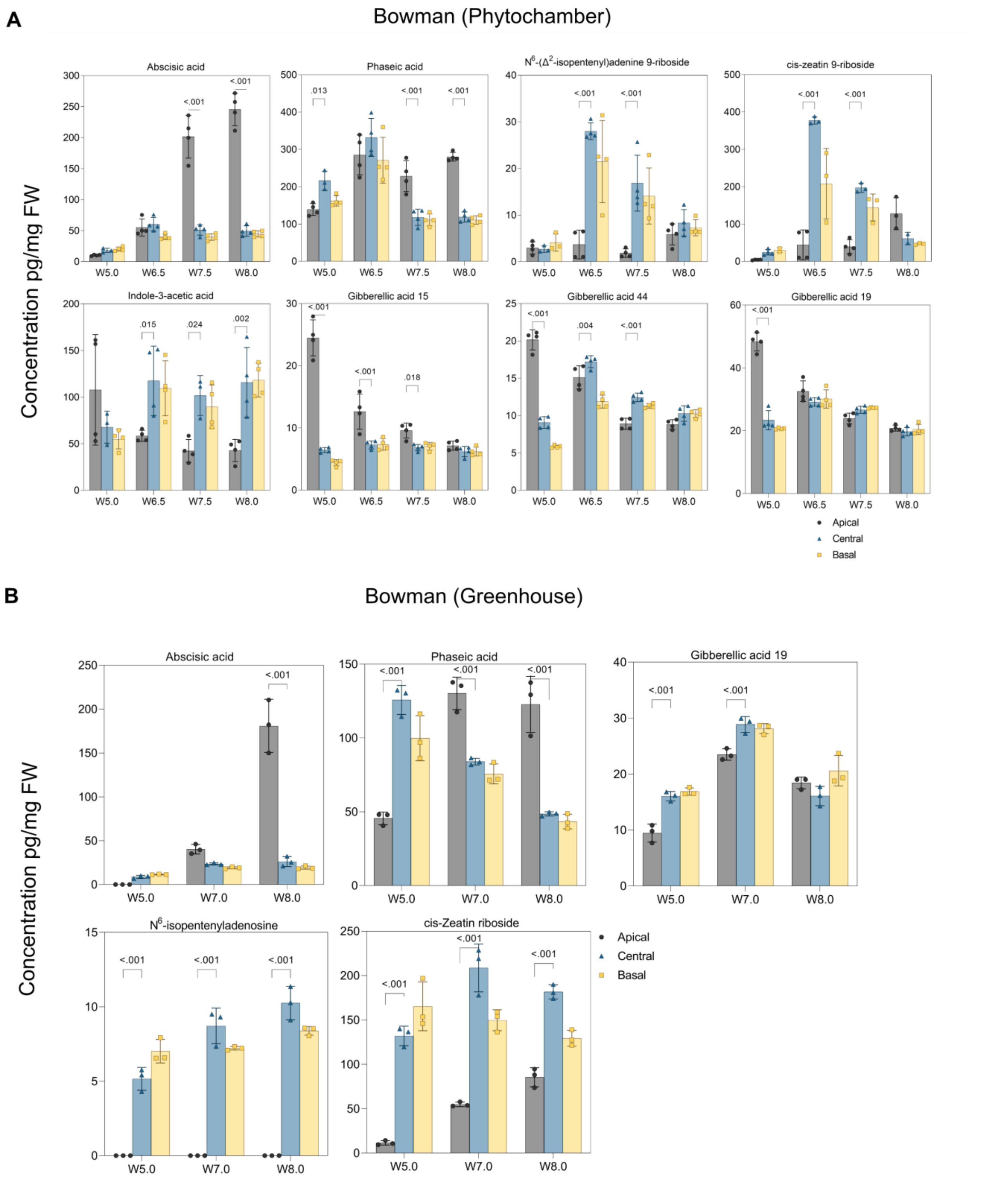


**Supplementary Figure S2. Absolute levels of phytohormones in spike sections of barley cv. Bowman.** (Supports Figure 2). **(A)** Concentrations of hormones along the apical, central and basal spike positions of cv. Bowman grown in the climate chamber. **(B)** Hormone levels in three spikes of Bowman under long-day conditions in the greenhouse. Under greenhouse conditions, visible death initiation happens at around stage W7.0 in Bowman.

Plots show means ± SD calculated from at least three to four biological replicates (Materials & Methods). Statistical analysis (Two-way ANOVA with Tukey's multiple comparison test) was carried out to evaluate the difference between apical, central and basal positions at each stage. W, Waddington scale; cv., cultivar; FW, fresh weight.

**A**

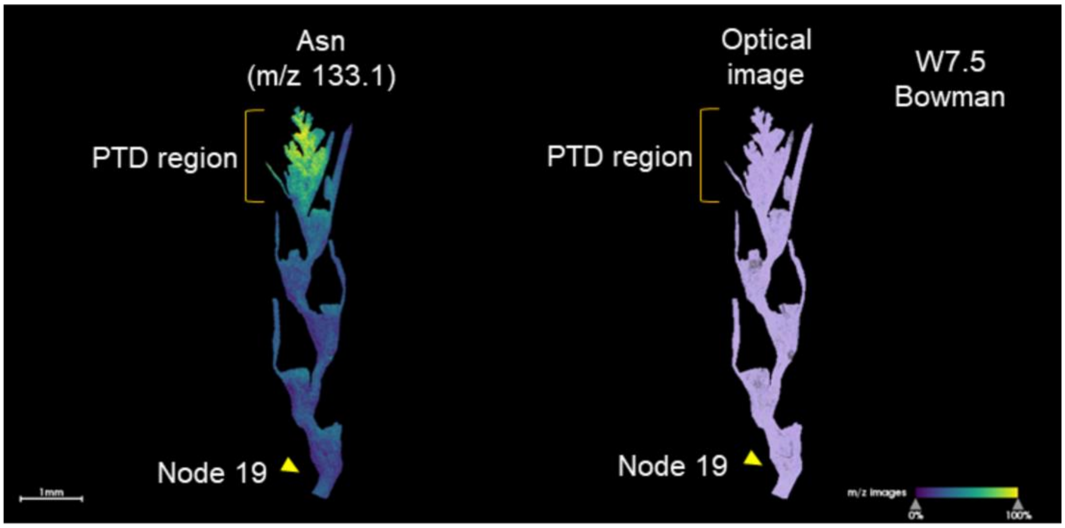


**B**

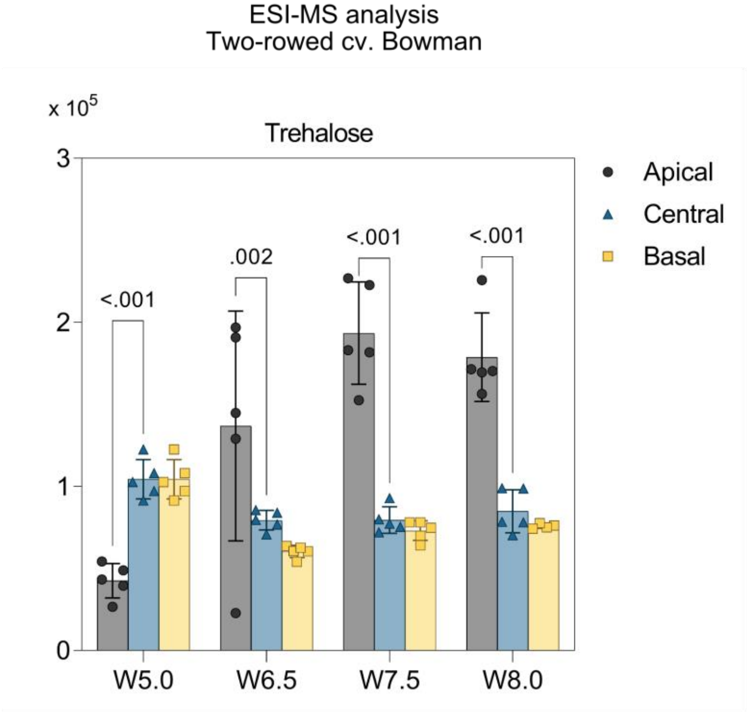


**Supplemental Figure S3. Asparagine and trehalose distribution and relative contents along the spike in cv. Bowman.** (Supports Figure 3). **(A)** Asparagine (Asn, *m/z* 133.1±0.1Da) distributions in spike tissue sections of cv. Bowman at stage W7.5 by MALDI MS imaging. Scale bar 1 mm. The color bar shows the signal intensity of ions; yellow, maximum ion intensity. **(B)** Relative distribution of trehalose in the three spike positions of Bowman measured by ESI-MS (Electrospray ionization – Mass spectrometry) analysis. The relative quantitation of trehalose was based on the most intense ion, $[M+H-H_2O]^+$ *m/z* 325.1138. y-axis shows the relative intensity. Plots show means ± SD

calculated from five biological replicates (Materials & Methods). Statistical analysis (Two-way ANOVA with Tukey's multiple comparison test) was carried out to evaluate the difference between apical, central and basal positions at each stage. PTD, pre-anthesis tip degeneration; W, Waddington scale.

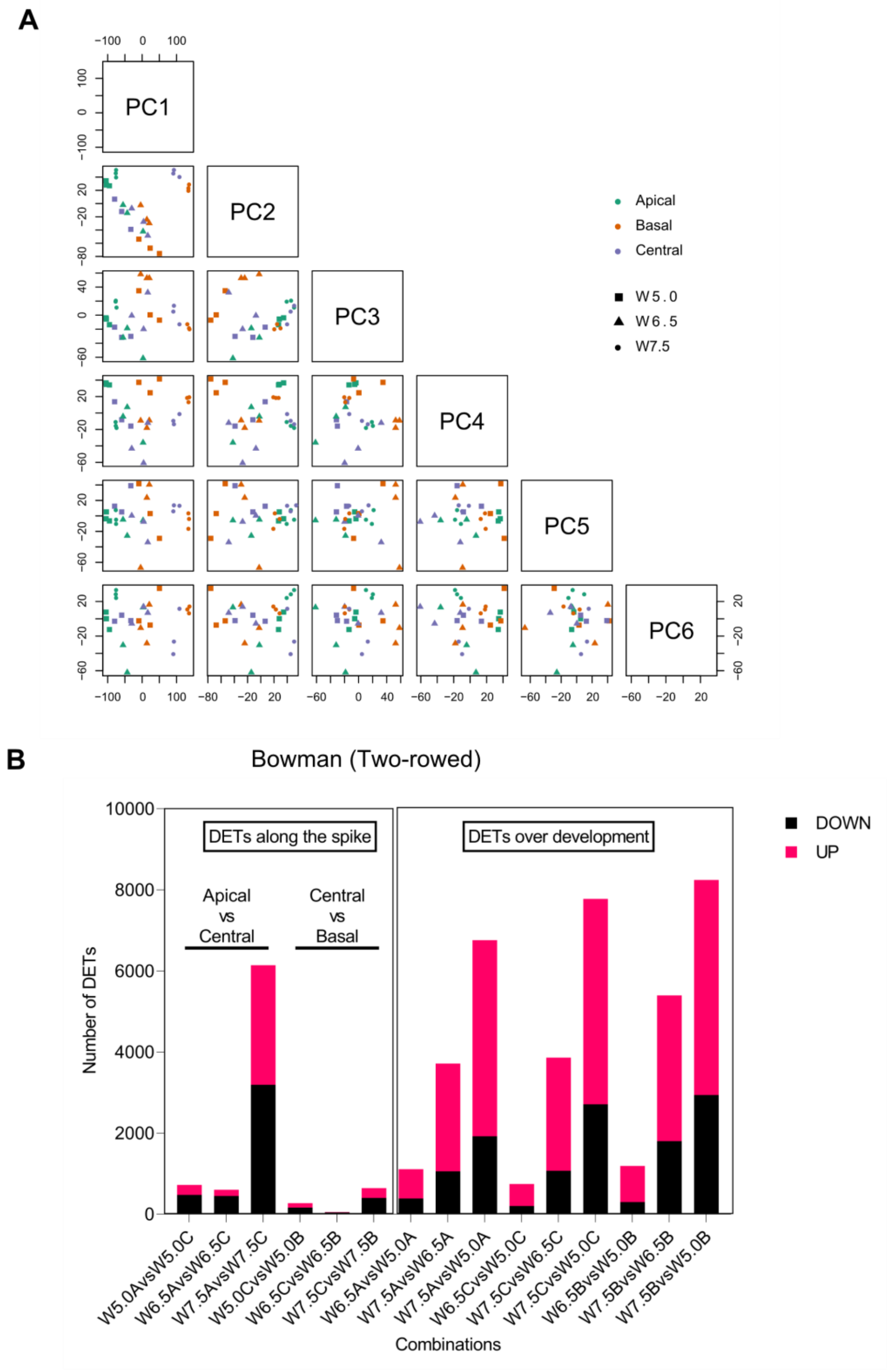


**Supplemental Figure S4. Position-specific transcriptome analysis of dying and viable spike parts in cv. Bowman.** (Supports Figure 4). **(A)** Principal component analysis (PCA) of normalized expression levels (counts per million, cpm) of all expressed transcripts in two-rowed cv. Bowman. Figure shows PC1 to PC6. **(B)** Number of differentially expressed transcripts ($log_2FC > 1$) between 15 pairwise combinations. A- apical, C-central, B- Basal. W, Waddington scale.

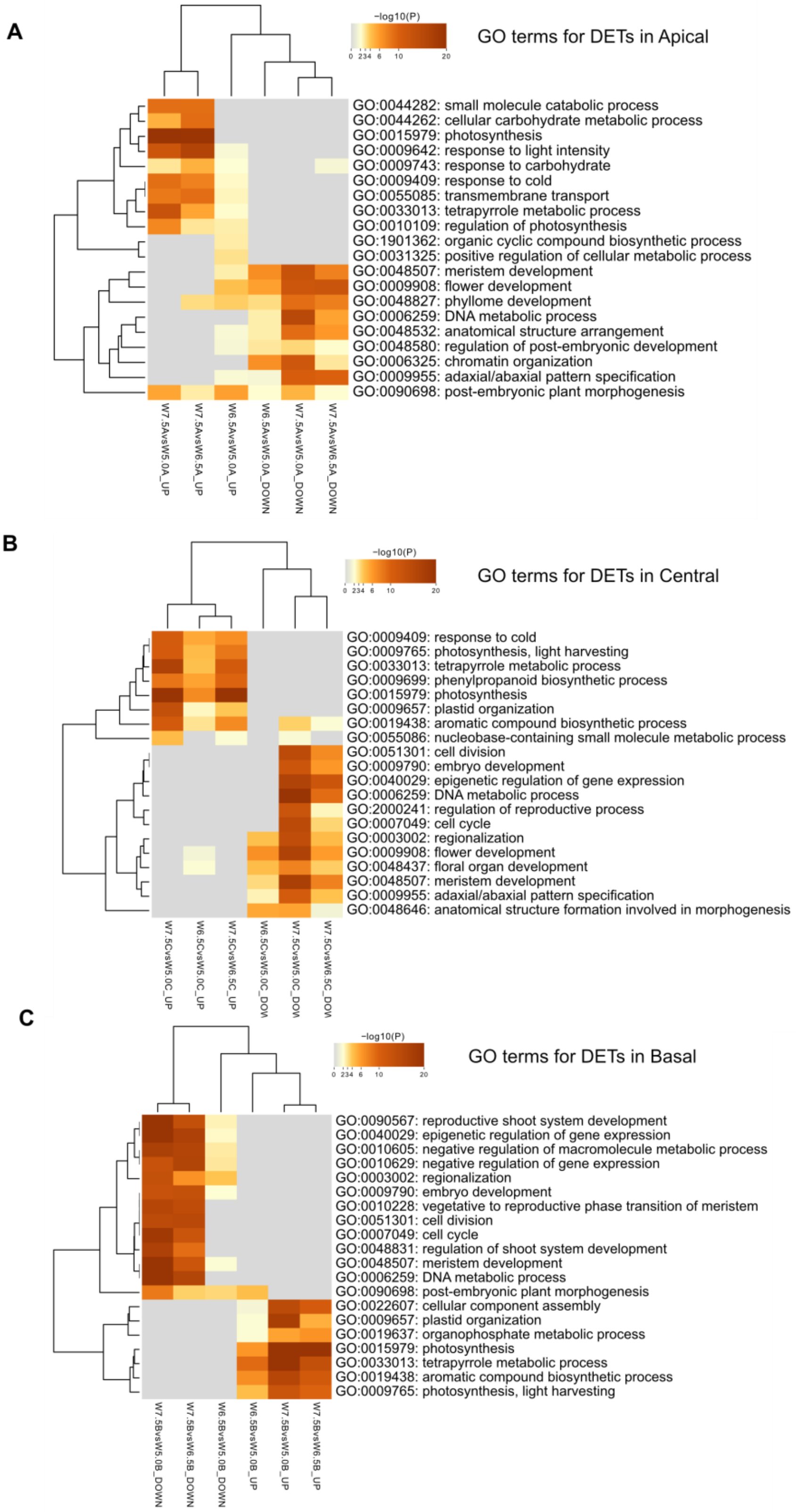


**Supplemental Figure S5. GO enrichment for differentially expressed transcripts across development in cv. Bowman.** (Supports Figure 4). **(A-C)** Top 20 GO terms enriched for the differentially expressed transcripts (DETs) across the development in

apical **(A)**, central **(B)** and basal **(C)** parts in all three stages. Color saturation corresponds to degree of enrichment and the terms were hierarchically clustered based on default settings in Metascape (http://metascape.org). A- apical, C-central, B- Basal. W, Waddington scale.

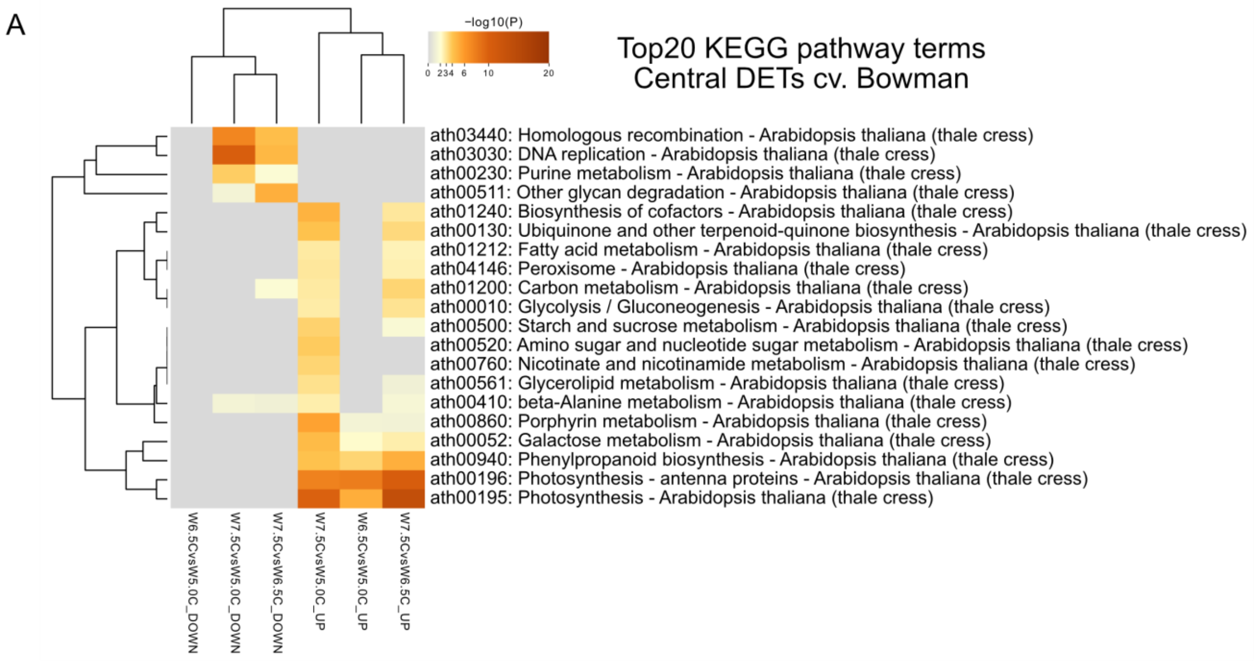


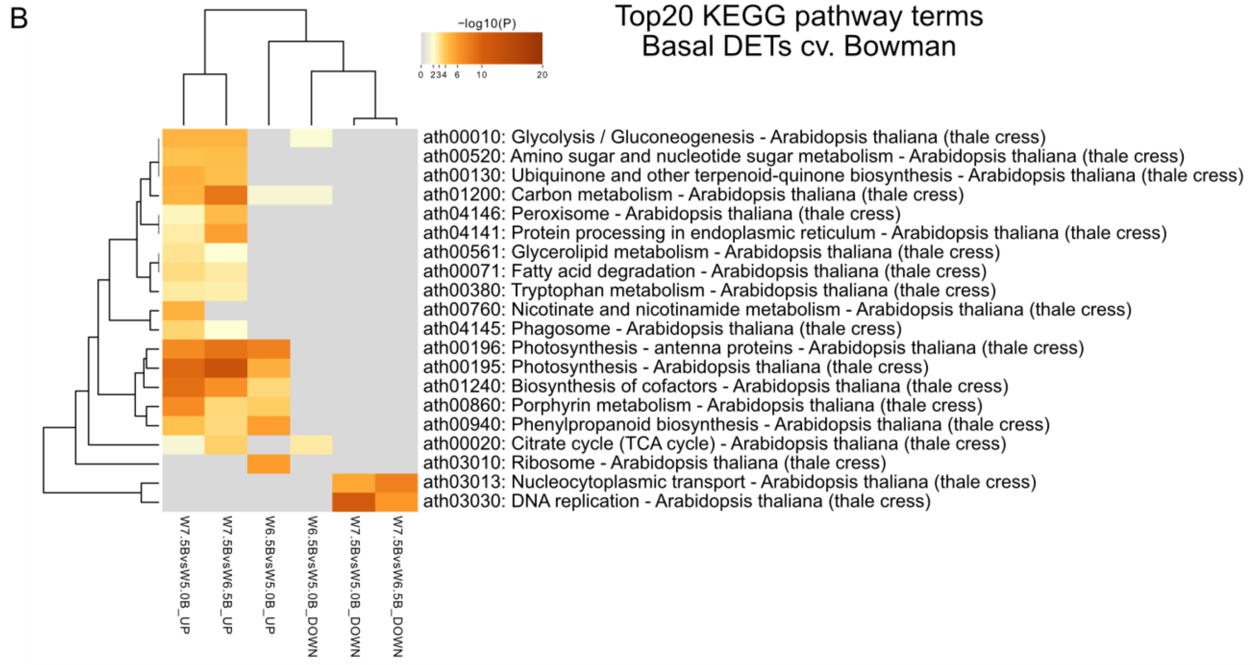


**Supplemental Figure S6**. **KEGG pathway enrichment analysis for differentially expressed transcripts.** (Supports Figure 4). **(A-B)** The heatmap shows the top 20 KEGG pathway terms enriched for the differentially expressed transcripts in spike central **(A)** and Basal **(B)** parts across development in cv. Bowman. Color saturation corresponds to degree of enrichment and the terms were hierarchically clustered based on default settings in Metascape (http://metascape.org). C-central, B- Basal. W, Waddington scale; cv., cultivar.

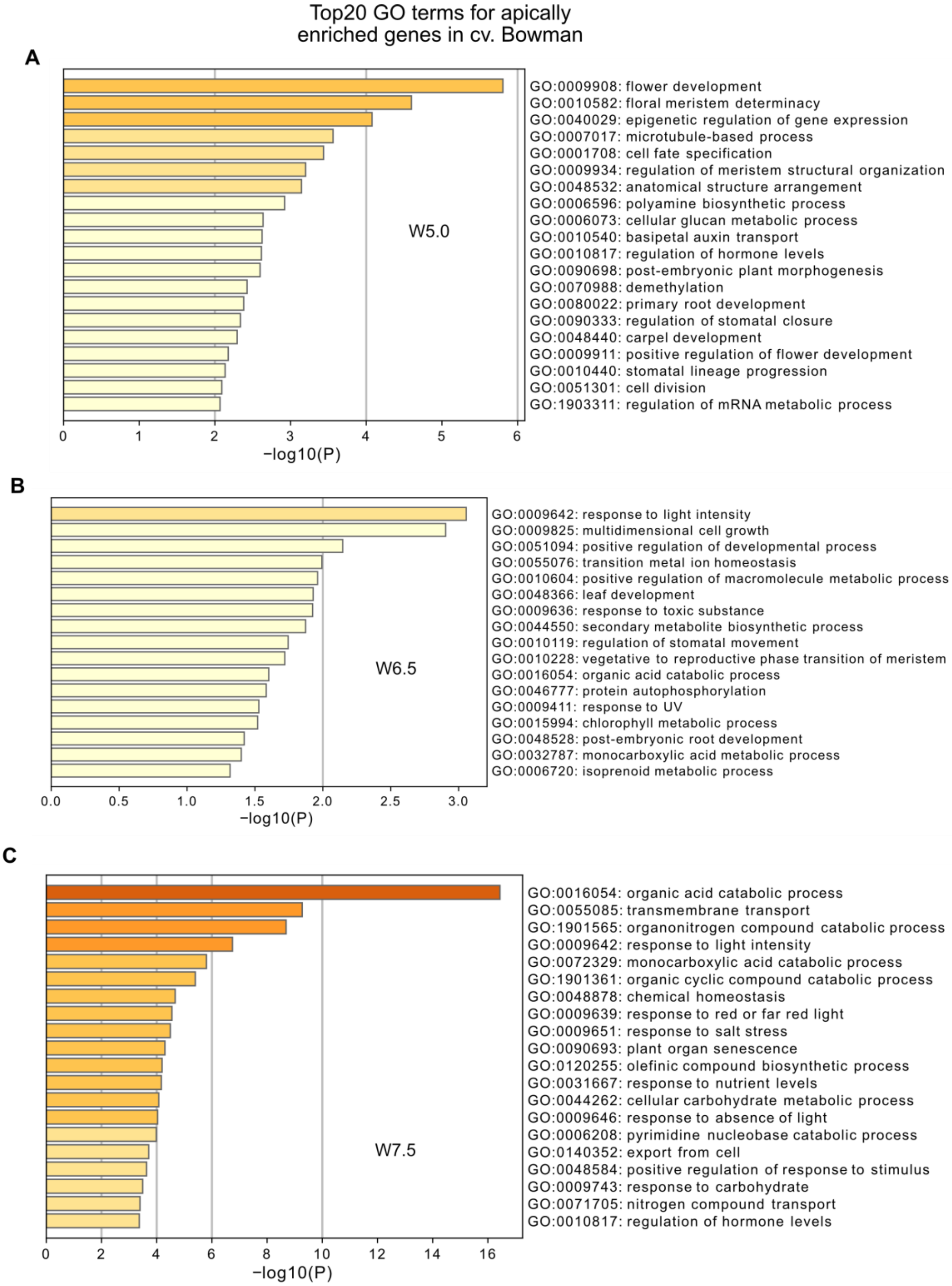


**Supplemental Figure S7. Gene Ontology (GO) enrichment analysis for apically enriched genes in cv. Bowman.** (Supports Figure 4). **(A-C)** Stage-wise enrichment of top 20 biological processes terms. Color saturation corresponds to degree of enrichment. W, Waddington scale.

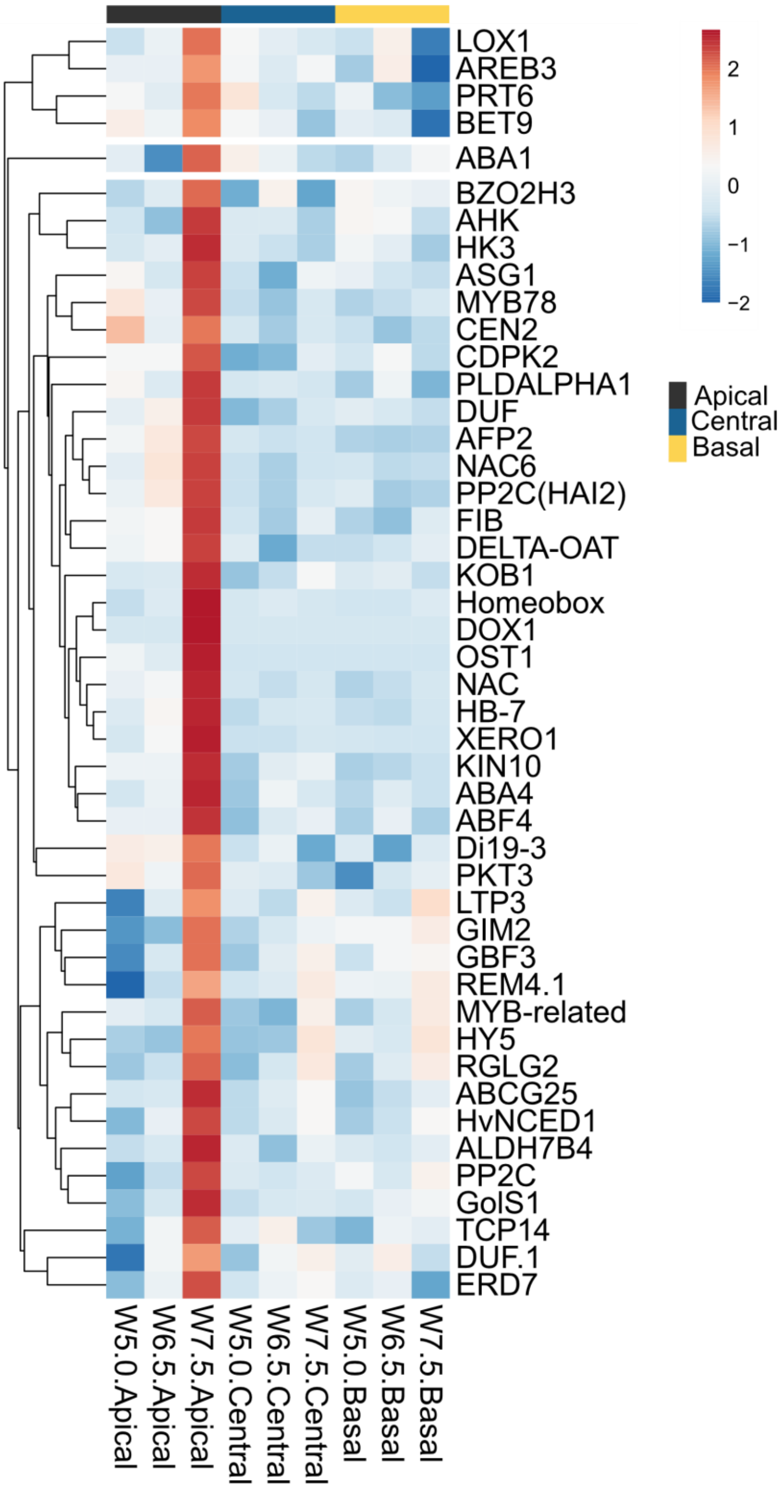


**Supplemental Figure S8. Expression heatmap of apically enriched ABA biosynthesis signaling/response genes in cv. Bowman.** (Supports Figure 4). Heatmap represents ln(X+1) transformed mean TPM values with Euclidian distance (average clustering) and are depicted by color code: red, high; blue, low expression. W, Waddington scale. Respective gene IDs are provided in Supplemental data Set S18.

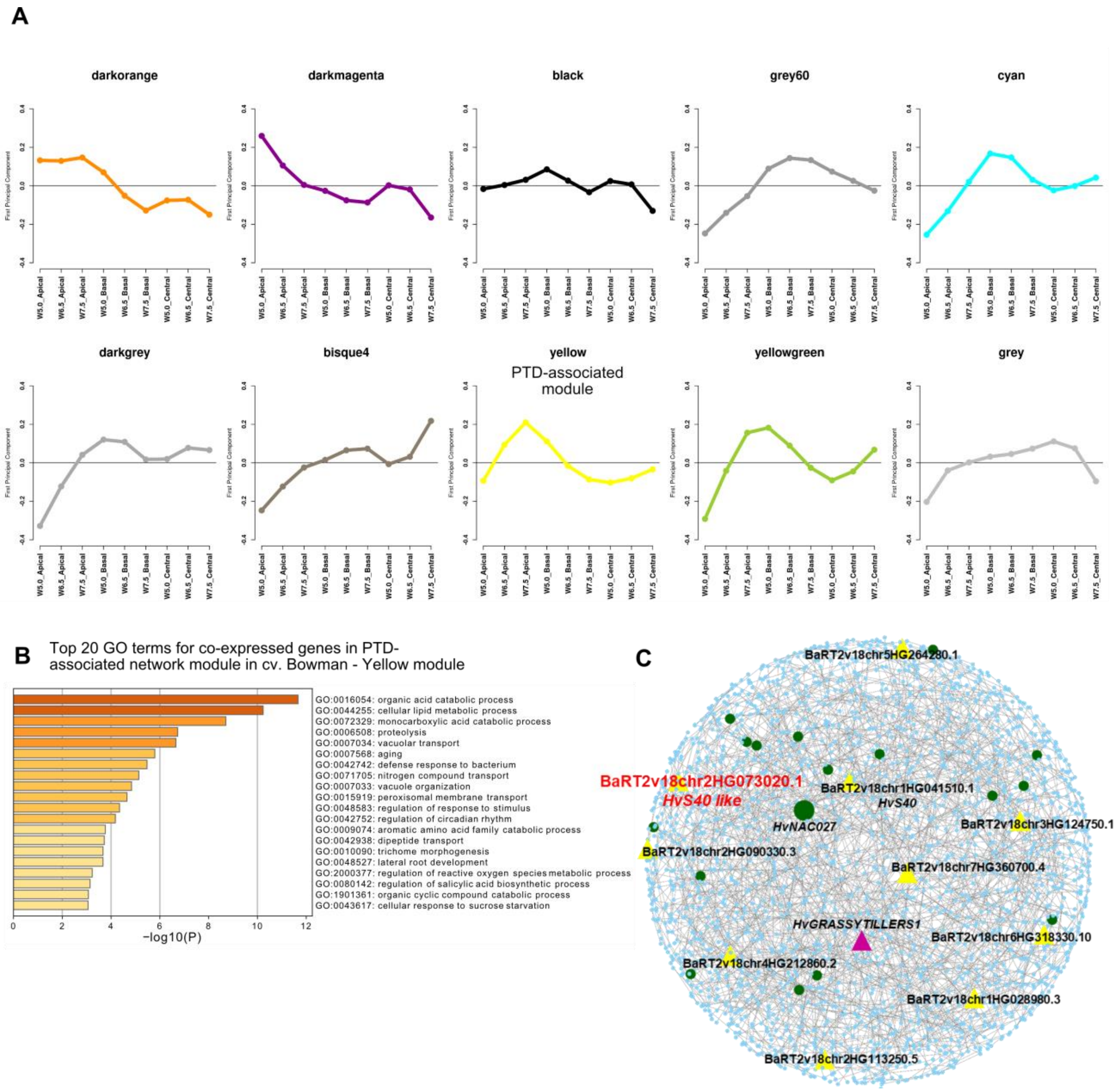


**Supplemental Figure S9. WGCNA co-expression modules in apical, central, and basal position-specific spike transcriptome of cv. Bowman.** (Supports Figure 6). **(A)** Expression patterns of 10 merged modules, generated based on correlation among the original modules in cv. Bowman. Yellow module was considered as a PTD-associated module. **(B)** Top 20 gene ontology (GO) terms enriched in the PTD-associated module (yellow). **(C)** Gene regulatory networks of PTD-associated module in Bowman. The top10 hub genes are presented as yellow triangle and senescence-associated gene *HvS40-like* gene is highlighted in red. Magenta colored triangles highlight *HvGT1* and NAC transcription factors in green circles. W, Waddington scale. Related data in Supplemental Data Set S21-S22.

*Hv GRASSY TILLERS1(HvGT1)*

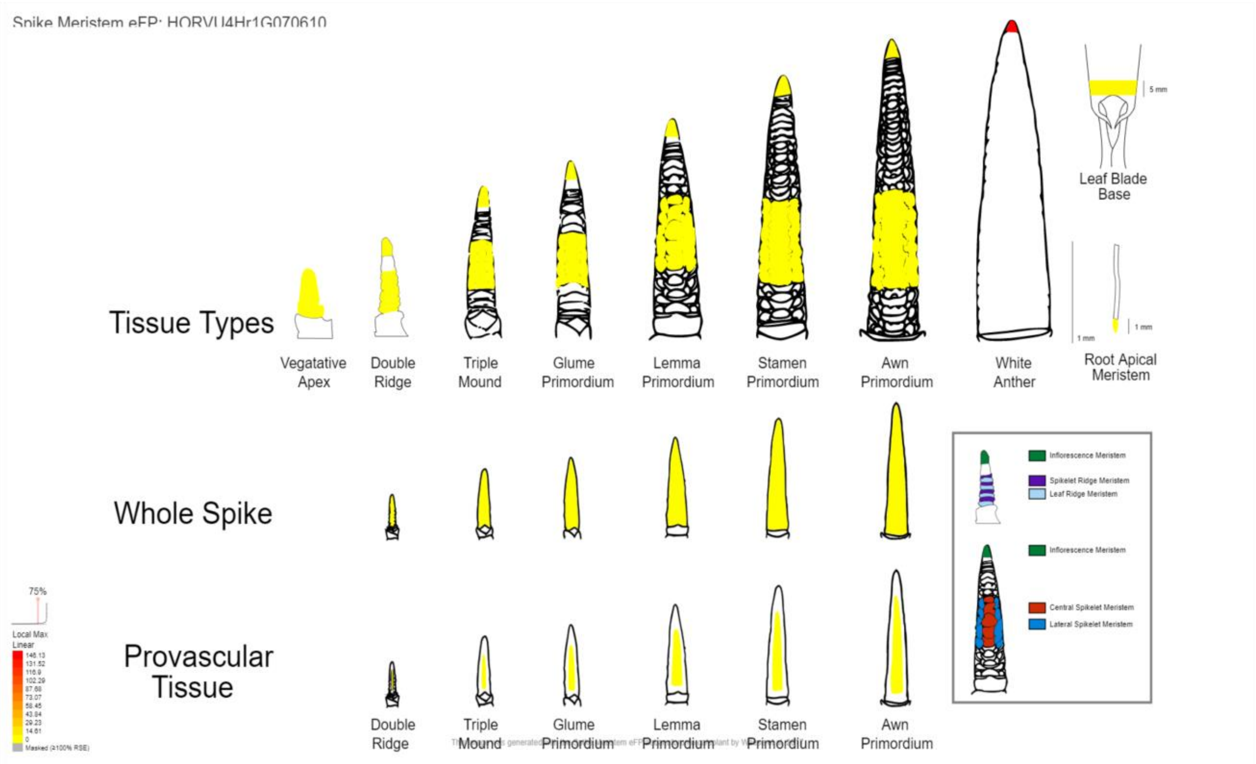


**Supplemental Figure S10. Expression of *HvGT1* in independent gene expression atlas of developing spike meristems of cv. Bowman.** (Supports Figure 7). IM dome-specific expression of *HvGT1* in barley spike meristem at white anther stage (W5.0) in cv. Bowman depicted in the top panel, while weak or no expression in other tissue types. Color scale represents gene expression levels. (http://bar.utoronto.ca/eplant_barley/). IM, Inflorescence meristem.

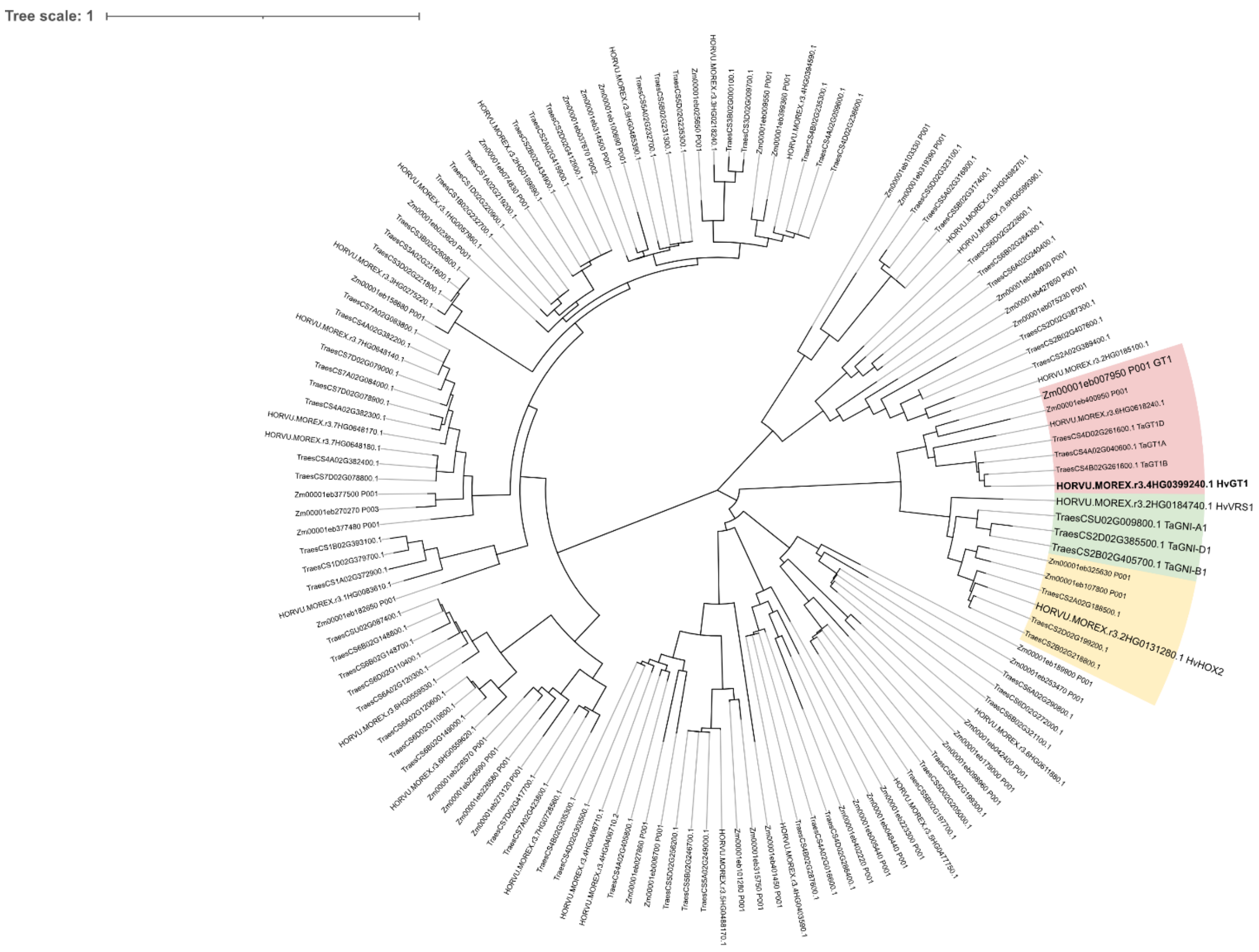


**Supplemental Figure S11. Phylogenetic tree of GT1-like proteins in grasses.** (Supports Figure 7). Protein sequences of GT1 and VRS1 orthologs from barley, wheat and maize were used as queries to blastp against maize (source: Ensembl), Wheat (source: Ensembl) and barley (source: Morex v3 in IPK) protein databases with an e-value cutoff of 1e$^{-10}$. Phylogenic tree was constructed with a Neighbor-Joining method in MEGA using the JTT model, and 1000 bootstrapped replicates. Scale bar refers to phylogenetic distance. GT1 clade shaded with red, VRS1 clade with green and HOX2 clade with yellow. In addition, we found one duplicated GT1 in barley on Chr6H (Low confidence gene - HORVU.MOREX.r3.6HG0618240.1). However, this gene did not express in any of our spike gene expression datasets and as well as in publicly available databases. GeneIDs in Supplemental Data Set S25.

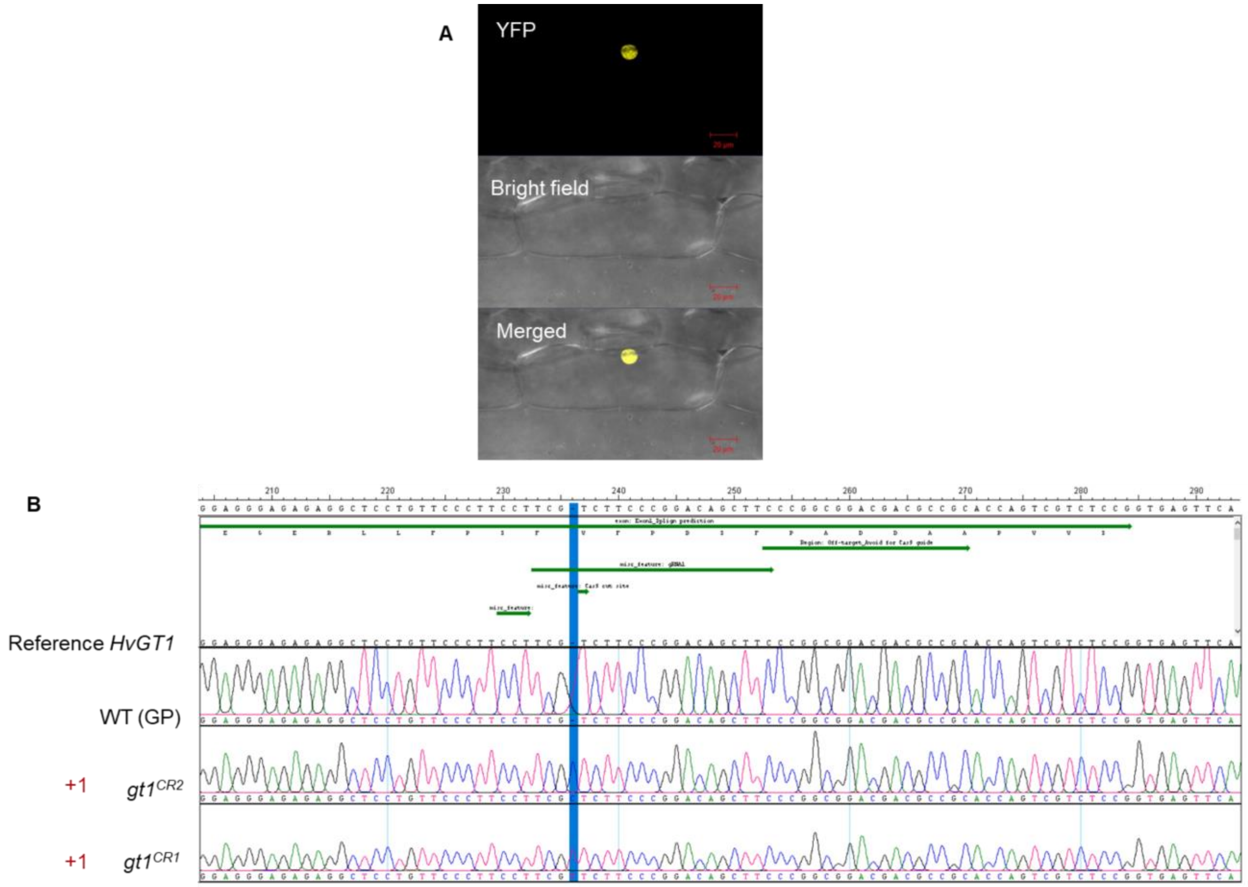


**Supplemental Figure S12. HvGT1 localization and mutation screening in gene-edited *Hvgt1* knockout lines.** (Supports Figure 7). **(A)** HvGT-YFP fusion protein targets to the nucleus in barley leaf epidermis. **(B)** DNA sequences of independent T1 lines (WT, *gt1*$^{CR1}$ & *gt1*$^{CR2}$) in the background of two-rowed cv. Golden Promise (GP). WT, Wild type.

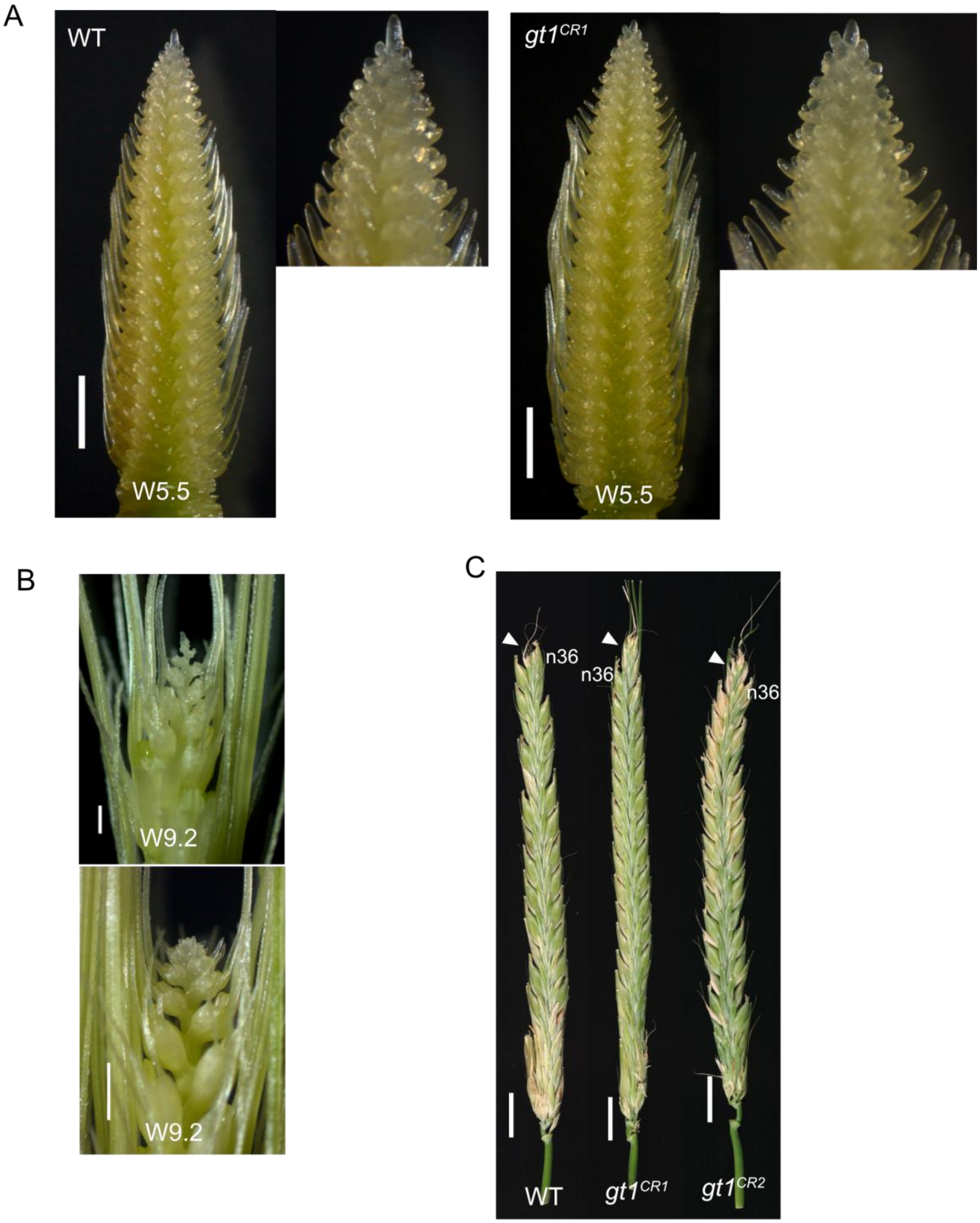


**Supplemental Figure S13. Delayed tip degeneration in *Hvgt1* knockout lines.** (Supports Figure 7). **(A)** Representative stereomicroscope images of WT and $gt1^{CR1}$ spikes. At the MYP stage (W5.5), no significant difference was found between WT and mutant spike. Scale bar 1 mm. **(B)** Mutant W9.2 in the bottom panel shows an intact apical part compared to degenerated WT spike in the top panel. Scale bar 500 µm. **(C)** Whole spikes of WT and *gt1* mutants ($gt1^{CR1}$ & $gt1^{CR2}$) at heading. The white arrow points to the region above node 36 that differs between WT and mutants. Scale bar 1 cm. W, Waddington scale; n, rachis node; MYP, maximum yield potential; WT, wild type.

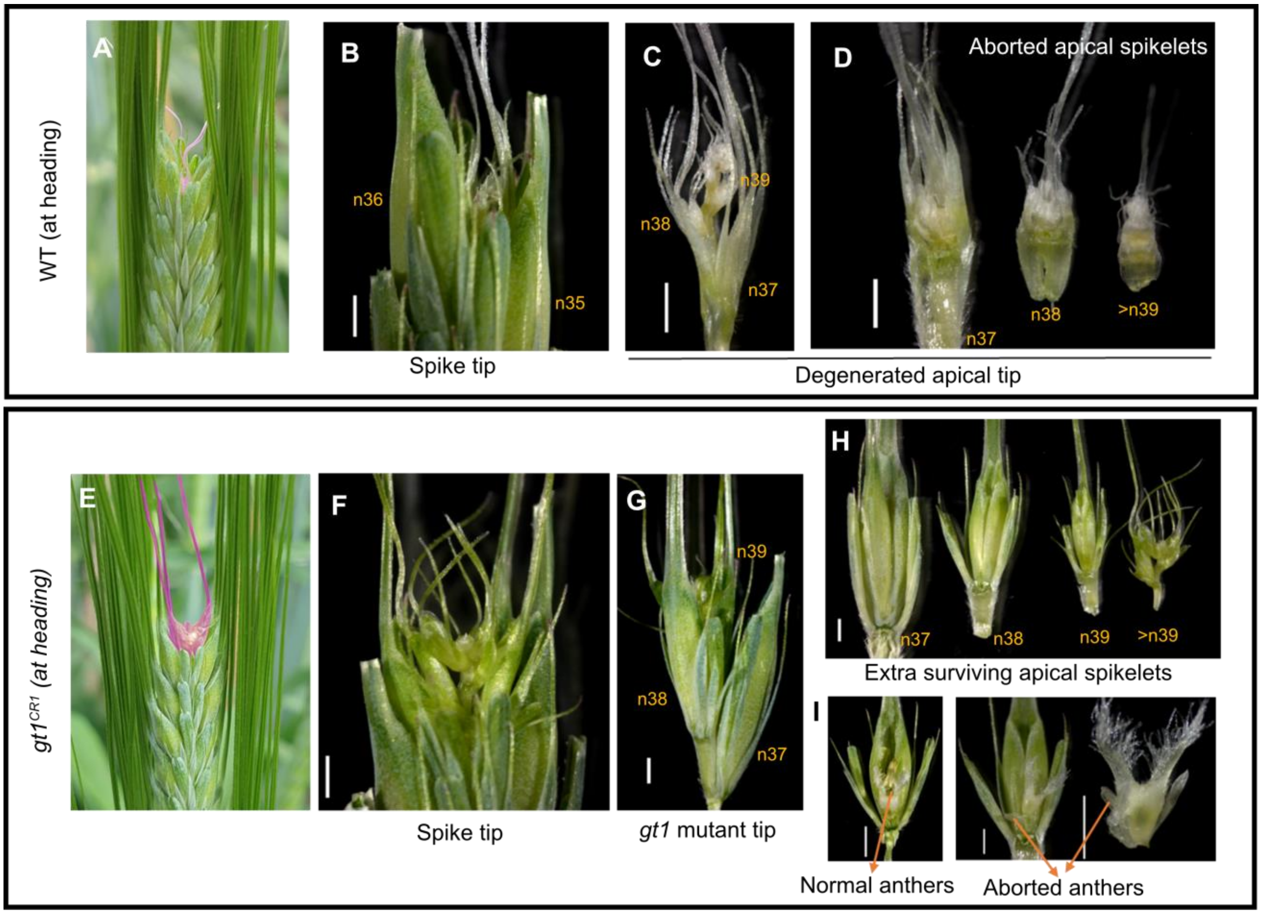


**Supplemental Figure S14. Enhanced differentiation of apical spikelets in *Hvgt1* knockout lines.** (Supports Figure 7). **(A-D)** Wild type (WT) spike at heading and the images shows dissected aborted tip and spikelet remnants **(D)**. **(E-I)** *Hvgt1* mutant spike at heading (extra-surviving spikelets shaded in magenta) and the images show dissected tip and extra-surviving spikelets **(H)**. However, the fertility of the few extra surviving spikelets might differ with viable and aborted anthers as highlighted with orange arrows **(I)**. Scale bar 1 mm. n, rachis node.

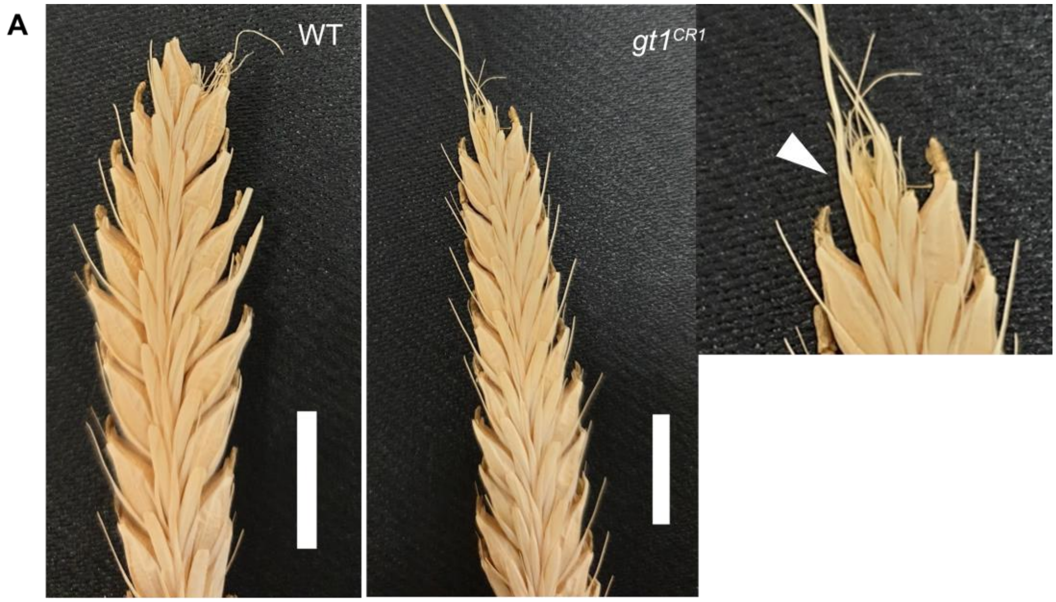


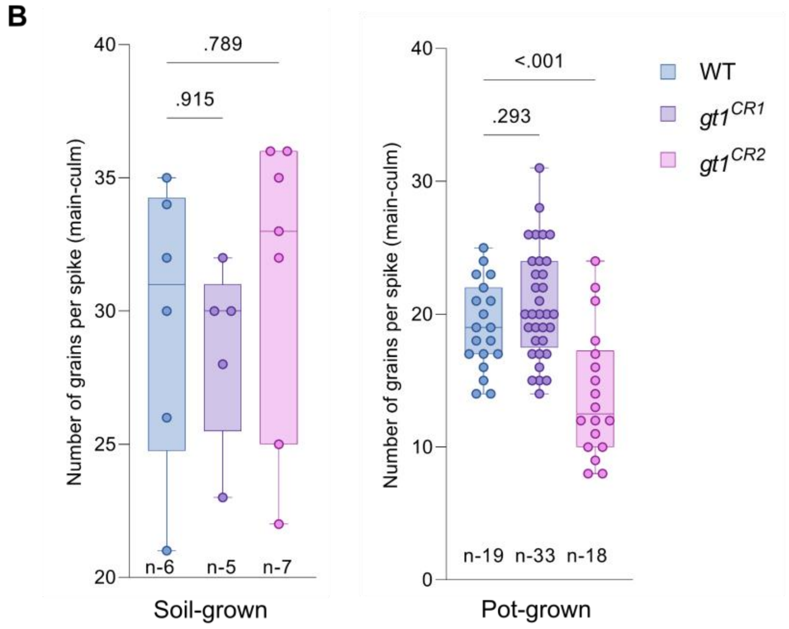


**Supplemental Figure S15. Mutation in barley *GT1* does not contribute to grain yield.** (Supports Figure 7). **(A)** Mature spikes of azygous WT and *gt1* mutant at harvest. White arrow points the unfilled chaff of extra-survived spikelets in *gt1* mutant. Scale bar, 1 cm. **(B)** Box plot shows the final grain number after harvest in the main-culm spikes of WT and *gt1* mutant lines (Materials & Methods; Supplemental Data Set S27). Box plots shows all individual data points with whiskers covering minimum and the maximum values; horizontal line in the box represents the median. The numerical values below each box represents the number of spikes analyzed. Significant levels are determined from the Unpaired Student's *t*-test with Welch's correction. WT, Wild type.

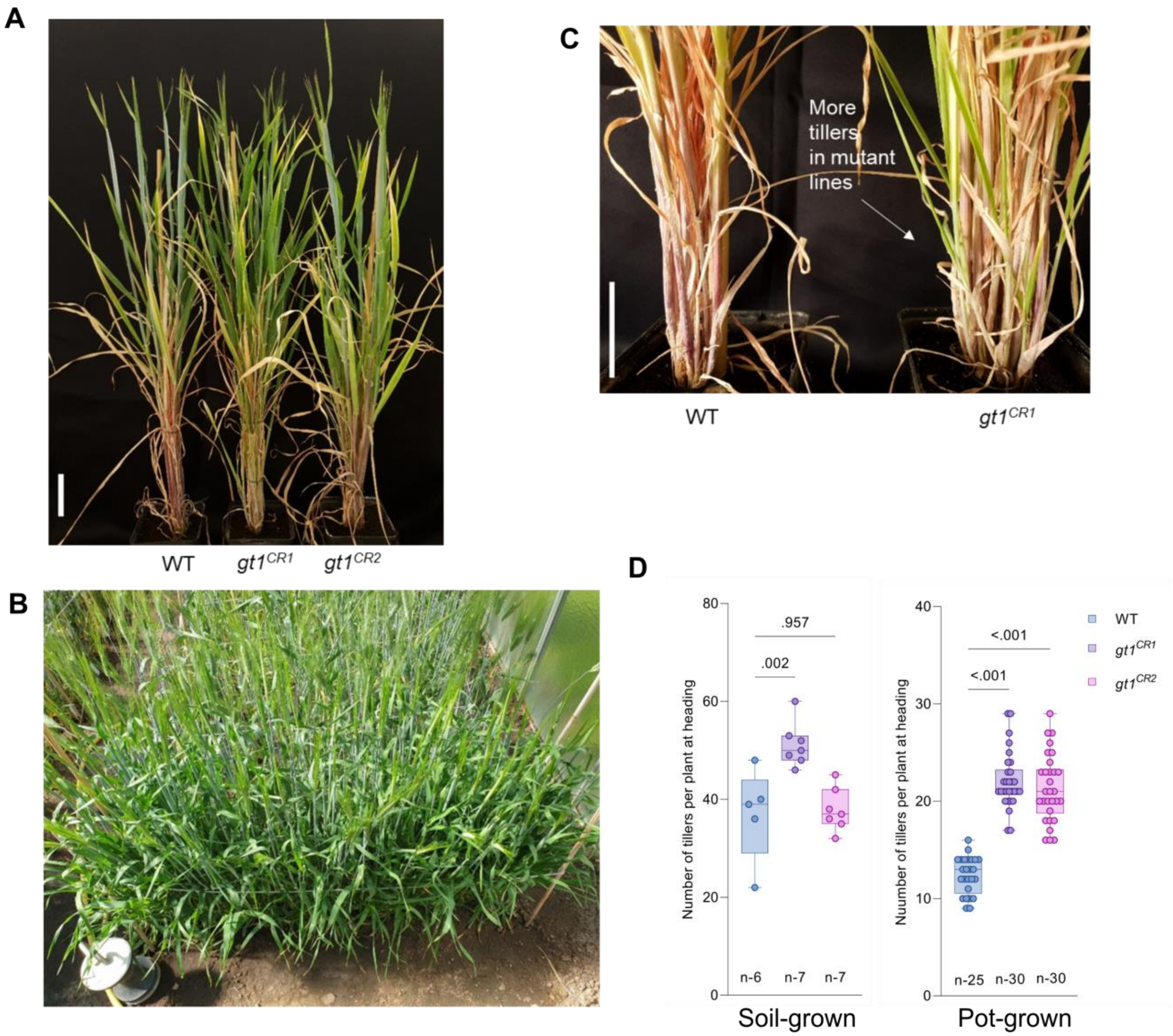


**Supplemental Figure S16. Tillering effect of barley *GRASSY TILLERS1.*** (Supports Figure 7). **(A)** Wild-type (WT) and *Hvgt1* knock-out mutants in two-rowed cv. Golden Promise at heading. **(B)** WT and *gt1* mutant lines at heading growing in the greenhouse in soil. **(C)** Pronounced tillering in *gt1* mutant lines compared to the azygous WT. **(D)** Phenotypic difference in the number of tillers in WT and *gt1* mutant lines (*gt1*$^{CR1}$ & *gt1*$^{CR2}$) in soil-grown and pot-grown plants. Box plots shows all individual data points with whiskers covering minimum and the maximum values; horizontal line in the box represents the median. The numerical values below each box represents the number of spikes analyzed. Significant levels are determined from unpaired Student's *t*-test with Welch's correction. W, Waddington scale. Scale bar 5 cm.

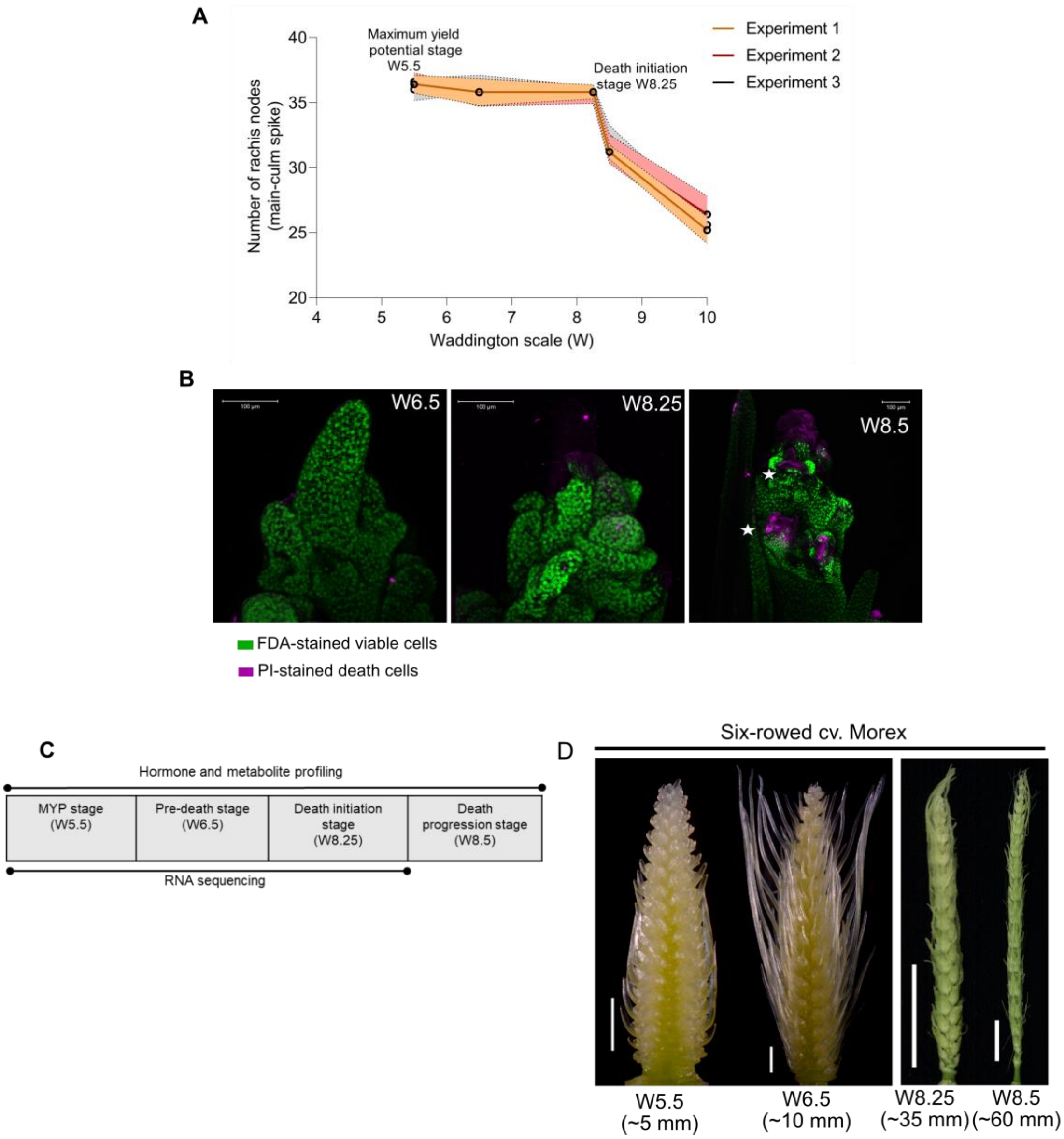


**Supplemental Figure S17. Pre-anthesis tip degeneration in six-rowed cv. Morex.** (Supports Figure 8). **(A)** Spike developmental pattern in the cv. Morex. Plots represent mean and error with a 95% confidence interval for five main-culm spike meristems at each developmental stages from three independent experiments in standardized phytochamber conditions (Materials and Methods; Supplemental Data Set S1). The x-axis indicates stages, and the y-axis shows the number of rachis nodes produced in the main-culm spike. **(B)** Live-death staining assay using Fluorescence diacetate (FDA) and Propidium Iodide (PI) labeling. FDA (green) stains viable cells, and cells undergoing death were stained with PI (Magenta). Asterisk points to the death of anthers in the spikelets at stage W8.5. Scale bar 100 µm. **(C)** Table showing the description of stages used for hormones, metabolome (all four stages), and transcriptome profiling (first three stages until death initiation). **(D)** Four spike developmental stages and their corresponding lengths of cv. Morex used in this study. Scale bar 1 mm (W5.5, W6.5); 1 cm (W8.25, W8.5). W, Waddington scale.

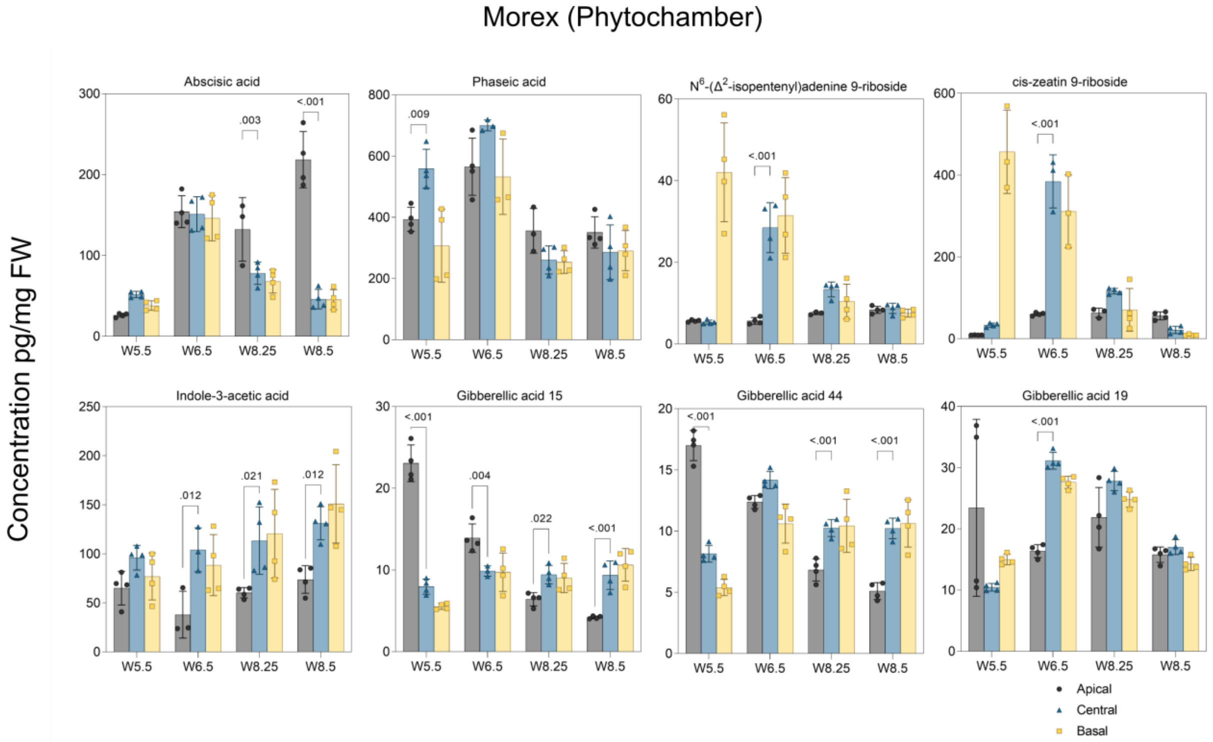


**Supplemental Figure S18. Absolute levels of phytohormone distribution in spike sections of cv. Morex.** (Supports Figure 8). Concentrations of hormones along the apical, central and basal spike positions of cv. Morex grown in the climate chamber. Plots show means ± SD calculated from at least three to four biological replicates. Statistical analysis (Two-way ANOVA with Tukey’s multiple comparison test) was carried out to evaluate the difference between apical, central and basal positions at each stage. W, Waddington scale; FW, fresh weight.

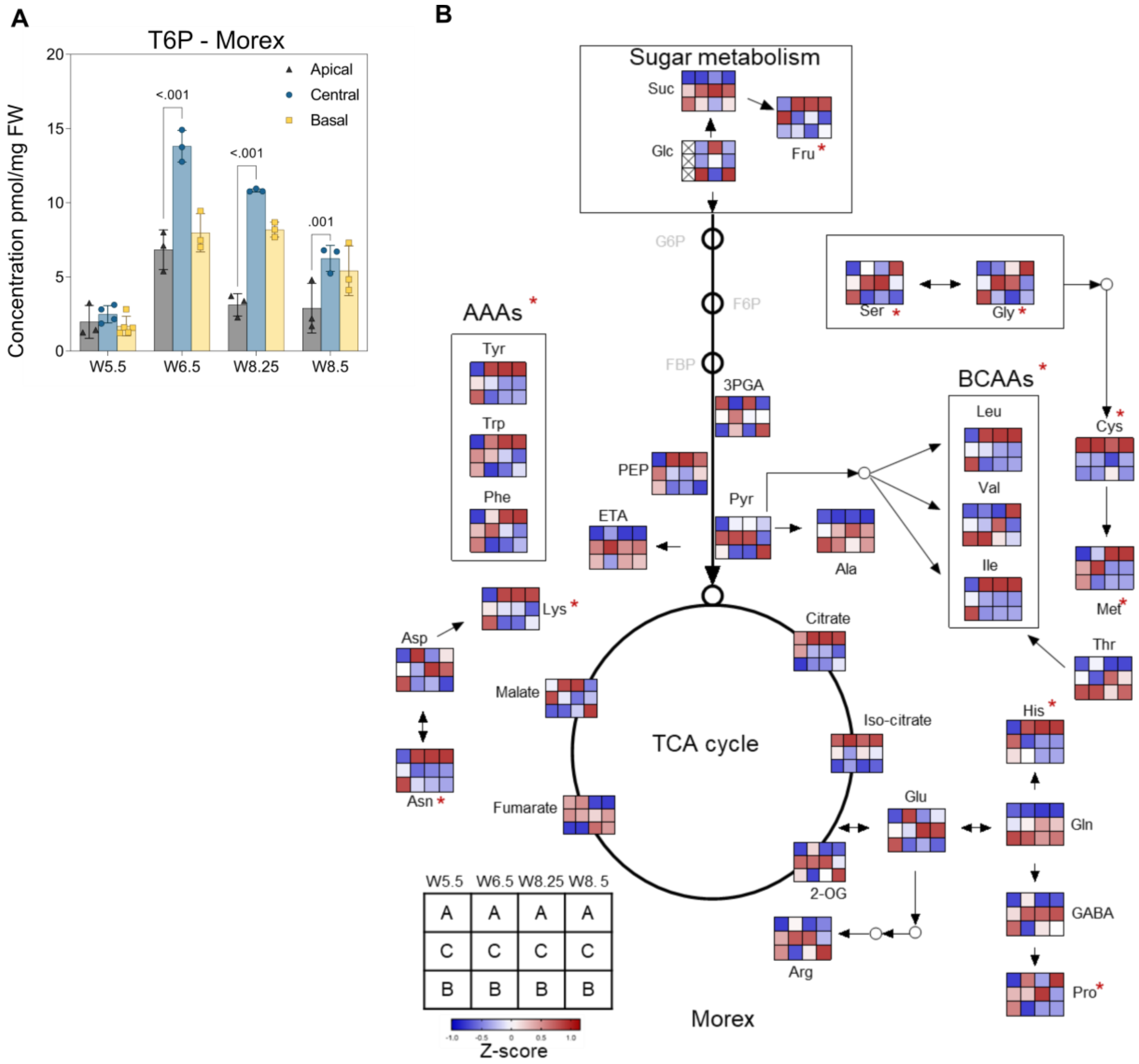


**Supplemental Figure S19. Altered primary metabolism along the spike in cv. Morex.** (Supports Figure 8). **(A)** Graph show the absolute levels of sugar phosphate T6P (trehalose-6-phosphate) in different spike parts of Morex. Plots show means ± SD calculated from three to five biological replicates. The y-axis represents absolute units of measurement. Statistical analysis (Two-way ANOVA with Tukey's multiple comparison test) was carried out to evaluate the difference between apical, central and basal positions at each stage. **(B)** Heatmap of primary metabolite changes during spike growth phase. Colors represents the Z-transformed ratios of apical, central and basal parts at each stage. Red - High; Blue - low. Suc, sucrose; Glc, glucose; Fru, fructose; Asn, asparagine; Asp, aspartate; Arg, arginine; Ala, Alanine; Glu, glutamate; Gln, glutamine, Cys, cysteine, Met, methionine; Gly, glycine; Ser, serine; His, histidine; Val, valine; Leu, Leucine; Ile, isoleucine; Phe, phenylalanine; Tyr, tyrosine; Trp, tryptophan, Thr, threonine; Lys, lysine; GABA, gamma aminobutyric acid; Pro, proline; 3-PGA, 3-Phosphoglyceric acid; PEP, phosphoenolpyruvate; Pyr, pyruvate; 2-OG, 2-Oxoglutarate; AAAs, aromatic amino acids; BCAAs, branched-chain amino acids; TCA, tri-carboxylic acid; W, Waddington scale. Red asterisk points the amino acids and sugar significantly enriched at high levels in the apical part during PTD. PTD, pre-anthesis tip degeneration; W, Waddington scale; FW, fresh weight.

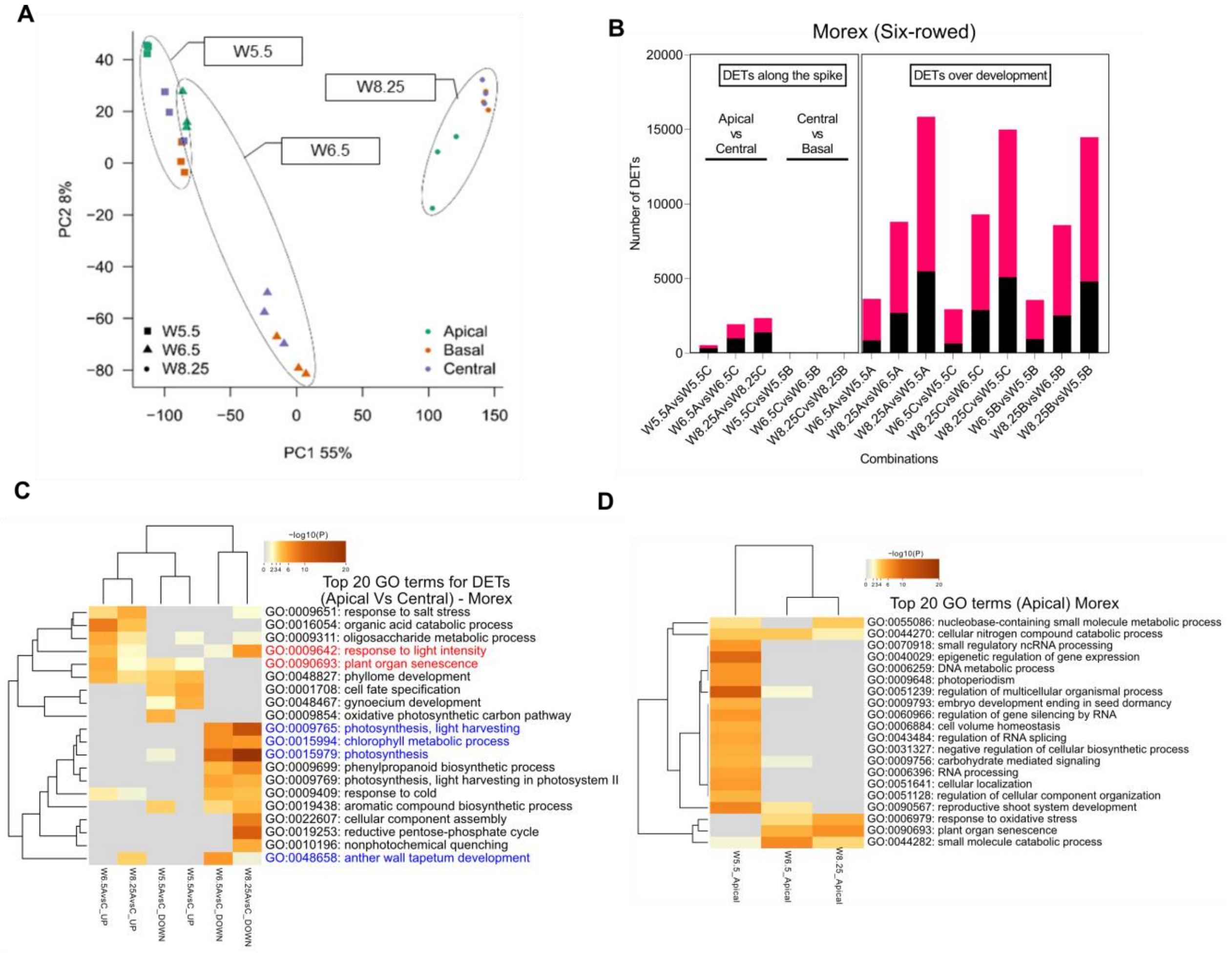


**Supplemental Figure S20. Position-specific transcriptome analysis in six-rowed cv. Morex.** (Supports Figure 8). **(A)** Principal component analysis (PCA) of normalized expression levels (counts per million, cpm) of all expressed transcripts. **(B)** Number of differentially expressed transcripts ($log_2$ FC > 1) between 15 pairwise combinations. **(C)** Top 20 GO terms enriched for the differential expressed transcripts (DETs) between degenerating apical and viable central parts. Biological processes associated with spike PTD were highlighted in red and those related to spike growth were highlighted in blue. **(D)** Top 20 GO terms enriched for the apically enriched transcripts. Color saturation corresponds to the degree of enrichment and the terms were hierarchically clustered based on default settings in Metascape (http://metascape.org). A- apical, C-central, B-Basal. W, Waddington scale; PTD, Pre-anthesis tip degeneration.

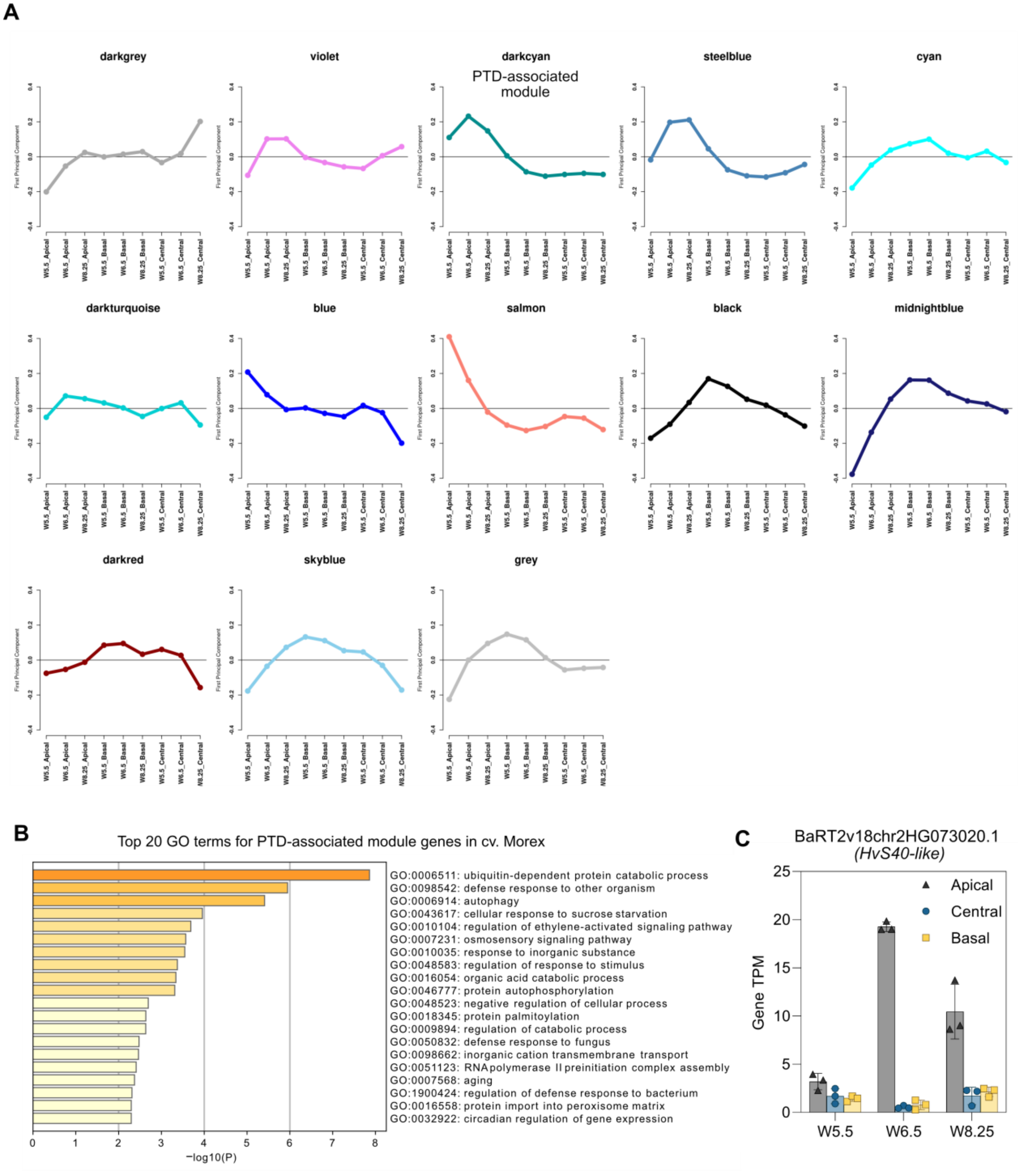


**Supplemental Figure S21. WGCNA co-expression modules in apical, central, and basal position-specific spike transcriptome of cv. Morex.** (Supports Figure 8). **(A)** Expression patterns of 10 merged modules, generated based on correlation among the original modules. Darkcyan module was considered as a degeneration-associated module which include *HvGT1* and *HvS40-like* genes. **(B)** Top 20 terms enriched in the PTD-associated module. Color saturation corresponds to the degree of enrichment. **(C)** Bar

plots show the expression of the senescence-associated gene, *HvS40-like* in Morex, which has been identified as a key hub gene in Weighted gene co-expression network analysis (WGCNA). Plots show means ± SD of TPM values from three biological replicates (Materials & Methods). W, Waddington scale; TPM, transcript per million.

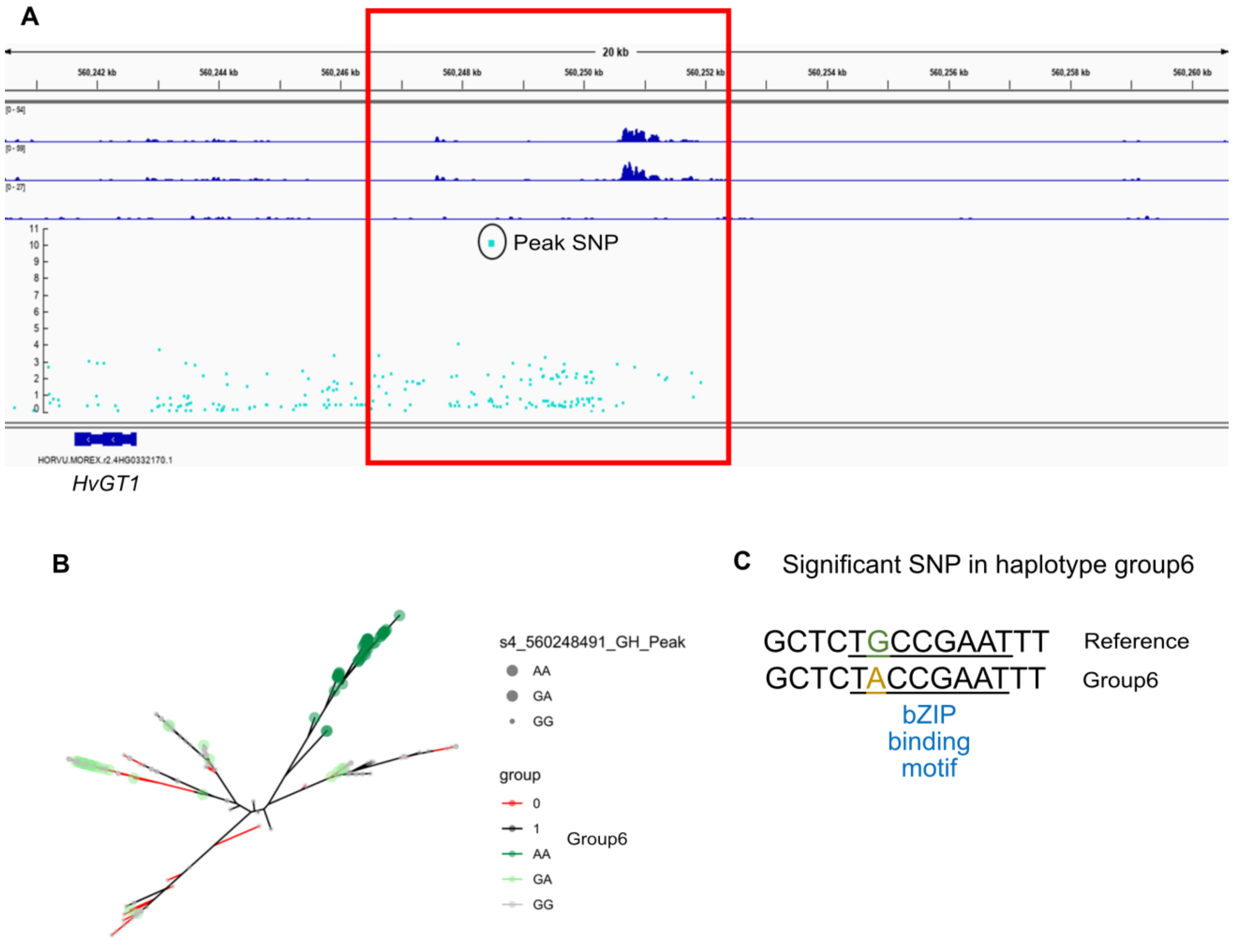


**Supplemental Figure S22. *HvGT1* natural variants enhance spikelet survival.** (Supports Figure 8)**. (A)** Significant SNPs identified within ~8-kb upstream regulatory region of *GT1* from 358 six-rowed spring barley accessions (Kamal *et al*., 2021) and 300 diverse barley accessions reported in Jayakodi *et al*., 2020*.* First and second tracks are the two replicates of the ATAC-seq data, third track is background control. **(B)** An unrooted neighbor joining phylogenic tree of *HvGT1* variants in regulatory region. **(C)** Significant SNP identified among the haplotype group 6 with high spikelet survival affects bZIP transcription factor binding cis-motif. Related data in Supplemental Data Set S40. SNP, single nucleotide polymorphism; *GT1*, GRASSY TILLERS1; bZIP, basic leucine zipper.

**Supplemental Table S1. Tentative identifications of detected molecular ions by MALDI-TOF MSI.**

| Compound class | Compound | Monoisotopic mass (Da) | Sum formula | Obtained *m/z* ± 0.1 Da | Corresponding ion(s) |
|---|---|---|---|---|---|
| Amino acids | Glutamine | 146.069 | $C_5H_{10}N_2O_3$ | 147.1 | $[M+H]^+$ |
| | Asparagine | 132.053 | $C_4H_8N_2O_3$ | 133.1 | $[M+H]^+$ |
| Sugar | Disaccharide | 342.116 | $C_{12}H_{22}O_{11}$ | 381.1 | $[M+K]^+$ |
| Tetrapyrrole | Chlorophyll *a* | 892.535 | $C_{55}H_{72}MgN_40_5$ | 893.5 | $[M+H]^+$ |
| | Pheophytin *a* | 870.565 | $C_{55}H_{72}N_40_5$ | 871.5 | $[M+H]^+$ |

## Supplemental Table S2. Primers used in this study

| **RT-qPCR** | | | |
|---|---|---|---|
| **Gene** | **MorexV3 ID** | **Forward (5'-3')** | **Reverse(5'-3')** |
| *HvNCED1* (Leymarie et al., 2008) | HORVU.MOREX.r3.5HG0426410.1 | CCAGCACTAATCGATTCC | GAGAGTGGTGATGAGTAA |
| *HvACTIN* | HORVU.MOREX.r3.5HG0457850.1 | AAGTACAGTGTCTGGATTGGAGGG | AAGTACAGTGTCTGGATTGGAGGG |
| **Subcelluar localization** | | | |
| *HvGT1-Topo* | HORVU.MOREX.r3.4HG0399240.1 | CACCATGAGCCCCGAGGAGGGAGAG | GCTGAACTGATCGTACGCCCCTC |
| **Site-directed mutagenesis/ mutational screening** | | | |
| | **Remarks** | | |
| *HvGT1_exon1gR1* | *Cas9* gRNA for *HvGT1* | TGGCGAAGCTGTCCGGGAAGACGA | AAACTCGTCTTCCCGGACAGCTTC |
| *HvGT1-1207F/1893R* | PCR amplification and mutation detection | CAGGCTCACAGACTCGGAC | CTCCTCCTCGATGAGCTTGC |
| *zCas9* | PCR amplification and mutation detection | CGGCCTCGATATTGGGACTAACTCT | CTTATCTGTGGAGTCCACGAGCTTC |
| *HptII* | PCR amplification and mutation detection | AGCTGCGCCGATGGTTTCTACAA | CATCGCCTCGCTCCAGTCAATG |
