# Supplementary material for: Multilayered regulation of developmentally programmed pre-anthesis tip degeneration of the barley inflorescence
Source: Plant Cell. 2023 Jun 7;35(11):3973–4001. doi: 10.1093/plcell/koad164 (PMC10615218; doi:10.1093/plcell/koad164)
Supplement: koad164_Supplementary_Data [file tpc22.01245Peer Review Report.pdf › tpc22.01245Peer Review Report.pdf]

Multilayered regulation of developmentally programmed pre-anthesis tip degeneration of the barley inflorescence

Nandhakumar Shanmugaraj, Jeyaraman Rajaraman, Sandip Kale, Roop Kamal, Yongyu Huang, Venkatasubbu Thirulogachandar, Adriana Garibay-Hernández, Nagaveni Budhagatapalli, Yudelsy Antonia Tandron Moya, Mohammed R. Hajirezaei, Twan Rutten, Götz Hensel, Michael Melzer, Jochen Kumlehn, Nicolaus von Wirén, Hans-Peter Mock and Thorsten Schnurbusch

Corresponding author: Thorsten Schnurbusch (schnurbusch@ipk-gatersleben.de).

Review timeline:

TPC2022-RA-01016	Submission received:<br>1st Decision:	October 17, 2022<br>November 5, 2022 *manuscript declined*
**TPC2022-RA-01245**	Submission received:<br>1st Decision:	December 22, 2022<br>March 2, 2023 *accept with minor revisions*
**TPC2022-RA-01245R1**	1st Revision received:<br>2nd Decision:<br>Final acceptance:	April 5, 2023<br>May 7, 2023 *acceptance pending, sent to sci editor*<br>June 4, 2023

REPORT: (The report shows the major requests for revision and author responses. Minor comments for revision and miscellaneous correspondence are not included. The original format may not be reflected in this compilation, but the reviewer comments and author responses are not edited, except to correct minor typographical or spelling errors that could be a source of ambiguity.)

TPC2022-RA-01016 1st Editorial decision – *declined* November 5, 2022

Thank you for submitting your manuscript entitled "Spatiotemporal multi-omic analyses of the barley inflorescence undergoing developmentally programmed pre-anthesis tip degeneration" to The Plant Cell. Your submission has been evaluated by four members of the editorial board, and we regret to inform you that we are not recommending that your manuscript in its current form proceed further in the review process.

We feel it best to not send your paper out for review until you have had sufficient time to complete this work. However, we encourage you to resubmit a revised version of your manuscript to The Plant Cell. The editors felt that the following points would need to be addressed before we could send it for review:

1. The link between the -omics analyses and GRASSY TILLERS1 is unclear. Why was this gene picked relative to others? Is there variation in GT1 sequence or expression between these two genotypes that is linked to different phenotypes? This gene is a well-known regulator of growth architecture , therefore the novelty is limited unless more relevant information is provided to link between the -omic analyses and spikelet development

2. The study of multiple genotypes was never connected. In order to make the manuscript more digestible the authors could consider presentation in a single genotype or a genotype analysis that is formally joined.

3. The analysis is limited as a result of sole consideration of 15 pairwise comparison.

4. In all - the manuscript was quite dense and difficult to digest, with the novelty of your findings being unclear. Thus, the writing could be considerably improved to address this.

-- Reviewer comments:

TPC2022-RA-01245 Submission received December 22, 2022

Editor comments on previously declined manuscript and **author responses:**

Comment 1. The link between the -omics analyses and GRASSY TILLERS1 is unclear. Why was this gene picked relative to others? Is there variation in GT1 sequence or expression between these two genotypes that is linked to different phenotypes? This gene is a well-known regulator of growth architecture, therefore the novelty is limited unless more relevant information is provided to link between the -omic analyses and spikelet development.

Authors response: We thank the editors for their suggestions. From our tissue-specific transcriptomic analysis, we identified barley *GRASSY TILLERS1 (HvGT1)* among the top 50 transcription factor genes ranked based on the apical tissue specific expression (ROKU analysis (entropy-based approach), Kadota *et al.,* 2006) (Figure 5C). Also, *HvGT1* expressed significantly higher in all three stages in the degenerating apical part compared to basal and central parts (Figure 7A). An independent transcriptome atlas of the barley inflorescence meristem with laser-capture microdissected tissues also revealed the IM dome–specific expression of *HvGT1* at the MYP stage (Thiel et al., 2021) (Supplemental Figure S10A). Interestingly, its wheat homolog, *GRAIN NUMBER INCREASE1* (*GNI1*), is expressed in rachillae and apical florets of wheat spikelets and inhibits their growth, while reduced *GNI1* expression increases floret fertility and, thus, grain number (Sakuma et al., 2019). Moreover, *GT1* orthologs in maize (Whipple et al., 2011; Klein et al., 2022) and Arabidopsis (González-Grandío et al., 2017) mediate growth repression of axillary tiller buds by ABA biosynthesis and sugar depletion. In agreement, our phytohormonal and metabolite analyses detected higher ABA levels with limited sucrose and T6P levels in degenerating apical parts, pointing to the conservation of this growth repression module during barley spike PTD. Hence, we focused on *HvGT1* as a candidate gene with a role in suppressing apical spikelet growth and regulating spike PTD.

Gene editing of *HvGT1* enhanced apical spikelet development and differentiation, substantially delayed degeneration, and increased final spikelet number in two-rowed barley (Figure 7). This result supports the evolutionarily conserved function of HD-ZIP class I family members in growth repression, as observed in grasses and eudicots (Komatsuda et al., 2007; Whipple et al., 2011; González-Grandío et al., 2017; Sakuma et al., 2017). The maize ortholog *GT1* is an essential domestication gene that suppresses carpel development in tassel florets and elongation of lateral ear branches and promotes axillary bud dormancy putatively regulated by the other domestication gene *TB1*, encoding a TCP transcription factor (Whipple et al., 2011; Dong et al., 2019; Klein et al., 2022). This domestication module in maize induces tiller bud dormancy by recruiting inhibitory phytohormones, such as ABA and jasmonate, thereby altering carbohydrate homeostasis (Dong et al., 2019). The findings in maize suggest that *HvGT1* might also function similarly in barley by modulating ABA and sugar signaling to suppress apical spikelet growth. However, barley *gt1* mutants did not show any carpel derepression phenotype in the spikelets of the non-dying region, in contrast to that seen in maize tassel florets (Klein et al., 2022). Notably, in barley, carpel suppression in lateral spikelets is controlled by the row-type *VRS* genes *(*Koppolu and Schnurbusch, 2019*)*. The *TB1* ortholog in barley, *VRS5/INT-C/HvTB1*, functions in lateral spikelet fertility/sterility, and its loss of function causes excess tillering and partial fertility in the lateral spikelets of two-rowed barley (Ramsay et al., 2011). Moreover, the expression domains of *HvTB1* and *HvGT1* were different in the barley inflorescence meristem (Supplemental Figure S10A, B), suggesting that they might function independently during barley spike growth, in contrast to maize, aside from their conserved tillering function. Thus, GT1 gained a novel function in barley in the onset of spike PTD and suppression of apical spikelet growth, most likely involving ABA pathway genes which require further downstream analysis in the *gt1* mutants.

Thus, we firmly believe, in the current version of the manuscripts, we described this in detail and made proper evidence and a better connection for choosing *HvGT1* for functional validation.

Comment 2. The study of multiple genotypes was never connected. In order to make the manuscript more digestible the authors could consider presentation in a single genotype or a genotype analysis that is formally joined.

Authors response: We thank the editors for their thoughtful suggestions. In the 'Introduction' (Lines: 76 - 81), we briefly mentioned the two major classifications of barley types based on the fertility/sterility of lateral spikelets and cited the related reference. The fertility of the two lateral spikelets in barley specifies spike row types, *viz*., two- and six-rowed barley. In six-rowed barley, all three spikelets per rachis node will be fertile and set grains, whereas both lateral spikelets will be sterile in two-rowed barley. The lateral spikelet sterility is controlled by at least five genes *VRS1, VRS2, VRS, VRS4, VRS5/INT-C/HvTB1* (Koppolu and Schnurbusch, 2019), majorly by carpel and anther abortion with growth inhibition already initiating from triple mound stage (W2.25) (Thiel et al., 2021). However, independent of this difference between two-and six-rowed barley, we found spike PTD is a common phenomenon in

both. Hence, in this study, we also investigated the developmental and molecular processes leading to spike PTD in six-rowed cv. Morex to obtain a more profound notion about this trait in both barley row types. However, we seriously considered your suggestion about the presentation of these results and made significant changes in the overall structure of the manuscript. Firstly, we presented our results pertained to two-rowed barley cv. Bowman in detail. Later, in the last result section, we briefly described the importance of including six-rowed cv. Morex in this study and presented and discussed only the significant results briefly. By presenting this way, we hope it will be more digestible for the readers.

Comment 3. The analysis is limited as a result of sole consideration of 15 pairwise comparison.

Authors response: Here, we would like to emphasize that our transcriptome analysis is not solely based on 15 pairwise comparisons. Rather it is based on the combination of tissue-specific enrichment analysis (ROKU analysis (entropy-based approach), Kadota *et al.,* 2006) and differential gene expression analysis (DESeq2) from 15 pairwise comparisons. From ROKU analysis, we identified the transcripts that are more specific to apical, central, and basal positions in all three stages. Further, we filtered the position-specific transcripts using DETs from 15 pairwise comparisons ($log_2FC > 1$; expression ≥ 1 TPM (Transcript Per Million) at least in one tissue). In addition to the above approach, we also performed Weighted Gene Co-expression network analysis (WGCNA) and derived similar conclusions. All these analyses were done independently in both barley row types (two-rowed (cv. Bowman) and six-rowed (cv. Morex)). In the current version of the manuscript, we described the results from a two-rowed cv. Bowman in detail with other biochemical and histological studies. Later, we also described the conservation of mechanism in the six-rowed cv. Morex (Figure 8). We also briefly described our approach for transcriptome analysis in the result and method sections (Line: 253 - 278). Based on your comment, we see the importance of describing our method more clearly. So we included a further detailed description of transcriptome analysis in the method section (Line: 904 - 915).

Though we utilized these independent transcriptome analyses methods considering their scopes, altogether, they pointed towards similar conclusions when describing the underlying mechanism of spike PTD. In the context of this manuscript, we gave more importance to ROKU analysis and only 15 pairwise DEG analysis. Therefore, we firmly believe that we performed detailed transcriptome analysis to list the putative candidate genes for spike PTD and proposed a model describing the molecular framework for barley spike PTD.

Comment 4. In all - the manuscript was quite dense and difficult to digest, with the novelty of your findings being unclear. Thus, the writing could be considerably improved to address this.

Authors response: We thank the editors for their suggestions. We seriously considered your suggestions and made significant improvements to the current manuscript version. We also opted to send our manuscript to professional science editors (https://planteditors.com/) and added their inputs.

TPC2022-RA-01245 1st Editorial decision – *revision requested* March 2, 2023

We have received reviews of your manuscript entitled "Spatiotemporal multi-omics analyses reveal a multilayered regulation of developmentally programmed pre-anthesis tip degeneration of the barley inflorescence." On the basis of the advice received, the board of reviewing editors would like to accept your manuscript for publication in The Plant Cell. This acceptance is contingent on revision based on the comments of our reviewers. In particular, please consider the following:

Please primarily addresss the comments of Reviewers 1 and 2; with particular focus on the phylogenetic relationship of HvGT1 to other genes; as this emphasizes the novelty of the study and the manuscript's conclusions. The multiplex CRISPR was agreed upon by all reviewers to be outside of the scope of this study.

Please highlight all changes and include a detailed annotation of changes of the text, with line numbers, and noting your responses to the comments.

-- Reviewer comments:

TPC2022-RA-01245R1 1st Revision received April 5, 2023

Editor comments on previous submission and **author responses**:

Editor comments: Please primarily address the comments of Reviewers 1 and 2; with particular focus on the phylogenetic relationship of HvGT1 to other genes; as this emphasizes the novelty of the study and the manuscript's conclusions.

Response to Reviews: First of all, we greatly appreciate editors' and referees' time and efforts on improving our manuscript with their helpful comments and suggestions.

Reviewer comments on previous submission and **author responses**:

Reviewer #1

The manuscript by Shanmugaraj et al. investigates the metabolomic and transcriptomic profiles of pre-anthesis tip degeneration (PTD) in two row and six row barley. The authors found changes in hormone concentrations (such as increased ABA, depleted auxin and cytokinin) and primary metabolites (such as low sucrose) in the dying tip of the spike compared to the surviving central spikelets that are all associated with tissue degeneration and death. The authors furthermore found that well-known regulators of leave senescence (such as NAC transcription factors) are also highly expressed in the tip of the spike. The authors then investigate in more detail the function of GRASSY TILLERS1 (HvGT1), which is upregulated in the tip, and show that in Hvgt1 full knockout mutants PTD is significantly reduced.

Overall, this is a well written and nicely presented study that will be of interest to multiple communities engaged with The Plant Cell. PTD is an important mechanism for spike yield in barley, and other major cereals, and the underlying molecular mechanisms have previously not been studied in this depth. The replication of the transcriptional and metabolic analysis in two-row and six-row barley strengthens the results of this analysis enormously. The identification of HvGT1 as an important gene in control of PTD is a valuable and novel finding of this work.

The results are presented rigorously, include extensive work, and go in depth into a major mechanism defining yield components in a staple crop. However, there are several major points which should be considered:

1. The authors introduce HvGT1 as the ortholog of TaGNI1. However, investigating the phylogeny of the two genes suggests that HvGT1 is not the direct/closest ortholog of TaGNI1. Therefore, the similarity in function (i.e., tip abortion) between the two genes is more surprising and interesting than initially presented in the study. The previously studies wheat gene GNI1 and the gene focus gene of this study (GT1) are more distant relatives then depicted in the manuscript and the direct orthologous of HvGT1 are not well studied in wheat. This provides additional novelty to the manuscript.
GT1 is distinct from the HvVRS1/wheat GNI1 clade. The manuscript would greatly benefit from a more in depth analysis and description of the phylogeny. Including a figure to clarify the relations between all mentioned orthologs (wheat, barley, maize) and connecting the two genes correctly would be important. As part of these changes, the authors could also mention the relationship between GT1 and GNI1 in the introduction (Line 135-136). GNI1 is previously mentioned in the text and thus GT1 could be connected at this point to GNI1.

We thank reviewer1 for raising this important comment. We also apologize for not making this point clearer. Anyhow, we have introduced HvGT1 as the ortholog of maize GT1, not as an ortholog of wheat TaGNI1. Rather, we mentioned HvGT1 as the homolog of TaGNI1 and we are aware based on the previous phylogeny study on HD-ZIP proteins that GT1 is distinct from VRS1 (Thirulogachandar *et al*., Plant journal, 2017). Whereas, TaGNI1 is the ortholog of VRS1. However, the reason to connect HvGT1 to GNI1 in this manuscript is to make a supportive

statement about the conserved repressive growth function of these closely related HD-ZIP proteins on distal florets/spikelets. Nevertheless, as reviewer1 mentioned, the direct ortholog of HvGT1 are not studied in wheat. Thus, to make the relationship clear, we independently generated the phylogenetic tree for VRS1 and GT1-like proteins from grasses in this study and included it in the revised version of the manuscript. Further, based on reviewer1's suggestion, we already made the changes in the introduction to relate HvGT1 (HORVU.MOREX.r3.4HG0399240.1) and TaGNI1. We hope that the changes in the revised version will help the readers to connect GT1 and other members of these HD-ZIP class I proteins (Revised MS: Lines429-441; Supplemental Figure S11). In addition, we found one duplicated GT1 in barley on Chr6H (Low confidence gene - HORVU.MOREX.r3.6HG0618240.1). However, this gene did not express in any of our spike gene expression datasets and as well as in publicly available database.

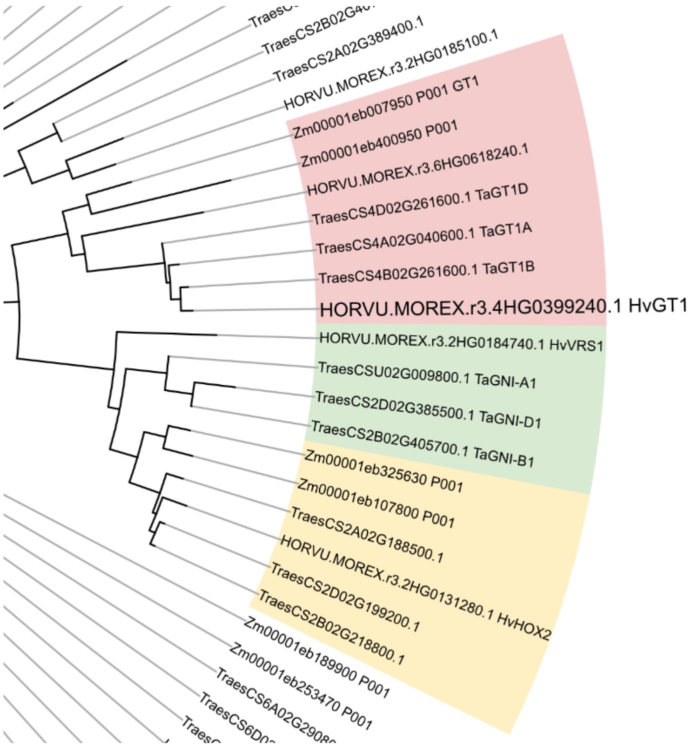

Protein sequences of GT1 and GNI1 orthologs from barley, wheat and maize were used as queries to blastp against Maize (source: Ensembl), Wheat (source: Ensembl) and barley (source: Morex v3 in IPK) protein databases with an e-value cutoff of 1e-10. This resulted in 137 non-redundant hits. Multiple sequence alignment was done using MUSCLE (REF: PMID: 15318951). Gaps present in 80% (or more) of the aligned sequences were removed using trimal (-gt 0.2) (REF: PMID: 19505945), as these gaps are considered to lack phylogenetic signals. Phylogenic tree was conducted with a Neighbor-Joining method in MEGA using the JTT model, and 1000 bootstrapped replicates.

2. The authors present the differences in metabolite compositions between the apical (dying) and central/basal (surviving) tissue. For example, W5 is described as "pre-abortion" and transcriptionally no signs of senescence are detected, the apical tissue is growing normal and developmental genes such as AP2 are expressed. However, the authors report that sucrose and T6P is already significant lower and fructose significant higher in apical compared to central tissue. Where does this place these signals in cell death? Or do these gradient in these metabolites exists across the spike at all times, with no consequences for PTD?

Mostly, they have clearly stated that differences found between the apical and central spike sections are purely correlative with PTD, however in some places a causative role has been implied and it would thus be strongly recommended that the authors adjust these sections:

We thank reviewer1 for raising these points. Transcriptionally, we see most of the changes leading to death only at W6.5 and W7.5. The gradient of sucrose and T6P in all the stages measured were found lower in the apical parts. Whereas, in central and basal parts, levels were higher compared to apical in all the stages while we saw a linear increase for sucrose across stages and an increase in T6P at W6.5, suggesting that these metabolites might be important for growth, which we found to be restricted in the developing parts. We acknowledge your recommendation and made the following changes to keep the claims rather correlative.

a. Line 187: Replace "....might be involved in..." with "accompany" (or similar)

Thanks, we have amended accordingly.

b. Line 195: replace "suggesting a poor differentiation" with "consistent with a poor differentiation"

Thanks, we have amended accordingly.

c. Line 200: replace "...pointing to..." with "consistent with"

Thanks, we have amended accordingly.

d. Line 205-208: Rephrase to avoid implying causative relations.

Thanks, we have amended accordingly.

e. Line 628: "Unequivocally demonstrates" is a very strong claim. We would suggest rephrasing to "provide another example of" or similar.

Thanks, we have amended accordingly.

f. Line 630: Replace "corroborate" with "correlate".

Thanks, we have amended accordingly.

g. Line 646-647: Rephrase as the current sentence suggests a more causative role of ABA in PTD then demonstrated by the correlative results.

Thanks, we have rephrased the concluding sentence.

3. The authors state in multiple occasions that loss of function mutants Hvgt1 increase the number of fertile florets and ultimately number of spikelets (or grains) per spike. For example, in line 137-140, line 508-509 ("In this study, we assembled a comprehensive molecular framework of spike PTD in barley that potentially affects final grain number and yield"), line 715-716, etc. However, only data showing the increase in fertile florets is presented in this manuscript. No data is presented to prove (or even support) that increased apical floret fertility translates into increased grains/spike. As the mutants were grown in multiple conditions, it is expected that this data would be relatively easy to obtain and would add valuable information to the manuscript. Even if the number of grain/spike is not increased in the Hvgt1 mutants this would still be important and interesting to show and would not affect the merits of the manuscript in any way. It would also be interesting to have number of grains/spike and number of grains/plant to understand the whole-plant effect of the Hvgt1 mutation.

We thank reviewer1 for raising this important point. We clearly understand the significance of grain number data and here is the reason why to exclude in the initial submission. As mentioned in the manuscript, we have grown azygous WT and *gt1* mutant plants in two environments. Though the final spikelet number is higher in the mutants at anthesis, the extra-surviving spikelets cannot form regular grains in most spikes investigated. They remain a rudimentary structure with empty chaff; no significant difference was found for grain number per main-culm spike between WT and *Hvgt1* mutants at harvest. This could be attributed to various reasons, such as growth conditions, genetic background, and border effect, as only a limited number of plants were grown in border rows. Hence, it is still necessary to test the yield advantage of the *gt1* mutation by growing more plants and testing the mutant effect in other genetic backgrounds. On the other hand, getting final grain yield traits gets complicated with background of two-rowed Golden promise (used for transformation) which often shows irregular ectopic spike branches in glasshouse light regime independent of mutations (as reported in several independent studies). However, such

ectopic spike branches were completely absent in the plants grown in soil in natural light (see methods). Nevertheless, what is consistent in both the environment is increased spikelet survival in *gt1* mutants. However, as reviewer1 mentioned it would be interesting and important for the readers to know the grain yield data, in this revised version we have included the grain number data per spike and discussed briefly about the outcome (Revised MS: Lines476-482, Lines667-676; Supplemental Figure S15; Supplemental Data Set S27).

4. The authors state multiple times that according to their results HvGT1 plays a role during onset of PTD (for example in line 522). However, HvGT1 is only upregulated in apical sections in W7.5 in Bowman. At this point, the tip is visibly dying and the authors stated that already in W6.5, molecular signs of PTD can be detected. Thus, HvGT1 is not upregulated at onset of PTD in Bowman. However, in Morex HvGT1 does appears to be upregulated at onset. The manuscript would benefit from addressing these differences in HvGT1 expression between the two genotypes and adding some discussion about the differences in expression. Again, this is not a major issue, but rather a more nuanced discussion of this would be best for readers.

We thank reviewer1 for their comment. In most part of the manuscript, we relate GT1 function to the onset of PTD based on the *gt1* knockout mutant phenotype in which the PTD is delayed. Our descriptions are not solely based on the expression pattern. Because, we saw an increased expression for *GT1* in the apical parts in all the stages investigated in both the genotypes. However, in Bowman it peaks at visible onset of PTD (W7.5), while in Morex it was high already at W6.5, though the visible death is delayed in Morex (W8.25). One possible reason for the increased expression of *GT1* in Morex before visible onset could be pertained to the higher spikelet abortion (absolute number) as at each rachis node it produces three spikelets compared to two-rowed Bowman. However, we still do not completely understand the reason for the delay in the visible onset of PTD in Morex. Considering the increased expression of *GT1* in all stages also suggests, they may be functioning much earlier on growth repression with subtle changes. However, we could didn't see such subtle effects in the present study with *gt1* mutants showing no obvious differences at MYP (W5.5). Rather, it is also important to consider the genotypic differences. We have included these points to convey the readers about the open questions in this direction (Revised MS: Lines458-462; Figure 7A, 8D, E; Supplemental Figure S13A).

5. The authors state that they performed a GWAS to identify SNPs identified with higher spikelet survival. However, according to the methods they only performed an association study within the region of HvGT1. This should be better described in the results section and corrected in the method section (Line 1009 and 1045). Furthermore, the data underlying this analysis is missing and should be reported as a SNP matrix with all accessions, haplotype groups and phenotypes. While acknowledging that the data is unpublished and will be part of some larger study, the 112 SNP data for the accessions is needed here to support this work. This would also allow readers to identify which accessions carry haplotype 6.

We thank reviewer1 for the comment. We performed only candidate gene association study for the region in and around *GT1*. In the revised manuscript, we clearly described this in results and method. We have included the 112 SNPs genotypic data (hapmap format), the haplotype groups and the phenotypic data in Supplemental Data Set S40.

Minor points:

1. Line 158: Add DAS information for W6.5, as reported for W7.5 in line 162

Thanks, we have amended accordingly.

2. Line 164: Replace "long before" with "long" as it is only 8 days between the two stages.

Thanks, we have rephrased a 'prior to'.

3. Line 167: Add "at W7.5 (48 DAS)" to clarify when cell death is initiated

Thanks, we have amended accordingly.

4. Line 175: Delete "endogenous".

Thanks, we have amended accordingly.

5. Line 231-236: Please cite corresponding figure rather than literature as the sentence is describing results from this publication. Please see suggested edit in track-changes.

Thanks, we have amended accordingly.

6. Line 264 and 912 in methods: Please clarify method used to classify transcripts as expressed. TPM cut-off?

Thanks for the comment. The RNA quantification was carried out using Kallisto. The quant files were then processed in R (3.4.3). Initially, for each transcript, a total number of reads mapped on all the tissue was calculated, and only transcripts with a total count greater than 100 (morex) and 150 (Bowman) were selected for differential expression analysis. So, this is based on count cut-off. The count threshold was determined based on voom transformation function from the limma package. We described these details in the method section of the revised version (Revised MS: Lines971-983).

7. Line 268-270: The highest value of DETs come from the 5.0 to 7.5 comparisons. This is strictly not a comparison across adjacent stages. If you do not consider these 5.0 to 7.5 comparisons then the Apical to Central at 7.5 is the highest one. Perhaps this could be rephrased.

Thanks for the comment. We removed this sentence in the revised version and rephrased the following sentence to describe the significant outcomes from DEG analysis.

8. Line 270-271: Reports that DETs between apical and central was higher than something but doesn't state in comparison to what

Thanks for the comment. We have rephrased the sentence.

9. Line 276-279: Suggest to remove or rephrase the last sentence of the paragraph as the conclusions stated in it do not stem directly from the above described results

Thanks, we agree with reviewer1's suggestion and have removed the last sentence, and rephrased the conclusion (Revised MS: Lines302-304).

10. Line 283: Please add how many transcripts (N= x DETs) pass the filtering steps.

By applying those filters, we found 3968 transcripts (apical – 2561, central – 387, basal – 1020). Included these numbers in the text (Revised MS: Line309)

11. From line 303 onwards: It is confusing to introduce a new nomenclature for the stages (stage 1 to 3) that overlap the previously used waddington stages and would strongly suggest to adjust the manuscript to only contain Waddington stages.

Thanks for the comment. In this revised version, throughout the manuscript we consistently followed only the Waddington scale for the descriptions of stages.

12. Line 325: Indicate that with "dying tissue" the authors refer to Waddington stage 7.5 or replace the interpretation with just the Waddington stage

Thanks for the comment. In this revised version, throughout the manuscript we consistently followed only the Waddington scale for the descriptions of stages.

13. Line 340, 342, 345, 349, 352: References are to Figure 5 but should be to figure 4.

Thanks, we have changed the figure number accordingly.

14. Line 379: replace "pre-death stage" with W6.5 or link to this Waddington stage.

Thanks for the comment. In this revised version, throughout the manuscript we consistently followed only the Waddington scale for the descriptions of stages.

15. Please include the Barley gene ID for HvGT1 somewhere in text (BaRT2v18chr4HG208140/ HORVU.MOREX.r3.4HG0399240.1) as this simplifies searching for the gene for the reader. This could be included, for example, in line 399.

Thanks, we have amended accordingly both the IDs in the results section for *GT1* mutant (Revised MS: Lines430-431).

16. Line 407: Figure S10 mention before figure S9. Also figure S9B is mentioned before S9A (which is only mentioned in discussion). Please make sure the order of figure appearance is correct.

Thanks, we have changed the order of figures according the descriptions in the text.

17. Line 408: "wheat homolog". Does this refer to wheat homolog GNI1-A or do the authors refer to GNI1 in wheat, as ortholog of HvGT1? Please revisit nomenclature.

Thanks. We refer here to wheat *GNI-A1*. We modified the text to make it clear (Revised MS: Line437).

18. Line 440: Images for heading time are only in S12A, not A and B.

Thanks, we have amended accordingly.

19. Line 463, 473, 975: Capital "A" for figure numbering.

Thanks, we have amended accordingly.

20. Line 485: Please add P-value cut-off information for overlap in genes.

Thanks, we have amended accordingly.

21. Line 490-495: The authors quote "358" accessions have been used, however in Figure S18 they quote 102+508 accessions have been used. Please clarify how many accessions were used in association study.

Thanks for the comment. We apologize for the mistake in Fig. S18. For the association study, we used 358 spring barley accessions. In addition to 358 barley accessions, we included SNPs in the same interval from 300 diverse barley accessions reported elsewhere (Jayakodi et al., 2020). We modified the figure legend (Revised MS: figure S22).

22. Line 527-529: The authors cite one definition of senescence, however senescence can occur independent of age or of growth cessation. Perhaps this statement is not required?

Thanks for the comment. We completely accept reviewer1's suggestion. As we would like to categorize the PTD in certain form of senescence, we rephrased the introduction of this discussion part in the revised version (Revised MS: Lines577-581).

23. Line 614: Replace "significance" with "possibility" to clarify that effect of PTD on yield has not been investigated in this study.

Thanks, we have amended accordingly (Revised MS: Line665).

24. Line 893: Daghma in lower case.

Thanks, we have amended accordingly.

25. Line 914: Delete space after bracket.

Thanks, we have amended accordingly.

26. Line 955: GO enrichment analysis: The background set against which enrichment was done should be reported. This should be against expressed genes in the dataset and not against all barley genes.

Thanks for the comment. We used DETs from pairwise comparisons as well as DETs from tissue-specific ROKU analysis for GO enrichment. We rephrased and clarified this in the corresponding method section (Revised MS: Line1017-1024).

27. Line 993: Supplemental table S16 does not exist in supplemental material. Please correct.

Thanks, we have corrected it.

28. Line 993: It is not clear from the text how the authors have 29 events with 41 sister lines. Are these 41 sister lines per event? Or do most of the 29 events have 1-2 sister lines? Please clarify how many sister lines existed per event.

We apologize for the confusion. The reason is some of the 29 events have 1-2 sister lines. An event is from one callus. Some events have more than one sister lines.

29. Line 1001, 1011: Add "accessions" after barley (or appropriate description).

Thanks, we have amended accordingly.

30. Line 1016: Replace "done" with "performed"

Thanks, we have amended accordingly.

Data availability:

31. Data availability: Please deposit mutant germplasm material.

***gt1* mutant used in this study are developed by CRISPR/Cas9 technology and can be provided under request.**

32. Data availability: Please deposit the full expression matrix for expressed genes/transcripts (data used for PCA analysis in Figure 4). Please also consider reporting PCs 3 to 6 by including plots of these against PC1 in a supplemental figure. It would also be interesting to see how libraries cluster when the data is split into individual sections or Waddington stages as the samples from W7.5 are very different and lead to all other libraries clustering closely together. Please consider including these alternative PCA plots.

Thanks, we have amended accordingly. We provided the expression matrix used for PCA in the supplemental dataset. Additionally, PCA plots until PC6 is also added in the supplemental figures (Supplemental Figure S4A; Supplemental Datasets S3, S4).

Figures:

33. Figure 1, line 1056: Please indicate how many experiments (N =)

Thanks, we have amended accordingly.

34. Figure 5: Perhaps include total number of each TF family in barley genome to give a sense of how "enriched".

Thanks for the comment. We haven't separately performed TF enrichment analysis. Rather the TF are selected based on GO terms enriched for 'transcription' and also based on barley annotation. To avoid confusion, we changed the text from 'enriched' to 'apically expressed' in the main-text (Revised MS: Line399) as well as in the figure (Figure 5A).

35. Figure 6: Legend described "expression shapes of death modules", however only one module is shown so legend should be adjusted.

Thanks, we have amended accordingly.

36. Figure 7C: In legend, please mentioned how many spikes were analyzed per event (N =).

Thanks, we have amended accordingly.

37. Figure S17 is not mentioned in text, thus figure S18 is mentioned before S17.

Thanks, we have amended accordingly. Previously, we mentioned these data for the discussion. In this revised version (Line508), we mention it in the main results.

38. Figure S3A: Why does the spike have much fewer (maybe 27) nodes than indicated in dataset S1 and figure 1 (at 7.5, bowman has 39 nodes)

Thanks for the comment. MALDI MSI was conducted in the divided spikes, as they are big enough to make optimal longitudinal sections due to technical difficulties. We mentioned in the figure clearly the section starts from node 19 and pointed with the arrow.

39. Figure S3B: Color scheme could be improved for clarity

Thanks for the comment. We have amended accordingly.

40. Figure S9B: The blue colour obscures the actual +1 polymorphism, so perhaps a lighter colour would be better.

Thanks for the comment. Unfortunately, it is the default color in the tool used (Revised MS: figure S12).

41. Figure S10D: The comparison of WT to mutant could be aided by zooming in a little more and by adding to the images which node number the spikelet nodes correspond to.

Thanks for the comment. We have amended accordingly (Revised MS: figure S13C).

42. Figure S10E: Missing scale.

Thanks for the comment. We have excluded that result in this revised version (Revised MS: figure S13).

43. Figure S11: To better compare between WT and mutant the corresponding spikelet nodes, it would be very useful to have numbers next to detached spikelet pictures that indicate which node they came from. Please also "tidy" bottom panel ("extra survived"change to "extra surviving", arrows for stamen are difficult to see and should point arrow the other way).

Thanks for the comment. We have modified the (Revised MS: figure S14) as suggested.

44. Figure S18: Legend is not clear. Top two tracks are Open-chromatin based on ATAC-Seq? The SNP track is not clear as the blue is very faint when printed. The bZIP motif SNP should be clearly highlighted in panel A. In panel A perhaps label gene as GT1 as it is assumed but not clear.

Thanks for the comment. First and second tracks are the two replicates of the ATAC-seq data, third track is background control. Raw data is from PMID: 31740772. We have modified the figure as suggested (Revised MS: Figure S22).

Reviewer #2:

This manuscript by Shanmugaraj et al. describes an interesting and novel aspect of barley inflorescence development and fertility related to the degeneration of the apical tip of the developing spike. This trait has links to other major cereals such as rice and maize that form indeterminate inflorescence meristems, and knowledge about the genes that promote tip degeneration could benefit breeding by providing new targets for enhancing yield. I therefore congratulate the authors on addressing a novel aspect of inflorescence development using an innovative approach.

On the whole, the manuscript is well-written and the results have been presented in clear figures that support their conclusions. The authors have employed an impressive array of techniques to test their hypotheses, and the research has been arranged in a logical order that leads to a well-supported conclusion that GRASSY TILLERS1 is a key gene promoting tip degeneration in barley.

I did not review the original manuscript but have been asked to assess a revised version and the authors' responses to comments of a reviewer. I believe the authors have answered all concerns sufficiently. Having said this, there is an important concern outlined below that was not raised by the previous review that I

think is crucial for the authors to answer and address, especially for placing this research in the context of published work on barley inflorescence development.

1. I'm confused about the authors' introduction of HvGT1 and its relationship to VRS1. I understood from their published work on GNI1 in wheat that GNI1 was an orthologue of VRS1, and yet here, the text links GNI1 and HvGT1 function, but not VRS1. This leads to the question, are HvGT1 and VRS1 the same gene? If so, then I strongly encourage the authors to be more transparent about this point throughout the manuscript, as if this is the case, then the results raise interesting points that should be discussed. For example, why do the gene edited lines have a 2-row spikelet architecture if GT1/VRS1 has been knocked-out (Figure S11)? And if VRS1 is the major gene controlling 2-row vs 6-row architecture, and GT1 (VRS1?) is a key regulator of PTD, why do 2-row and 6-row types share PTD as a common phenomenon? In my opinion, the lack of clarity about HvGT1 and VRS1 gene identity would be a major source of confusion for the field, and so this should be addressed by the authors.

We thank reviewer2 for raising this important comment. We also apologize for not making this point clearer. We addressed this point on the relationship between GT1, VRS1, and GNI1 in the revised manuscript with the help of phylogenetic analysis between these HD-ZIP I proteins from grasses. GT1 in barley is not a row-type gene like VRS1. Rather, GT1 has a distinct role in regulating spike PTD. Therefore, that is the reason why we do not see any phenotype in the lateral spikelets of two-rowed CRISPR knockout lines. Further, we made the changes in the introduction to relate HvGT1 and TaGNI1. Hope the changes in the revised version help the readers to connect the GT1 and other members of these HD-ZIP class I proteins (Revised MS: Lines429-441; Supplemental Figure S11).

2. In the description of the transcriptome analyses, there is much discussion about the enrichment of genes from ontologies at the different developmental stages, but it is only in a few instances that the direction of changes in gene expression is mentioned. For example, are enriched genes down or upregulated in PTD tissue relative to living tissue? Can the authors provide more text to describe the direction of gene expression in the different tissues/developmental stages?

We thank reviewer2 for the comment. Initial brief description of transcriptional changes are based on DEG analysis from 15 pairwise combinations that includes both up/down-regulated transcripts just to give overall impression on changes across and within stages (Fig. 4B. shown a representative GO enrichment for UP/DOWN DETs between dying apical and viable central parts within stages). However, the major results presented regarding transcriptome data are based on tissue-specific enrichment analysis (ROKU method), in which we describe the transcripts enriched (upregulated) in specific tissue-type, say apical, central and basal. So here, the GO are based on these enriched (upregulated) genes in apical, central and basal parts across stages. In addition, based on reviewer1's comment, we made some changes in the text (Revised MS: Lines292-299; 305-310; Method – Lines1017-1024). Hope we have made this clear.

3. L394-395: The authors mention that light regulation may be important for spike PTD, but the developmental stages of PTD occur when the spike is still in the boot. How then would light influence PTD?

We thank reviewer2 for the comment. The description about the possibility of light regulations on PTD in this manuscript is based on the higher expression of certain light signaling receptors and circadian clock genes. Further, previous study showed the changes in photoperiod impacts spikelet fertility and growth (Diegel et al., The Plant Cell, 2015). Recent research work from our group also found the presence of vascular-specific clock within the spike that might regulate spikelet growth and PTD (Huang et al., 2023, Sci Adv.). Indeed, we cited these references to support our statement in the discussion. However, the effect of light on these hidden organs are still not clear and hypothetical, but the possibilities can also the signals derived from other parts (like leaves).

Minor comments

1. In the keywords, should "PCD" be "PTD" or are the authors referring to programmed cell death?

Thanks. We are referring to Programmed cell death.

2. Line616 - should "undirected" be "indirect"?

Thanks. 'Undirected' is used to represent the coexpression network constructed. This is the standard phrase used in WCGNA or GENIE3.

3. L606 - in relation to the point about excessive tillering, I recommend the authors review this statement based on a more recent publication by Zwirek et al. 2019.

We thank reviewer2 for the comment. We rephrased in the revised MS. (Revised MS: Lines655-657).

Reviewer #3:

While I appreciate the extensive efforts the authors have undertaken to describe the molecular changes associated with PTD, I feel that the initial editorial comment that the novelty is limited unfortunately still holds. If the authors would illustrate a reproducible manner in which these combined data can be used to identify solutions to this problem, that would be a major step forward. However, while there are many and obvious connections to the validation target they chose, a step-by-step selection of this target based on the data is not available. It is therefore not strongly novel to select and validate, based on the data available in the literature and a ranking of candidate targets based on enrichment in the process. If a reproducible selection process would be used to identify and validate unexpected targets, both novel insight, a methodological advance and potentially novel biological processes would have been discovered. My opinion therefore is that if the authors want to have the manuscript considered for a high-impact journal, they should start at the top of the list probably and validate not one but more than one target, preferably the unanticipated ones rather than the anticipated. A descriptive approach, though no doubt state-of-the-art, remains descriptive in nature and is very worthwhile, but in particular so when it leads to discovery. There are very appropriate current manners to do this. Use for example a multiplex CRISPR construct to generate multiple knockouts in a single round of transformation and genotype and phenotype. In such an approach, it remains important to start from a well considered and preferably reproducible selection process of the targets selected to validate.

TPC2022-RA-01245R1 2nd Editorial decision – *acceptance pending* **May 7, 2023**

We are pleased to inform you that your paper entitled "Spatiotemporal multi-omics analyses reveal a multilayered regulation of developmentally programmed pre-anthesis tip degeneration of the barley inflorescence" has been accepted for publication in The Plant Cell, pending a final editorial review by a science editor. At this stage, your manuscript will be evaluated by a science editor with respect to its presentation of scientific content, compliance with journal policies, and presentation for a broad readership.

Final acceptance from Science Editor **June 4, 2023**
